# Cationic Gold(II) Complexes: Experimental and Theoretical Study**

**DOI:** 10.1002/chem.202201794

**Published:** 2022-09-01

**Authors:** Jaya Mehara, Adarsh Koovakattil Surendran, Teun van Wieringen, Deeksha Setia, Cina Foroutan‐Nejad, Michal Straka, Lubomír Rulíšek, Jana Roithová

**Affiliations:** ^1^ Department of Spectroscopy and Catalysis Institute for Molecules and Materials Radboud University Nijmegen Heyendaalseweg 135 6525AJ Nijmegen (The Netherlands; ^2^ Institute of Organic Chemistry and Biochemistry Czech Academy of Sciences Flemingovo náměstí. 2 16610 Prague Czech Republic

**Keywords:** density functional calculations, electronic spectroscopy, gold, mass spectrometry, vibrational spectroscopy

## Abstract

Gold(II) complexes are rare, and their application to the catalysis of chemical transformations is underexplored. The reason is their easy oxidation or reduction to more stable gold(III) or gold(I) complexes, respectively. We explored the thermodynamics of the formation of [Au^II^(L)(X)]^+^ complexes (L=ligand, X=halogen) from the corresponding gold(III) precursors and investigated their stability and spectral properties in the IR and visible range in the gas phase. The results show that the best ancillary ligands L for stabilizing gaseous [Au^II^(L)(X)]^+^ complexes are bidentate and tridentate ligands with nitrogen donor atoms. The electronic structure and spectral properties of the investigated gold(II) complexes were correlated with quantum chemical calculations. The results show that the molecular and electronic structure of the gold(II) complexes as well as their spectroscopic properties are very similar to those of analogous stable copper(II) complexes.

## Graphical abstract

**Gilded gases**: Gold(II) complexes are rare, and a little is known about their properties. We have generated gaseous gold(II) halido complexes from their gold(III) precursors with various auxiliary ligands and investigated them by helium‐tagging infrared and visible photodissociation spectroscopy. The structure of the complexes was described by DFT calculations benchmarked by comparison with the experimental data.

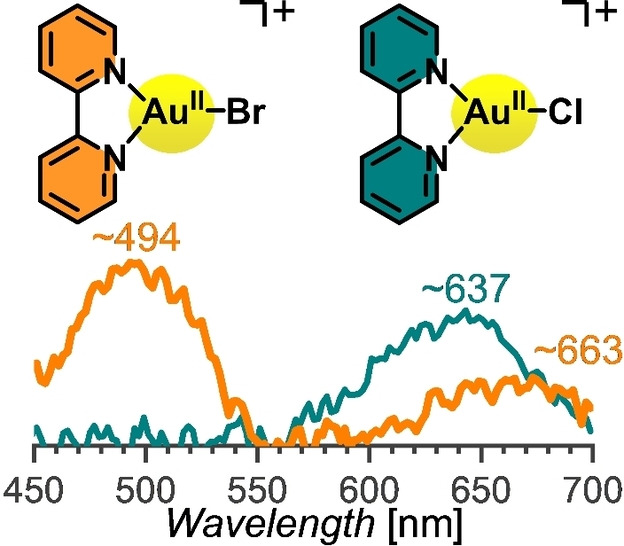

## Introduction

Gold(I) and gold(III) complexes have an important place in the field of metal catalysis.[^1^, ^2^, ^3^, ^4^, ^5^, ^6^, ^7^, ^8^, ^9^] Gold(I) complexes are mainly used to activate multiple bonds towards additions and subsequent cascade reactions^[10]^ whereas gold(III) complexes have been used as catalysts in various coupling reactions.[^11^, ^12^, ^13^, ^14^, ^15^] Conversely, reports on gold(II) chemistry are sparse.[^16^, ^17^, ^18^, ^19^, ^20^, ^21^, ^22^] The gold(II) complexes are usually unstable and tend to disproportionate to the gold(I) and gold(III) complexes.^[23]^ The destabilization of the gold(II) oxidation state is due to the relativistic effects[^24^, ^25^] which are responsible for an expansion and energy destabilization of the 5d shell and contraction of the 6 s electron shell.[^24^, ^26^] As a result, the contribution of the 5d electrons to the valence shell increases. The ionization of 5d electrons is then associated with the Jahn‐Teller distortion which leads to a large energy splitting of the d${}_{z{}^{2}}$ and d${}_{x{}^{2}-y{}^{2}}$ energy levels. Thus, gold is easily ionized to the gold(III) state with all electrons paired.^[27]^

Heinze and co‐workers reported a series of stable monomeric gold(II) complexes supported by porphyrin ligands.[^20^, ^28^, ^29^] The preparation of the gold(II) complexes relied on the reduction of the corresponding gold(III) precursors. In order to avoid the subsequent reduction to gold(I), the authors used cobaltocene (*E*_1/2_=−1.3 V vs. Fc/Fc^+^ couple) which matched the redox potential for the first reduction potential of the gold(III) porphyrin complex.^[20]^ The only reactivity of prepared gold(II) complexes studied so far was based on electron transfer processes.^[20]^ However, they were suggested as intermediates in several reactions involving radical chemistry.[^15^, ^30^] The reactions beyond the electron transfer require a labile coordination site at the gold(II) complexes that could coordinate reactive molecules or radicals. Therefore, we aimed at exploring the generation and properties of gold(II) complexes with bidentate and tridentate ligands.

Previous electrochemical experiments with analogous complexes showed that the reduction of gold(III) complexes often leads directly to the gold(I) complexes or that the Au^III^→Au^II^ and Au^II^→Au^I^ potentials are very close and therefore the steps are difficult to separate.[^31^, ^32^, ^33^, ^34^] In such a situation, where the properties of reactive complexes cannot be properly studied in solution, isolation of these species in the gas phase can extend their lifetime and thus allow a detailed study in vacuum.[^35^, ^36^] This approach permitted an exploration of the chemistry of metal complexes in unusual oxidation states or even in the oxidation states that cannot be prepared in a condensed phase.^[37]^ In addition, previous DFT calculations suggested that the gold(II) complexes are more stable in the gas phase than in the solution.^[23]^

Herein, we present a gas‐phase study of the intrinsic properties of gaseous gold(II) complexes with bidentate and tridentate ligands. We generated the gold(II) complexes from their gold(III) precursors and explored how various common ligands stabilize the unusual oxidation state. We have characterized the generated gold(II) complexes with gas‐phase ion spectroscopy. We have also investigated their structure and spectroscopic properties by DFT calculations.

### Results

#### Generation of the gaseous complexes

Our aim has been to explore the properties in terms of thermodynamic stability and spectral properties in IR/VIS range of cationic gold(II) complexes in the gas phase. We envisaged that the easiest general way of generation of these complexes could start from the [Au^III^(L)_*n*_(X)_2_]^+^ precursors (*n*=1 for bidentate ligands and *n*=2 for monodentate ligands such as pyridine and PPh_3_) that might be prone to lose a halogen radical and thereby form the desired [Au^II^(L)_*n*_(X)]^+^ complexes (Reaction 1). In the study of the gold(III) complexes, we indeed observed this reaction path. However, we also observed competing reactions leading to the reduction of the complex to gold(I) (Reaction 2) or to the degradation of the gold(III) complex (Reaction 3.

(1) $$\left[\mathrm{Au}{}^{\mathrm{III}}\left(L\right)\left(X\right){}_{2}\right]{}^{+}\to \left[\mathrm{Au}{}^{\mathrm{II}}\left(L\right)\left(X\right)\right]{}^{+}+X{}^{•}$$

(2) $$\left[\mathrm{Au}{}^{\mathrm{III}}\left(L\right)\left(X\right){}_{2}\right]{}^{+}\to \left[\mathrm{Au}{}^{I}\left(L\right)\right]{}^{+}+X{}_{2}$$

(3) $$\left[\mathrm{Au}{}^{\mathrm{III}}\left(L\right)\left(X\right){}_{2}\right]{}^{+}\to \left[\mathrm{Au}{}^{\mathrm{III}}\left(L\text{-}H\right)\left(X\right)\right]{}^{+}+\mathrm{HX}$$

We tested the fragmentations of [Au^III^(L)_*n*_(X)_2_]^+^ with a series of monodentate‐, bidentate‐ and tridentate ligands with N, P, and S coordinating atoms (Table 1). The monodentate ligands do not support the desired formation of gold(II) complexes. The reason stems from the coordination flexibility of the monodentate ligands in the coordination sphere of the gold atom. The gold(III) precursors were formed in a square planar geometry[^14^, ^38^, ^39^] with the [Au^III^(η^1^‐ligand)_2_(X)_2_]^+^ speciation. These complexes can easily eliminate the dihalogen molecule (X_2_) because the resulting [Au^I^(η^1^‐ligand)_2_]^+^ can attain the favoured linear arrangement of the two η^1^‐ligands and thereby favour Reaction 2.[^33^, ^40^, ^41^, ^42^, ^43^, ^44^] Dicationic gold(II) complexes with neutral monodentate ligands can be generated in the gas phase by reaction of gold atoms with neutral ligands and subsequent electron ionization of the complex. Properties of the [Au^II^(η^1^‐ligand)_*n*_]^2+^ generated in this way were studied previously.[^45^, ^46^]

**Table 1:** Fragmentation of [Au^III^(ligand)(X)_2_]^+^ complexes.^[a,b]^

		Branching of fragmentations pathways^b^			
		Reaction 1	Reaction 2	Reaction 3	Other
Ligand	X	[Au^II^(L)(X)]^+^+X⋅	[Au^I^(L)]^+^+X_2_	[Au^III^(L−H)(X)]^+^+HX	(specification of the fragment(s))
Pyridine	Cl	–	18 %	66 %	16 % (X_2_; pyridine loss): complex reduced to gold(I)
Pyridine	Br	–	94 %	–	6 %
TMEDA	Cl	**13 %**	–	8 %	79 % ([Au(H)(Cl)_2_] loss)
TMEDA	Br	**98 %**	–	2 %	–
2,2’‐Bipyridine	Cl	**93 %**	6 %	–	1 %
2,2’‐Bipyridine	Br	**100 %**	–	–	–
2,2’‐Bipyridine	I	**99 %**	–	–	1 %
1,10‐Phenanthroline	Cl	**93 %**	7 %	–	–
1,10‐Phenanthroline	Br	**100 %**	–	–	–
2,2’:6’,2’‘‐Terpyridine	Cl	**85 %**	14 %	–	1 %
2,2’:6’,2’‘‐Terpyridine	Br	**96 %**	2 %	–	2 %
PPh_3_	Cl	Precursor complex [Au^III^(PPh_3_)_2_(Cl)_2_]^+^ not observed			
PPh_3_	Br	Precursor complex [Au^III^(PPh_3_)_2_(Br)_2_]^+^ not observed			
Ph_2_P‐(CH_2_)‐PPh_2_ (dppm)	Cl	Precursor complex [Au^III^(dppm)(Cl)_2_]^+^ not observed			
Ph_2_P‐(CH_2_)‐PPh_2_ (dppm)	Br	Precursor complex [Au^III^(dppm)(Br)_2_]^+^ not observed			
Ph_2_P‐(CH_2_)_2_‐PPh_2_ (dppe)	Cl	–	–	2 %	98 % (loss of PhCl and subsequent ligand degradation)
Ph_2_P‐(CH_2_)_2_‐PPh_2_ (dppe)	Br	**63 %**	–	–	37 % (loss of PhBr and subsequent ligand degradation)
Ph_2_P‐(CH_2_)_3_‐PPh_2_ (dppp)	Cl	–	–	5 %	95 % (subsequent ligand degradation)
Ph_2_P‐(CH_2_)_3_‐PPh_2_ (dppp)	Br	**8 %**	16 %	–	76 % (subsequent ligand degradation)
Ph_2_P‐(CH_2_)_4_‐PPh_2_ (dppb)	Cl	–	–	15 %	85 % (loss of 2 x HCl and subsequent ligand degradation)
Ph_2_P‐(CH_2_)_4_‐PPh_2_ (dppb)	Br	Precursor complex [Au^III^(dppb)(Br)_2_]^+^ not observed			
Phenylpyridine	Cl	Precursor complexes [Au^III^(phenylpyridine)_2_(Br)_2_]^+^ or [Au^III^(phenylpyridine‐H)(Br)]^+^ not observed			
Thiophene	Br	Precursor complex [Au^III^(thiophene)_2_(Br)_2_]^+^ not observed			
2,2’‐Bithiophene	Br	Precursor complex [Au^III^(2,2’‐thiophene)(Br)_2_]^+^ not observed			

[a] The complexes were generated from an acetonitrile and dichloromethane solution of the AuX_3_ salt (0.1 mM) and the ligand (0.1 mM) achieving a final concentration of 100 μM. [b] The fragmentations were investigated at a collision energies of 1.9–3.9 eV

**Figure 1:**
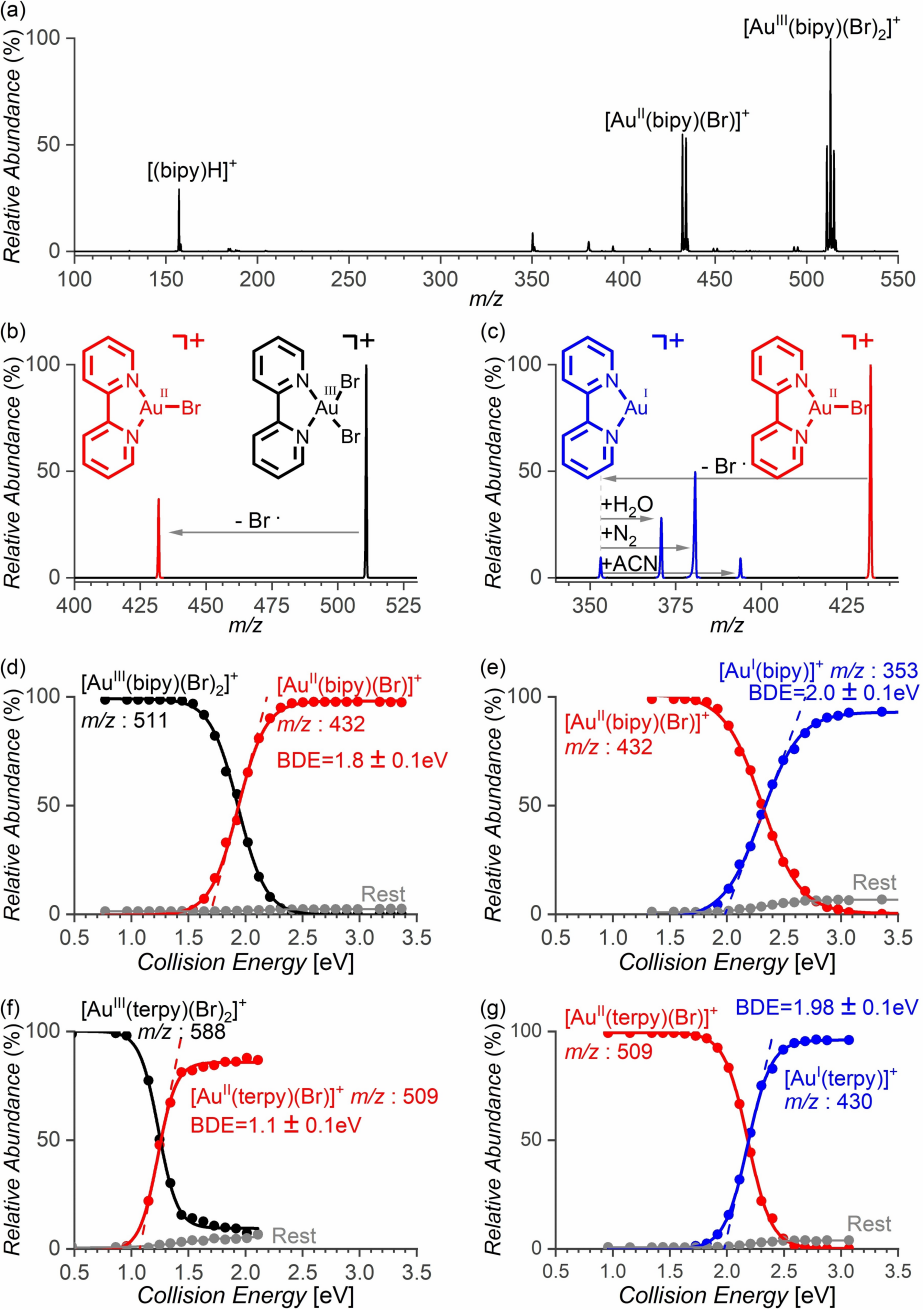
a) Electrospray ionization mass spectrum of a solution of 2,2’‐bipyridine (bipy, 100 μM) and AuBr_3_ (100 μM) in ACN. Collision‐induced dissociation (CID) spectra of mass‐selected b) [Au^III^(bipy)(Br)_2_]^+^ (*m/z* 511) at a collision energy of 1.9 eV and c) [Au^II^(bipy)(Br)]^+^ (*m/z* 432) at a collision energy of 2.4 eV. Integrated abundances of the peaks in the energy‐resolved CID spectra of the ions with d) *m/z* 511 and e) *m/z* 432. Integrated abundances of the peaks in the energy‐resolved CID spectra of f) [Au^III^(terpy)(Br)]^+^ (*m/z* 588) and g) [Au^II^(terpy)(Br)]^+^ (*m/z* 509). Extrapolation of the onset of the fragmentation provides the BDE for the given fragmentation (see also Figures S4, S6, S14, S16, S19 and S21 in the Supporting Information).

In contrast, the bidentate ligands do support the formation of gold(II) complexes. The gold(III) precursors were formed with the [Au^III^(η^2^‐ligand)(X)_2_]^+^ speciation.^[47]^ Fragmentation of the gold(III) precursors with N,N’‐bidentate ligands led almost exclusively to the elimination of a halogen radical and thus to the formation of the desired [Au^II^(η^2^‐ligand)(X)]^+^ complexes (Reaction 1, Figure 1b). In comparison, phosphine‐based ligands are less suitable for the generation of gold(II) complexes because they get easily oxidised in the presence of the gold(III) salts. Accordingly, next to the desired gold(III) precursors with the bis‐phosphine ligands (*P,P’‐*ligands), we have always observed gold(I) complexes with the bis‐phosphine‐oxide ligands. Nevertheless, we have detected a fraction of the desired [Au^III^(*P,P’*‐ligand)(X)_2_]^+^ precursors with the bis‐diphenylphosphine ethylene (dppe) and propylene (dppp) ligands and therefore we could explore the possibility to form the gold(II) complexes.^[48]^ The desired reaction 1 occurred only for complexes with X=Br; hence, we observed the formation of [Au^II^(dppe)(Br)]^+^ and [Au^II^(dppp)(Br)]^+^. The competing reactions led generally to the reduction of the complexes to the gold(I) state and to a degradation of the ligands (see Table 1, and the Supporting Information). The bis(diphenylphosphino)methane (dppm) ligand with a smaller bite angle provided directly just gold(I) complexes. We also tested sulphur‐based ligands but were unable to prepare the desired gold(III) precursor complexes.

Finally, we tested the same approach with a N,N’,N’’‐tridentate ligand (1^2^,2^2^:2^6^,3^2^‐terpyridine, terpy in the following). The precursor gold(III) complexes had a [Au^III^(terpy)(X)_2_]^+^ speciation. As the gold(III) complexes prefer square planar coordination and the complex retains two halogen ligands, the terpy ligand initially coordinates probably only as a bidentate ligand. The complex easily eliminates one of the halogen atoms and forms [Au^II^(terpy)(X)]^+^. Presumably, the ligand changes the coordination mode and fills in the vacant coordination site after the elimination of X. For more detailed information, we studied bond dissociation energies (BDEs) for the halogen eliminations.

#### Energetics of gold(II) complex formations and fragmentations

**Figure 2:**
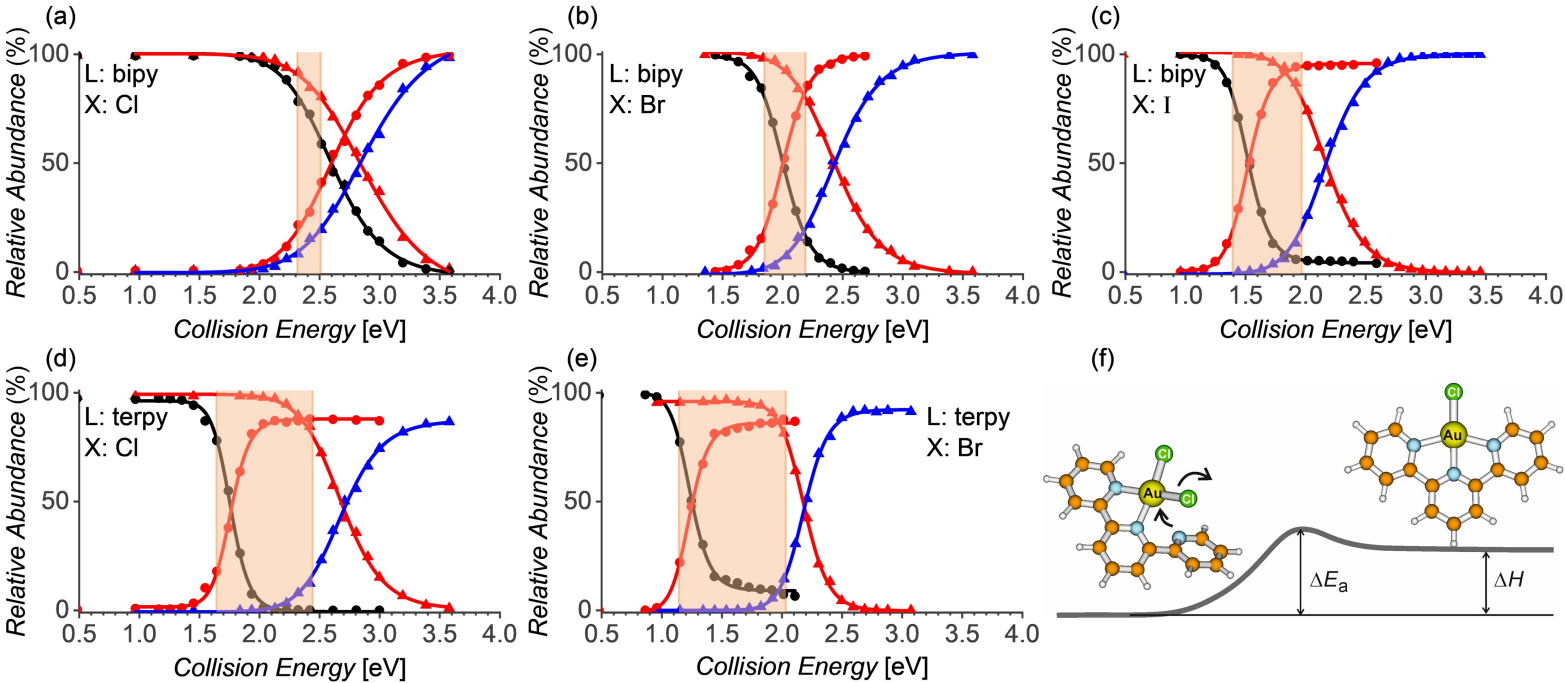
Energy‐resolved CID curves for the dissociation of [Au^III^(L)(X)_2_]^+^ (black) to [Au^II^(L)(X)]+ (red) and [Au^II^(L)(X)]^+^ (red) to [Au^I^(L)]^+^ (blue); L=bipy (a–c) and terpy (d, e); X=Cl (a, d), Br (b, e) and I (c). The orange regions depict the energy window for the possible generation of the gold(II) complexes. The window starts when 20 % of the gold(III) complexes fragment to the gold(II) complexes and stops when 20 % of the gold(II) complexes fragment to the gold(I) complexes. f) Schematic potential‐energy profile for the elimination of Cl⋅ from [Au^III^(terpy)Cl_2_]^+^; the depicted structures are optimized at the B3LYP‐D3BJ/6‐311*G**/SDD(Au) level of theory.

A comparison of the results for various complexes suggests that the bromido ligands are more suitable for the generation of the gold(II) complexes than the chlorido ligands. We have investigated this in a more detail using complexes with the 2,2’‐bipyridine ligand and we have also included the iodido precursors. The aim was to determine the span of the energies that are sufficient to induce the reduction from [Au^III^(bipy)(X)_2_]^+^ to [Au^II^(bipy)(X)]^+^, but insufficient to induce the second reduction step from [Au^II^(bipy)(X)]^+^ to [Au^I^(bipy)]^+^ (Figure 2, Table 2). Note that the gold(III) iodine complexes easily undergo a disproportionation reaction.[^49^, ^50^] Therefore, we had to generate the [Au(bipy)(I)_2_]^+^ complexes in situ by the Finkelstein reaction[^51^, ^52^] from the corresponding chloride complex and sodium iodide.

**Table 2:** Experimental^[a]^ and theoretical^[b]^ bond dissociation energies of [Au(L)(X)_*n*_]^+^ complexes (*n*=1 or 2).

		[Au^III^(L)(X)_2_]^+^ → [Au^II^(L)(X)]^+^+X⋅		[Au^II^(L)(X)]^+^ → [Au^I^(L)]^+^+X⋅			
X	L	BDE_exp_^[a]^ [kJ mol^−1^]	BDE_theor_^[b]^ [kJ mol^−1^]	BDE_exp_^[a]^ [kJ mol^−1^]	BDE_theor_^[b]^ [kJ mol^−1^]	ΔBDE_exp_ [kJ mol^−1^]	ΔBDE_theor_ [kJ mol^−1^]
Cl	bipy	213± 1	225	229 ± 2	244	16	19
Br	bipy	169± 5	199	196 ± 4	231	27	32
I	bipy	128± 2	174	177 ± 2	221	49	47
Cl	terpy	155± 3	131	226 ± 1	232	71	101
Br	terpy	108± 6	113	191 ± 1	210	83	97
							
		[Au^III^(L)(X)]^2+^ → [Au^II^(L)]^2+^+X⋅					
Cl	terpy	185± 4	259				
Br	terpy	165± 4	237				

[a] The experimental values were determined from energy‐resolved CID experiments (see Experimental and S4, S6, S9, S11, S14, S16, S19, S21, S23, S26, S28 and S30). [b] The theoretical values were calculated by using Gaussian 16: B3LYP−D3BJ/6‐311+G** level with SDD/SDD‐ECP on Au and 6‐311G** on I. See also Table S1 for results obtained at the PBE0‐D3/def2TZVPP level of theory. These results show the same trend, but overestimate the binding energies even more.

The energy demands for the reduction reactions were determined from energy‐resolved collision‐induced dissociation experiments with mass‐selected [Au^III^(bipy)(X)_2_]^+^ and [Au^II^(bipy)(X)]^+^ (Figures 1 and 2). The extrapolation of the energy‐dependent relative fragmentation cross‐section for the elimination of the halogen atom provides the experimental Au−X bond‐dissociation energy (see the Experimental Section). The energy difference between the energy demands for the Au^III^→Au^II^ and Au^II^→Au^I^ reductions decrease in the order of X: I>Br>Cl (Table 2). Hence, the iodido complexes provide the largest interval of energies that allow the formation of the gold(II) complexes from their gold(III) precursors. However, with respect to the low stability of the gold(III) iodido precursors, the bromido complexes are probably the best candidates for studying gold(II) complexes in the gas phase.

We have also studied BDEs for the complexes with the tridentate ligand terpy and X=Cl and Br. The dominant gold(III) complexes detected from the solution correspond to the [Au^III^(terpy)(X)]^2+^ dications. The energy required for the formation of [Au^II^(terpy)]^2+^ is slightly smaller than that for the formation of [Au^II^(bipy)(X)]^+^ (the bottom of Table 2). This attests that a pyridine type ligand better stabilizes the +II oxidation state of gold than the chlorido ligand. Interestingly, this trend is not caught by the DFT calculations (see also below).

The gold(III) complexes with the terpy ligand and two chlorido ligands, [Au^III^(terpy)(X)_2_]^+^, can have two possible structures. The first structure can have a square planar arrangement of the halogen ligands and two pyridine units (N−N−X−X; Figure 2f). The alternative structure can have one halogen and three pyridine units coordinated to the gold centre in the plane (N−N−N−X) and the remaining halogen above the plane. According to DFT calculations, the latter structure lies 48 kJ mol^−1^ higher in energy for [Au^III^(terpy)(Cl)_2_]^+^ and could not have been localized for [Au^III^(terpy)(Br)_2_]^+^ (see Figure S94). Hence, we conclude that the recombination of [Au^III^(terpy)(X)]^2+^ and X^−^ during the transfer to the gas phase proceeds via a pyridine‐to‐X^−^ replacement in the square planar arrangement.

The analysis of the fragmentation energetics shows that the binding energies of the halogens in the [Au^III^(terpy)(X)_2_]^+^ complexes are more than 60 kJ mol^−1^ smaller than in [Au^III^(bipy)(X)_2_]^+^. The reason probably stems from the assistance of one of the pyridine units in the elimination of the halogen radical (Figure 2f). The binding energies of the halogen atoms in the gold(II) complexes [Au^II^(terpy)(X)]^+^ remain very close to those in [Au^II^(bipy)(X)]^+^.

Table 2 shows a comparison of the experimental *BDE*s and the computed values (B3LYP−D3BJ/6‐311+G**, SDD/SDD ECP on Au and 6–311G** on I). The theoretical calculations overestimate the bond dissociation energies. In the exploratory calculations, we have found out that the overestimation is even larger if we use larger basis sets and if we (correctly) include the spin‐orbit coupling and nonscalar relativity (employing X2C method). We will investigate the origin of this peculiar effect in a large benchmarking study of the theoretical methods in future. For the purpose of this study, we will evaluate the relative trends (1st vs. 2nd dissociation; Cl/Br/I ligands) that are reasonably reproduced irrespective of various methodological ingredients.

The theoretical results show, in agreement with the experiment, that the gold(II) complexes are better stabilized with heavier halogen ligands and that the tridentate ligand stabilizes the complex better than the bidentate ligand. The formation of the gold(II) complexes from [Au^III^(terpy)(X)_2_]^+^ is about 60 kJ mol^−1^ less energy‐demanding than that from [Au^III^(bipy)(X)_2_]^+^. In theoretical calculations, this value is even higher and reaches 80–90 kJ mol^−1^. The discrepancy is caused by the fact that we measure Δ*E*_a_ in the experiment, whereas we calculate Δ*H* in the theory (Figure 2f). Typically, the simple bond cleavages are quasi‐barrierless endothermic processes and therefore the measured activation energy corresponds to the reaction enthalpy of the bond cleavage process, that is, to the BDE. In the case of [Au^III^(terpy)(X)_2_]^+^, the starting complex has a pseudo square planar geometry around the gold centre formed by two pyridine units and the two halogen atoms (see the initial structure in Figure 2f). The third pyridine unit interacts only weakly with the gold ion. The X elimination proceeds via a transition structure in which the initially loosely bound pyridine unit coordinates to the gold centre. The measured activation energy thus corresponds to the barrier height of this process.

##### Electrochemistry experiments

The experiments determining bond dissociation energies of the gaseous complexes show that the window of the stability of the gold(II) complexes is rather narrow. It is sufficient to generate these complexes in the gas phase, but it remains a question, whether these complexes could be also studied in solution. In order to test this possibility, we have prepared the [Au(bipy)X_2_]PF_6_ and [Au(terpy)X](PF_6_)_2_ complexes (see the Experimental Section and Supporting Information) and performed cyclic voltammetry (CV) experiments in dichloromethane (DCM) and dimethylformamide (DMF) solutions. We have selected DCM, because it is non‐polar non‐coordinating solvent and thus the conditions are closest to the gas phase experiments (Figures S79 and S81). Unfortunately, the terpy complexes were not soluble in DCM and therefore the comparison of all complexes was done in DMF.

**Figure 3:**
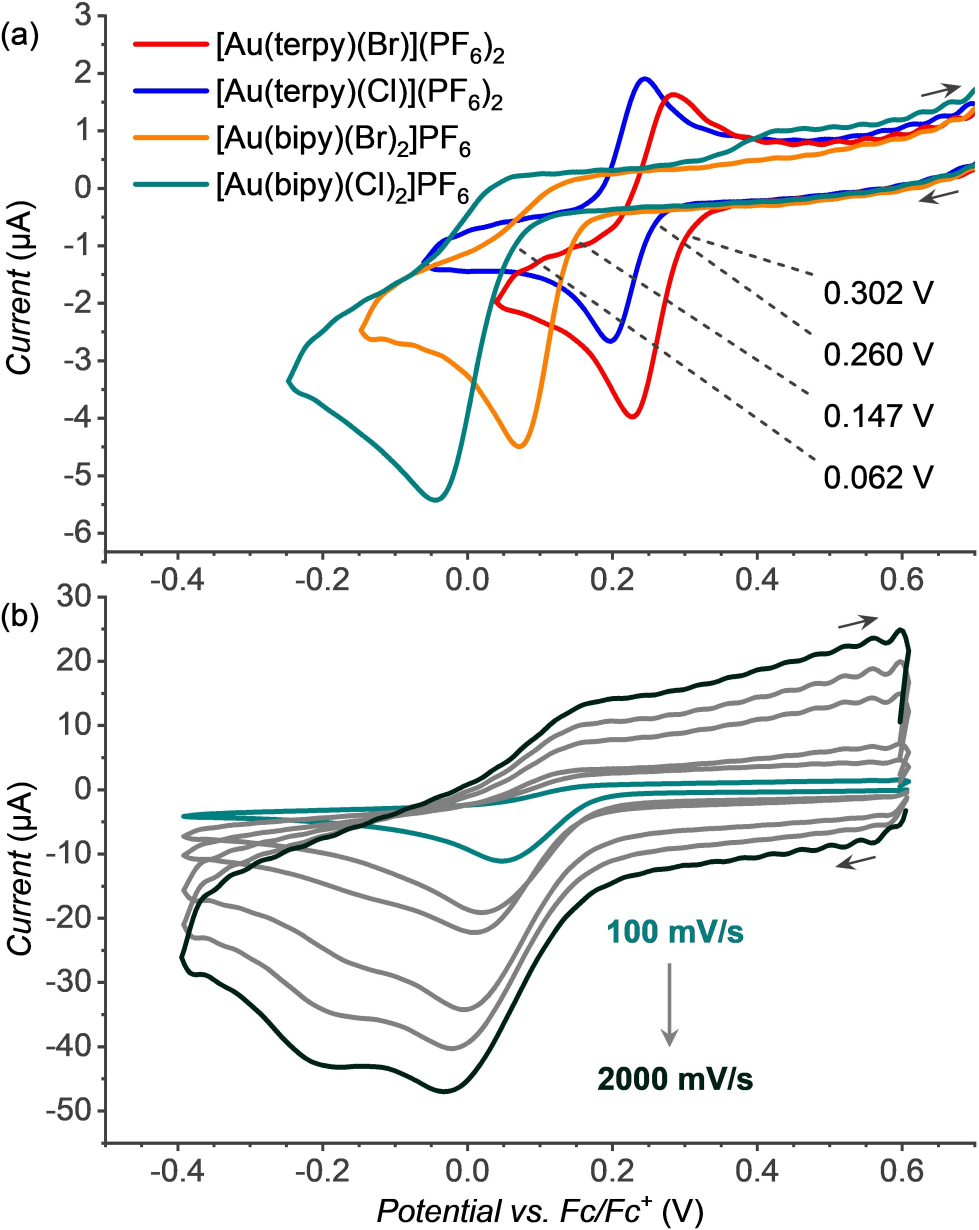
a) Cyclic voltammogram of [Au(bipy)(X)_2_]PF_6_ and [Au(terpy)(X)](PF_6_)_2_ (X=Cl or Br) in dimethylformamide (0.25 mM) in the range of the Au^III^→Au^I^ reduction potential (scan rate 100 mV s^−1^). b) Cyclic voltammogram of [Au(bipy)(Cl)_2_]PF_6_ in DCM (0.5 mM) at various scan rates (100, 300, 500, 1000, 1500 and 2000 mV s^−1^).

Figure 3 shows a comparison of the reduction waves of the [Au(bipy)(X)_2_]PF_6_ and [Au(terpy)(X)](PF_6_)_2_ (X=Cl or Br) complexes (X=Cl or Br) in DMF under the same conditions. We show the range of the CVs with the reduction waves corresponding to the reduction of gold(III) to gold(I) complexes. This 2e‐reduction process has been studied and discussed in detail for analogous complexes[^33^, ^53^, ^54^] with the bipy and terpy type ligands previously. The whole range of the CV experiments with the assignment of the Au^III^→Au^I^ and Au^I^→Au^0^ transitions is in Figures S78, S80, and S82–S88).

The comparison of the Au^III^→Au^I^ reductions waves for the studied complexes shows the trend of the shifts to more positive potentials in the order [Au(terpy)(Br)]^2+^>[Au(terpy)(Cl)]^2+^ and [Au(bipy)(Br)_2_]^+^>[Au(bipy)(Cl)_2_]^+^. The reduction of the complexes with the terpy ligand is reversible, whereas the reduction of the complexes with the bipy ligand is irreversible. We have further tested, whether we could separate the 2e‐reduction wave to the two one‐electron reduction steps by increasing the CV scanning rate.[^31^, ^55^] The only successful separation was achieved for the [Au(bipy)(Cl)_2_]^+^ complex (in DCM–Figure 3b – and in DMF‐Figure S85). With the increasing scanning rate, the reduction became partially reversible. This result suggests that the irreversibility is associated with the decomposition of the complex that is fast. We did not succeed in separation of the reduction steps for the bromide complexes which suggest that they decompose even faster than the chloride complexes.

##### Vibrational photodissociation spectra

Next, we investigated the spectroscopic properties in the infrared region for the [Au^II^(bipy)(X)]^+^ complexes (X=Cl and Br) in the gas phase and compared them with the analogous complexes of copper(II). While 2,2’‐bipyridine is usually not acting as a redox‐active ligand,^[56]^ it can be redox active in some complexes.[^57^, ^58^] In addition, some metals can activate a C−H bond of 2,2’‐bipyridine in a roll‐over mechanism.[^59^, ^60^] We have assumed that if the C−H activation reactivity or the redox reactivity would be important for the gold(II) complexes, then we should detect distinct signatures by vibrational and electronic spectroscopy of the mass‐selected complexes.[^61^, ^62^, ^63^, ^64^] For an easier interpretation, we compared all spectral features with those of analogous copper complexes.

**Figure 4:**
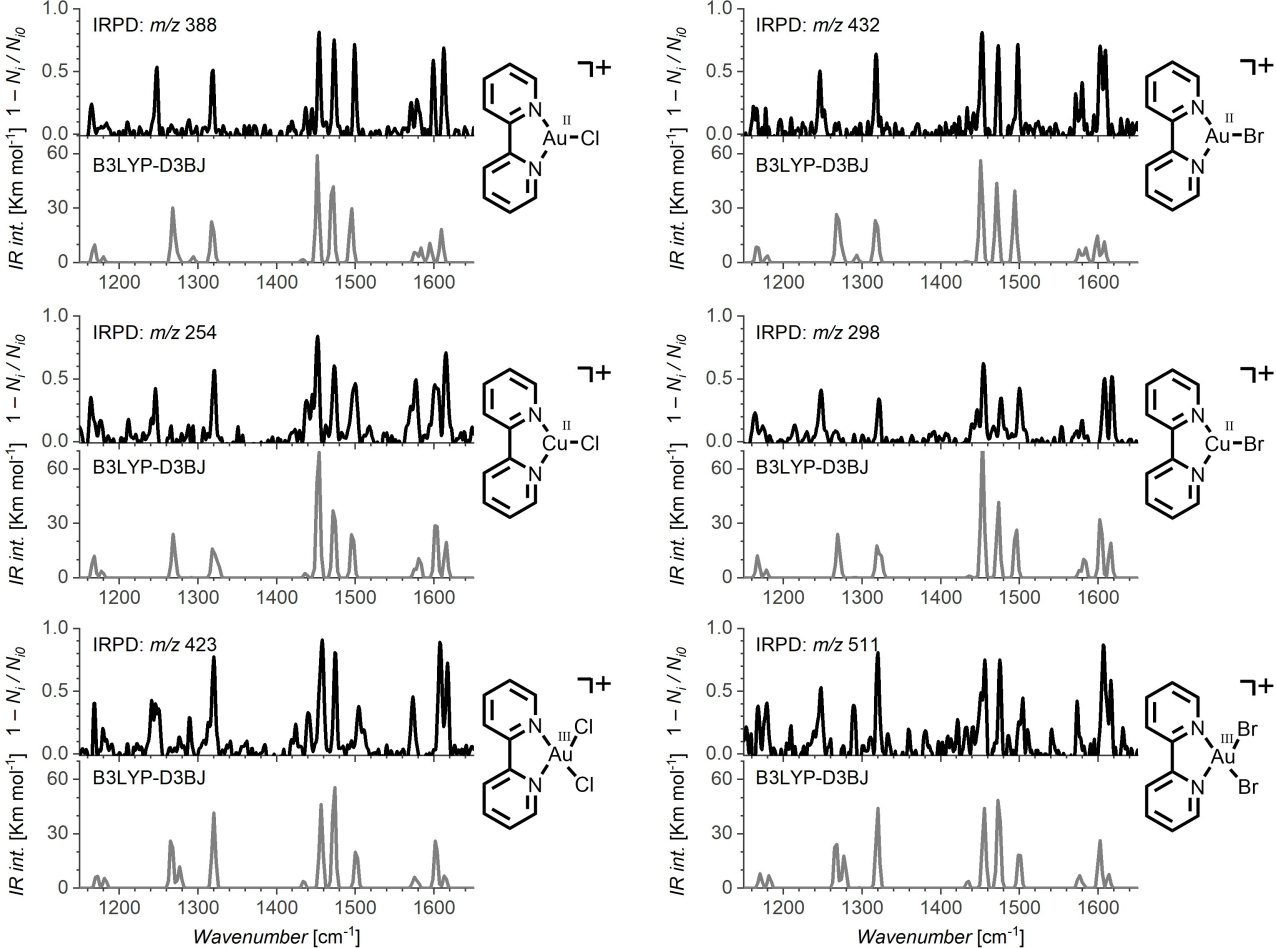
IR photodissociation spectra (measured by helium tagging photodissociation spectroscopy) of Au^III^, Au^II^ and Cu^II^ complexes (black lines) and comparison with their theoretically predicted IR spectra (grey lines, calculated by B3LYP−D3BJ/6‐311+G**(SDD on Au/Cu/Br), scaling factor: 0.98). See also Figures S89–S98.

We measured IR spectra by helium tagging photodissociation method.^[65]^ In the range of our OPO IR light source, the spectra show mostly only the vibrations of the bipy ligand (Figure 4). The comparison of gold and copper (M=Au or Cu) analogues of [M^II^(bipy)(Cl)]^+^ and [M^II^(bipy)(Br)]^+^ reveals that both metals interact with the ligand in the same way (compare the top IRPD spectra in both columns in Figure 4). The spectra do not reveal any significant differences. We also measured IR spectra of the gold(III) precursors (bottom IRPD spectra in Figure 4). The gold(III) complexes show a very similar fingerprint of the bipy ligands as the respective gold(II) complexes. The comparison of the experimental spectra with the IR spectra predicted by DFT (B3LYP−D3BJ^[66]^ – the most frequently used functional in ion spectroscopy[^67^, ^68^, ^69^] – in grey in Figure 4) shows a very good agreement. The agreement suggests that the DFT method describes well the interaction between gold(II) and the ligand as well as the overall geometry of the complex. The assignment of the vibrational bands is shown in Figure S91.

We also measured the IRPD spectra of [Au(terpy)(Cl)_2_]^+^ and [Au(terpy)(Cl)]^2+^ (Figures S94 and S96). They are very similar to the spectra of the bipy complexes. The spectra show the vibrations of the terpy ligand that are similar to those of the bipy ligand. The experimental spectrum of [Au(terpy)(Cl)_2_]^+^ better agrees with the theoretical spectrum of the more stable isomer of [Au(terpy)(Cl)_2_]^+^ with the N−N−Cl−Cl planar arrangement of the ligands, but the IR spectrum of the alternative high‐energy isomer with the N−N−N−Cl arrangement of the ligands is rather similar (Figure S94).

## Electronic photodissociation spectra

**Figure 5:**
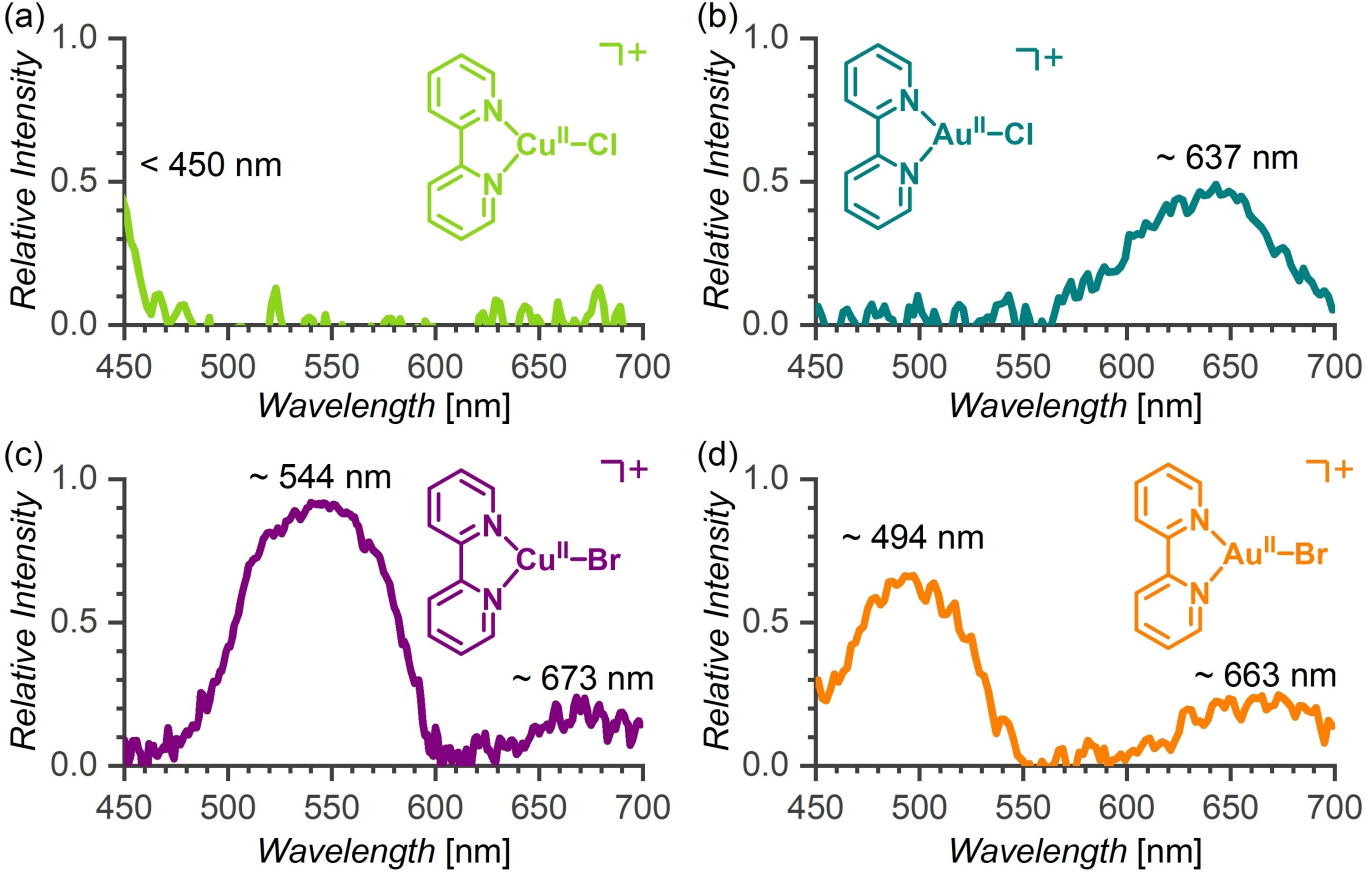
Helium‐tagging photodissociation spectra in the visible range (visPD) of Cu^II^ and Au^II^ complexes at 3 K.

Next, we have characterized the newly generated gold(II) complexes [Au^II^(bipy)(X)]^+^ (X=Cl or Br) by measuring their absorption spectra in the visible range using the helium tagging photodissociation method. For the comparison, we measured also the spectra of the stable copper(II) analogues [Cu^II^(bipy)(Cl)]^+^ and [Cu^II^(bipy)(Br)]^+^. The copper chlorido complex has an absorption band below 450 nm. We could detect only the onset of this band, because of the working range of our laser (Figure 5a). The absorption maximum for [Cu^II^(bipy)(Br)]^+^ is red shifted to 544 nm (Figure 5c). For the gold complexes, we detected a band at 637 nm for [Au^II^(bipy)(Cl)]^+^ (Figure 5b) and two bands at 494 nm and 663 nm for [Au^II^(bipy)(Br)]^+^ (Figure 5d). In order to explain the spectra and get a deeper insight into the properties of gold(II) complexes, we performed quantum chemical calculations.

## Computed electronic spectra

To provide an interpretation and computational support of the experimental absorption spectra (Figure 5), we employed the CAM‐B3LYP/aug‐cc‐pVTZ/Au(PP) density functional theory method. The equilibrium geometries were obtained at the PBE0/def2‐TZVPP/PP(Au)[^70^, ^71^] level. This combination provided a good agreement between the computed and experimental data and afforded their straightforward interpretation, though it partly relies on error cancellation, see the Supporting Information. A comparison of the DFT functionals, basis set requirements, subtle structural effects on the computed spectra and the spin‐orbit effects are given in the Supporting Information, including the full set of the calibration data, Tables S2−S5.

**Figure 6:**
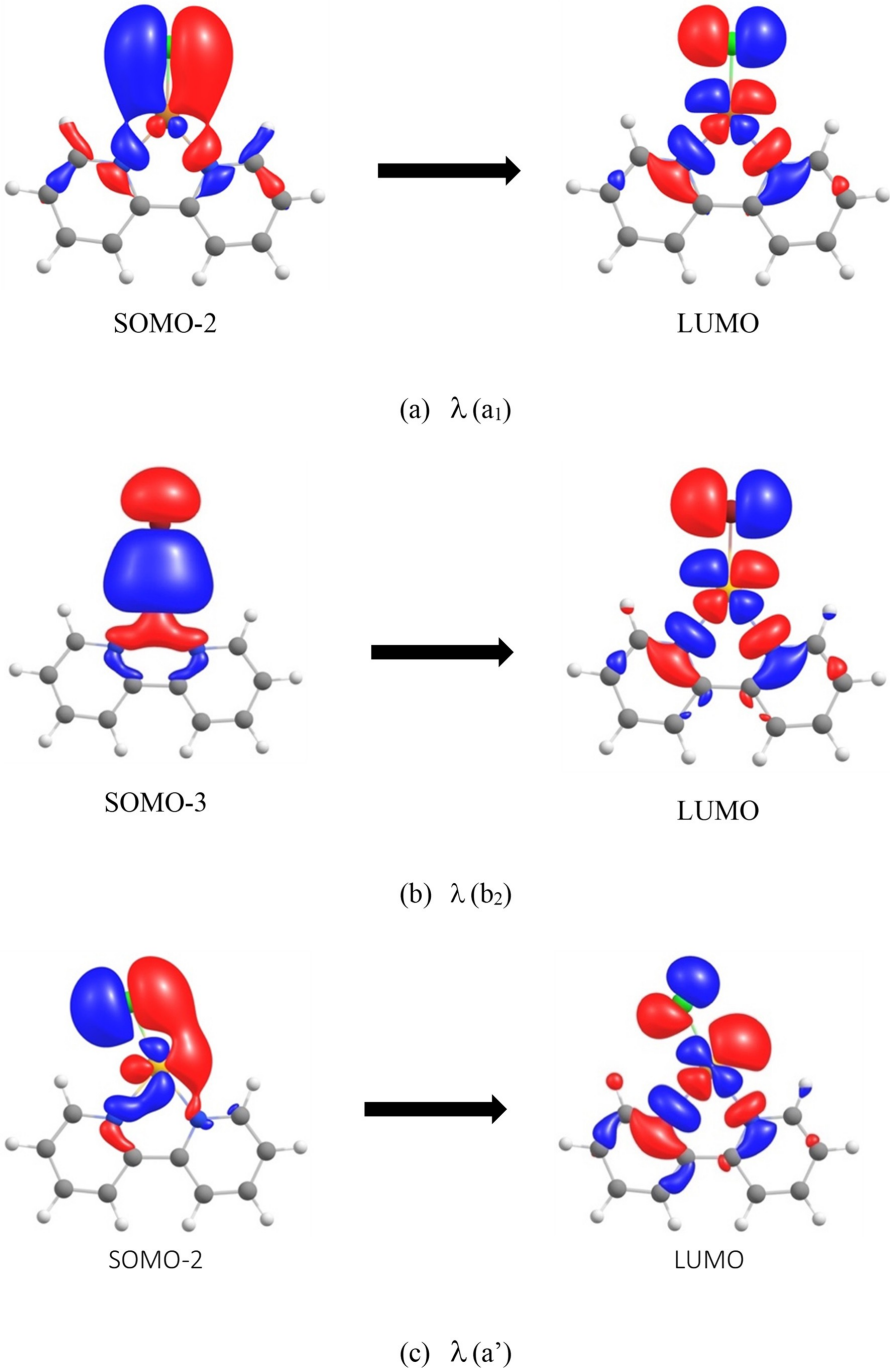
The MO origin of studied excitations. a) *a*_1_ excitation from the *b*_2_ LP(X) SOMO‐2 to *b*_2_ π(M−X)* LUMO; in the *C*_2v_ geometry. b) *b*_2_ excitation from *a*_1_ σ(M−X) SOMO‐3 to *b*_2_ π(M−X)* LUMO; in the *C*_2v_ geometry. c) *a’* excitation from the *a’’* LP(X) SOMO‐2 to *a’’* π(M−X)* LUMO in the *C*_s_ geometry.

The calculations clearly reveal the origin of two transitions responsible for the spectral features that are common to all four studied complexes. The transitions are illustrated in Figure 6. It can be seen that the experimental band near 650 nm (Figure 5) arises from LP(X)>π(M−X)* *a*_1_ (LP: lone pair, *a’* in the *C*_s_ symmetry, see below) excitation (Figure 6a) whereas the experimental band near 500 nm (Figure 5) arises from the σ(M−X)>π(M−X)* *b*_2_ (*a’’* in *C*_s_) excitation (Figure 6b). These common features are calculated at all tested levels, DFT and ab initio (Tables S2–S5).

Comparison of theoretical and experimental data shown in Table 3 reveals that for X=Br, that is, for the [Cu(bipy)(Br)]^+^ and [Au(bipy)(Br)]^+^ complexes, both transitions are experimentally observed and the calculations are in a good agreement with the experimental values (to within 2–20 nm). For [Cu(bipy)(Cl)]^+^, the predicted *λ*_calc_ (*b*_2_)=455 nm seems to correspond to the experimental band appearing at the end of the short wavelength of the experimental spectra (∼450 nm) whereas the predicted *λ*_calc_ (*a*_1_) at 684 nm seems to be hidden in noise on the other end of the experimental spectra (Figure 5a). For [Au(bipy)(Cl)]^+^, the predicted *λ*_calc_ (*b*_2_)=416 nm in the *C*_2v_ geometry and *λ*_calc_ (*a’‘*)=433 nm in the *C*_s_ minimum (see Discussion below) is clearly outside the experimental window (*λ*_min_∼450 nm). This explains the missing *b*_2_ band in the spectrum of [Au(bipy)(Cl)]^+^ (Figure 5b).

**Table 3:** Comparison of theoretical and experimental absorption resonances for [M(bipy)(X)]^+^ M=Cu, Au; X=Cl, Br systems. In nm. Calculated oscillator strengths are in parentheses. CAM‐B3LYP/aug‐cc‐pVTZ/Au(PP)//PBE0/def2TZVPP/Au(PP) level.

Molecule	Sym.	*λ*_calc_ (*b*_2_)	*λ*_exp_(*b*_2_)	*λ*_calc_ (*a*_1_)	*λ*_exp_(a_1_)
[Cu(bipy)(Cl)]^+^	*C*_2v_	455(0.12)	<450	684(4×10^−4^)	–
[Au(bipy)(Cl)]^+^	*C*_2v_	416(0.17)	–	639(1×10^−4^)	637
[Cu(bipy)(Br)]^+^	*C*_2v_	545(0.12)	544	694(4×10^−4^)	673
[Au(bipy)(Br)]^+^	*C*_2v_	495(0.17)	494	664(1×10^−4^)	663
[Au(bipy)(Cl)]^+^	*C*_s_	433(0.01) ^[a]^	–	665(0.01) ^[a]^	637

[a] C_s_‐symmetry. *λ*_calc_ (a’‘) and *λ*_calc_ (a’)

**Figure 7:**
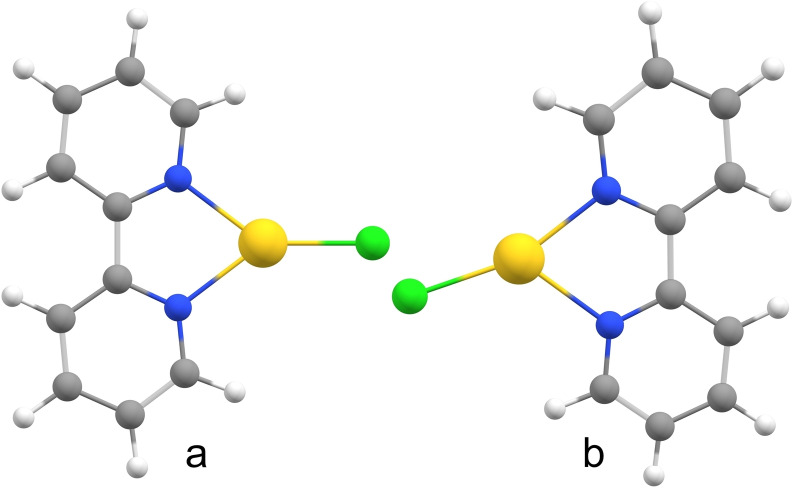
a) C_2v_ and b) *C*_s_ structures of the [Au(bipy)Cl]^+^ systems.

As for the computed intensities of the two peaks, the computed value of the oscillator strength for the *a*_1_ excitation (*f* ≈1–4×10^−4^, Figure 6b, Table 3) at the *C*_2v_ minima is rather low. We assumed that it was caused by neglecting the vibronic coupling, because the in‐plane bending mode (Figure 7) of the halogen atom in the [M(bipy)(X)]^+^ complex is associated with a very low vibrational frequency of a few tens of cm^−1^. In energy terms, this corresponds to a computed deformation energy of 2–4 kJ mol^−1^ (see below).

Interestingly, in the case of [Au(bipy)(Cl)]^+^ complex, the equilibrium geometry at the PBE0/de2TZVPP/Au(PP) level corresponds to the *C_s_* minimum (Figure 7a); whereas the *C*_2v_ structure lies ∼2 kJ mol^−1^ above (Figure 7b). In fact, the latter (C_2v_) is a transition state with an imaginary mode of *i*30 cm^−1^, whereas the lowest vibrational mode in the *C*_s_ minimum (30 cm^−1^) corresponds to soft in‐plane ligand‐gold‐chlorine bending. More importantly, for the *C*_s_ minimum of [Au(bipy)(Cl)]^+^ the computations predict ∼100 times higher oscillator strength for the *λ*_calc_(*a’*) excitation in comparison with the *λ*_calc_(*a*_1_) in the *C*_2v_ geometry (*f*=0.0103 vs. 0.0001, *c.f*. Table 3). The predicted wavelength of the *λ*_calc_(*a*’) transition raises from 639 to 665 nm (1.94 to 1.86 eV), which is admittedly away from the experimental value of 637 nm (1.95 eV).

Similar arguments can be applied for [Au(bipy)(Br)]^+^. The equilibrium geometry of [Au(bipy)(Br)]^+^–at all tested levels–corresponds to a *C*_2v_ minimum, for which the calculated oscillator strength of the *a*_1_ transition is also very low, *f* ≈10^−4^ (Table 3). However, a relaxed scan of the N1‐Au−Br tilt angle reveals that the energy of the system changes only very little (<2 kJ mol^−1^ for 20°), while the intensity of the *a*’ transition strongly raises, by ∼100 times for the same (20 degrees) tilt, c.f. Table S6. The *λ*_calc_ (*a’*) calculated then raises to 754 nm (1.64 eV), relatively far from the experimental value of 663 nm (1.87 eV). Inclusion of spin‐orbit coupling in calculations may decrease the predicted value by few tens of nm as shown in Table S2. All in all, we qualitatively demonstrated the sensitivity of the computed intensities on the small structural changes of the studied complexes.

It can be thus concluded that the ratio between the computed intensities of the two peaks is between 1 : 10 and 1 : 100. This still seems to be somewhat in disagreement with the experimental spectrum (Figure 5c) for which the ratio 1 : 3 can be inferred from the visual inspection of the figure. However, the band intensities in the visPD spectra depend on the laser power that varies with the wavelength (Figure S99). In addition, the spectra were measured with large attenuations (close to the saturation regime) with the aim to detect also less intense bands. In a linear regime, the ratio between the bands would be larger, making the agreement between experimental and theoretical results qualitatively correct.

As mentioned above, the *a*_1_ band is either missing or hidden in the noise in the spectra of [Cu(bipy)(Cl)]^+^ system, Figure 5a. Calculations of *C*_s_‐symmetric [Cu(bipy)(Cl)]^+^ with Cl artificially bent by 20 degrees from the *C*_2v_ minimum give *λ*_calc_ (*a*_1_)=679 nm with oscillator strength ∼0.003 that is only about ten times higher than in the *C*_2v_ minimum. Apparently, the vibronic coupling is much weaker in [Cu(bipy)(Cl)]^+^ and the corresponding band is not well observed in the spectrum.

### Oxidation state and electronic configuration of the metal

To check the unusual oxidation state II of the gold ion in the studied complexes, quantum theory of atoms in molecules (QTAIM) analysis^[72]^ of the electronic structure has been carried out employing the localization index, LI_A_ as the central property. The oxidation state of an electropositive element in contact with elements that are more electronegative can be defined as the difference between its atomic number and its LI_A_.^[73]^ This quantity is consistent with the IUPAC definition of the oxidation state that is atomic charge after ‘‘ionic approximation” of the heteronuclear bonds. The QTAIM analysis of [M(bipy)(X)]^+^ (M=Cu, Au; X=Cl, Br, I) complexes in Table 4 shows that while the atomic charges of M vary from 0.4 for [Au(bipy)(I)]^+^ to 0.9 for [Cu(bipy)(Cl)]^+^, the LI_M_ index remains rather constant among all species, confirming the II oxidation state of the metal. The differences between the LI_M_ and atomic numbers for copper and gold complexes are similar to each other, which is consistent with their similar oxidation numbers.

**Table 4:** The atomic charges, Q, on metal, halogen, and nitrogen atoms, localization index of the metals (LI) and delocalization index (DI) between metal and halogen, and metal and nitrogen atoms. Spin densities at metal, halogen and nitrogen atoms; atomic properties of the nitrogen atoms in the asymmetric structures are reported for N1 and N2 as marked in Figure 7, respectively. Calculated at TPSS/def2TZVP level.

Molecules	Q_M_	Q_X_	Q_N_	LI_M_	OS(rounded)	DI_MX_	DI_MN_	SD_M_	SD_X_	SD_N_
[Au(bipy)(Cl)]^+^	0.65	−0.28	−1.10	76.89	+2	1.33	0.66	0.43	0.27	0.13
[Au(bipy)(Cl)]^+[a]^	0.72	−0.36	−1.20/−1.19^**[a]**^	76.82	+2	1.26	0.71/0.69^**[a]**^	0.53	0.17	0.05/0.22^**[a]**^
[Au(bipy)(Br)]^+^	0.56	−0.16	−1.10	76.97	+2	1.40	0.65	0.40	0.32	0.12
[Au(bipy)(I)]^+^	0.41	0.02	−1.10	77.10	+2	1.51	0.62	0.35	0.41	0.10
[Cu(bipy)(Cl)]^+^	0.94	−0.42	−1.16	26.80	+2	1.13	0.59	0.50	0.28	0.10
[Cu(bipy)(Br)]^+^	0.87	−0.34	−1.15	26.86	+2	1.19	0.58	0.47	0.33	0.09
[Cu(bipy)(I)]^+^	0.75	−0.18	−1.15	26.94	+2	1.29	0.56	0.42	0.42	0.07

^[a]^ *C*_s_ minimum.

The bonding between Au^II^ and the other atoms in the molecule can be described by the delocalization index, DI, that expresses the level of electron sharing, that is, covalency, between two atoms. The results in Table 4 point to the covalent M−X bonds with DI_M‐X_ values varying between 1.1 ([Cu(bipy)(Br)]^+^) and 1.5 ([Au(bipy)(Cl)]^+^). The DI_M‐X_ higher than 1 denotes multiple bonding, see NBO analysis below. Dative bonds are predicted between M and nitrogen atoms, with DI_M‐N_ about 0.5–0.7 for each nitrogen in Table 4. It is worth noting that as the covalent character of M−X in both copper and gold complexes increases, spin density on the metal centres decreases. This is compensated by an increase of the spin density at the halide atoms. Visualizing the spin density in 2D and 3D plots, Figures S104–S106, shows that the unpaired electron is placed in the d orbitals of the gold atom with some contribution of the X and nitrogen atoms. The calculated spin densities are listed in Table 4.

**Figure 8:**
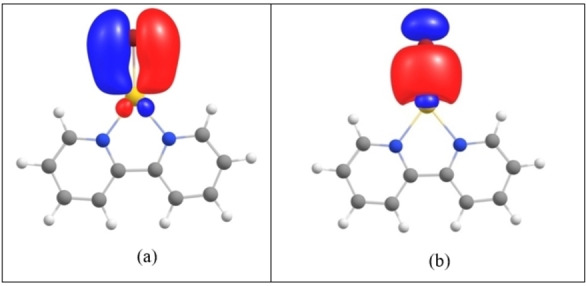
M−X bonding NBOs found in the [Au(bipy)(Br)]^+^ system. a) Single occupied alpha NBO corresponding to one‐electron LP_π_(X)>d${}_{{}_{\pi *}}$ (M) interaction and b) NBO that corresponds to the σ(M−X) bond. Here, in fact, two NBOs, one alpha and one beta singly occupied NBOs share similar spatial shape, so only one is shown.

The multiple bonding character of M−X bonds (DI_M‐X_ >1 in Table 4) is reflected in the NBO analysis of ([Au(bipy)(Br)]^+^) that finds three one‐electron bonding NBOs between Au and X. Two of these one‐electron bonds share similar spatial shape but one hosts an alpha‐ and the other hosts a beta electron (Figure 8b) which basically corresponds to a two‐electron σ(M−X) bond. The third M−X bonding NBO corresponds to one‐electron LPπ (X) donation to d_π*_(M), Figure 8a. The Au−N interactions are also observed in all systems. The NBO procedure finds LP(N) NBOs that strongly interact with the M−X antibonding NBOs.

## Discussion

The isolation of ions in the gas phase allowed us to study the properties of gold(II) complexes with [Au^II^(L)(X)]^+^ speciation (L=bidentate or tridentate ligand, X=halogen) in detail. The gold(II) complexes can be prepared by reduction of gold(III) precursors. The challenge in this preparation is a possibility of an easy direct reduction of gold(III) complexes to gold(I) complexes. We have explored the possibility to generate the gold(II) complexes by sequential eliminations of halogen radicals from the [Au^III^(L)(X)_2_]^+^ precursors in the gas phase for various L and X combinations. The experiments clearly showed that the ligands L that stabilize gold(I) complexes do not favour the formation of the desired gold(II) complexes. This includes all monodentate ligands that easily support favourable linear coordination of gold(I) complexes and ligands with ‘‘soft” donor atoms (especially P‐based). The most favourable ligands for the formation of gold(II) halido complexes were N,N’‐bidentate and N,N’,N’’‐tridentate ligands.

**Figure 9:**
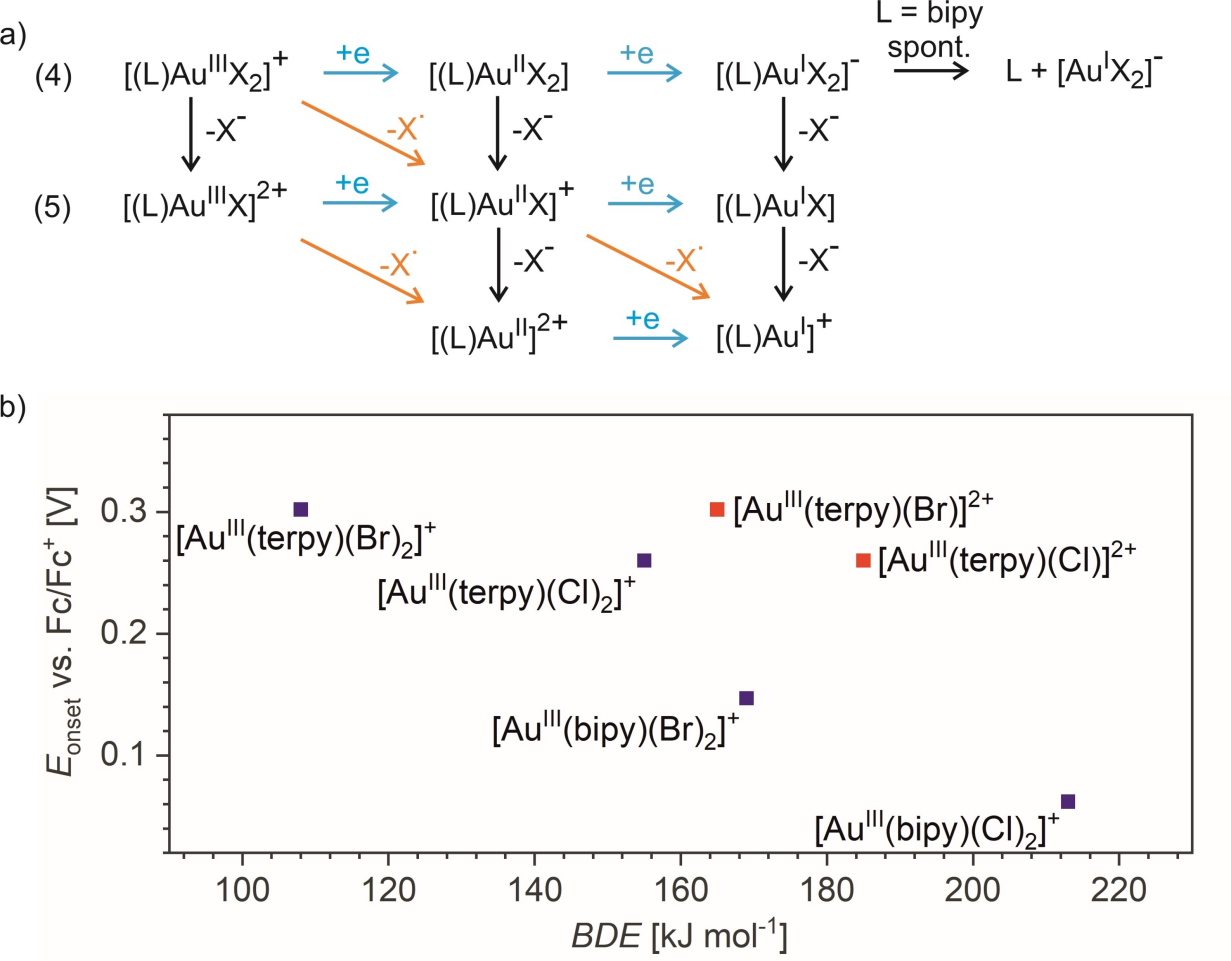
a) The possible processes in solution (one‐electron reductions are indicated by blue arrows, ligand dissociations are indicated by black arrows) and the processes studied by collision‐induced dissociation experiments (radical cleavages indicated by orange arrows). b) Correlation between *E*_onset_ of the Au^III^→Au^I^ redox potentials and the BDEs of the [Au^III^(L)(X)_2_]^+^ complexes (blue symbols) and [Au^III^(terpy)(X)]^2+^ (red symbols; L=terpy or bipy, X=Br or Cl).

Knowing the ligands that can stabilize gold(II) halido complexes, we have attempted to prepare the respective gold(II) complexes also in solution by electrochemical reduction. Unfortunately, the electrochemical reaction led always to the two‐electron reduction and the formation of gold(I) complexes. Nevertheless, we have observed the trend in the onset potential for the reduction of the gold(III) complexes that correlates well with the gas‐phase halogen bond dissociation energies from the respective [Au^III^(L)(X)_2_]^+^ ions (Figure 9b). For the tridentate ligand terpy, the reduction is reversible and the ions are speciated as [Au^III^(terpy)(X)]^2+^ in solution (Figures S76 and S77).[^74^, ^75^, ^76^] Accordingly, the dications are also the dominant complexes detected by electrospray ionization (ESI) mass spectrometry (Figures S22 and S29). The [Au^III^(terpy)(X)]^2+^ complexes can accept 2 electrons without forming an unstable species (Reaction 5 in Figure 9a). In contrast, the bidentate ligand will support [Au^III^(bipy)(X)_2_]^+^ speciation. Electrochemical reduction of these complexes was always irreversible. Likely, the [Au^III^(bipy)(X)_2_]^+^ complexes accept two electrons and then spontaneously dissociate to form linear [Au(X)_2_]^−^ and the free ligand. Alternatively, the complexes could dimerize.[^32^, ^33^, ^77^] Interestingly, a correlation between the BDEs of the gold‐halogen bonds in [Au^III^(L)(X)_2_]^+^ and the reduction onset potentials (blue points in Figure 9) suggests that the energy of the gas‐phase homolytic M−X bond cleavage to a certain degree predict the redox potential of the metal M. The fact that we see a better correlation with the BDE values measured for [Au^III^(terpy)(X)_2_]^+^ than with those for the solution‐relevant [Au^III^(terpy)(X)]^2+^ complexes is most probably because of the charge. The double charge increases the BDEs in the gas‐phase. Hence, the additional chlorido ligands in [Au^III^(terpy)(X)_2_]^+^ compensate this effect and allow the rough correlation even in a family of different ligands.

The possibility of generating the gold(II) halido complexes in the gas phase allowed us to characterize their spectroscopic properties. Using infrared photodissociation spectroscopy, we could show that gold(II) has a very similar electronic interaction with the auxiliary ligands as copper(II). The IR spectra showing the vibrations of bipy ligand do not suggest any unpaired electron delocalization at the ligand. The gold(II) complexes absorb also in the visible range. In comparison to the analogous copper(II) complexes, their absorption maxima are slightly blue shifted. The spectra suggest the orange‐red colour for [Au^II^(bipy)(Br)]^+^ and cyan for [Au^II^(bipy)(Cl)]^+^. The theoretical analysis of the spectra showed that the excitations are metal‐centred and also demonstrated how subtle structural variations and vibronic coupling dramatically change the intensities of peaks and also shift non‐negligibly the positions of the absorption peaks.

QTAIM calculations confirm that both gold and copper are in oxidation state M^II^ in calculated [(bipy)M^II^X]^+^ systems, with the calculated M−X bond order between 1 and 1.5 (multiple bonding) and with the M−N bond orders near 0.7 (dative bonding). The NBO analysis reveals a strong interaction of the in‐plane LP(X) with the half‐empty d orbital at the gold(II) centre.

## Conclusions

We have shown that gold(II) complexes with bidentate and tridentate ligands can be generated in the gas phase by the elimination of halogen radicals from the gold(III) halogen precursors. We have further explored the spectroscopic and electronic properties of the [Au^II^(bipy)(X)]^+^ complexes in detail, because these complexes are not fully coordinatively saturated and thus offer the possibility to study the reactivity of gold(II) complexes in the future. The results show that the interaction of gold(II) with the ancillary ligand bipy is analogous to that of copper(II). Moreover, the electronic spectra of the gold(II) complexes are similar to those of the analogous copper(II) complexes. The absorption maxima in the spectra are blue‐shifted for the gold complexes, thus suggesting that [Au^II^(bipy)(Br)]^+^ should be orange‐red and [Au^II^(bipy)(Cl)]^+^ should be cyan in colour.

## Experimental Section

**Mass spectrometry**: Mass spectrometric (MS) experiments were carried out using the Thermo Scientific LTQ XL linear ion trap mass spectrometer with an ESI source.^[78]^ Energy‐resolved collision‐induced dissociation spectra were measured with an LCQ Deca XP ion trap mass spectrometer.^[79]^ In the CID experiments, BDE can be determined from the dependence of the relative cross‐section of the given dissociation on the collision energy.^[80]^ The linear extrapolation of the rise of the sigmoid fit of the cross‐section energy dependence gives the appearance energy of the given dissociation channel (see Figure 1 and the data in the Supporting Information). The appearance energies are related to the bond dissociation energies associated with the given fragmentations. The collision energies in the LCQ instruments can be calibrated based on the measurements of the dissociation energies of a series of ‘‘thermometer” alkylpyridinium ions (Figure S1).[^81^, ^82^, ^83^] The ions of interest were generated by ESI from dichloromethane/acetonitrile (DCM/ACN) solution of the respective 1 mM ligand and 1 mM AuX_3_ stock solutions. The stock solutions were mixed in a 1 : 1 ratio and diluted to achieve the final 100 mM concentration. The electrospray voltage was 4–5 kV and the capillary was heated to 150–220 °C. The sheath gas, flow rate, and voltages of capillary and lenses were optimized in order to obtain the maximum ESI‐MS signals of the required ions. CID experiments were performed for mass‐selected ions with a trapping parameter *q*_z_=0.25 and the excitation period of 30 ms.

**Infrared and UV‐vis photodissociation spectroscopy**: The IR and visible spectra of mass‐selected ions were measured with a helium tagging photodissociation method using the ISORI instrument.^[65]^ The ions were generated by the ESI, mass selected by a quadrupole, and transferred to a wire quadrupole trap operated at 3 K. The ions were trapped and cooled down using the helium buffer gas. The relaxed ions attached a helium atom. The helium complexes then served for the spectroscopic experiments. The complexes were irradiated with the OPO/OPA system from LaserVision pumped by Nd:YAG laser Surelite EX from Continuum (tuning range 700–4700 cm^−1^, FWHM ∼ 1.5 cm^−1^, 10 ns pulse length) or with supercontinuum laser NKT Photonics SuperK Extreme (430–700 nm range using acusto‐optic tuneable filter SuperK Select).^[84]^ The spectra are constructed as (1‐*N*_i_/*N*_i0_), where *N*_i_ and *N*_i0_ are numbers of helium complexes with and without laser irradiation.[^85^, ^86^]

**Electrochemistry**: The gold(III) complexes were prepared according to the literature.^[87]^ The NMR spectra of the solutions of the complexes in CD_3_CN on comparison with the literature indicate that the complexes are present as [Au(bipy)Cl_2_]^+^, [Au(bipy)Br_2_]^+^, [Au(terpy)Cl]^2+^ and [Au(terpy)Br]^2+^ (Figures S74–S77).[^74^, ^75^, ^76^, ^87^] We note that overtime these complexes especially the terpy based gold(III) complexes decomposed in solution as precipitation was observed. The speciation can depend on the solvent, but the results are consistent with the dominant speciation detected by NMR. Cyclic voltammetry (CV) experiments were performed using a Metrohm potentiostat (Autolab PGSTAT204) at room temperature in a solution of 0.25 or 0.5 mM of the respective gold complex (freshly prepared), 0.1 M of the supporting electrolyte (tetrabutylammonium hexafluorophosphate) either in DMF or in DCM. Glassy carbon working electrode, Pt counter electrode and a double junction nonaqueous Ag/AgCl reference electrode filled with 2 M LiCl in ethanol as the inner electrolyte was used. Prior to the measurements the electrolyte was deoxygenated by argon bubbling, and during the measurement electrolyte was kept under the argon atmosphere. Reference electrode was calibrated against ferrocene redox couple before measurements.

**DFT calculations**: All quantum chemical calculations were carried out using Gaussian 16,^[66]^ and Turbomole 7.5.1. programs.^[88]^ Density functional theory (DFT) has been mostly used throughout. A set of density functionals, including pure generalized‐gradient approximation (GGA),^[89]^ meta‐GGA and hybrids were employed: B3LYP, PBE, TPSS, and PBE0. Basis sets of triple‐zeta quality‐6‐311+G**, and def2‐TZVP(P) – used throughout are considered as sufficiently large to report the converged DFT values. For gold and iodine core electrons, Stuttgart−Dresden (SDD) pseudopotentials (ECP's) were used.[^90^, ^91^, ^92^, ^93^, ^94^, ^95^, ^96^] The electronic excitation spectra were mostly calculated by employing time‐dependent (TD‐) DFT theory as implemented in Gaussian 16. For comparison, the ab‐initio CC2 and ADC2 calculations were performed using the Turbomole 7.5.1 code. The spin−orbit SO‐ZORA excitations we obtained in the ADF 2021 program suite.[^97^, ^98^, ^99^, ^100^] The particular method used for computations is always denoted in the following by employing the standard notation: Method A/basis set A//method B/basis set B. This describes the level of theory at which the geometry was optimized (method B/basis set B) and the final single‐point energy (method A/basis set A) calculated. Zero‐point vibrational energy (and entropic and thermal contributions, if needed) were obtained employing the standard normal‐mode analysis.

**QTAIM and NBO analysis**: To investigate chemical bonding and to determine the oxidation states of metal in the studied complexes, quantum theory of atoms in molecules, QTAIM,^[101]^ was employed. All QTAIM computations were performed by the AIMAll suite of programs.^[102]^ Natural bond orbital analysis[^103^, ^104^, ^105^, ^106^, ^107^] used program NBO 7.0[^103^, ^108^] linked to the Gaussian 16 code.^[66]^

## Conflict of interest

The authors declare no conflict of interest.

1

## Supporting information

As a service to our authors and readers, this journal provides supporting information supplied by the authors. Such materials are peer reviewed and may be re‐organized for online delivery, but are not copy‐edited or typeset. Technical support issues arising from supporting information (other than missing files) should be addressed to the authors.

Supporting Information

## Data Availability

The data that support the findings of this study are available from the corresponding author upon reasonable request.
