# Supplementary material for: Cationic Gold(II) Complexes: Experimental and Theoretical Study
Source: Chemistry. 2022 Sep 1;28(60):e202201794. doi: 10.1002/chem.202201794 (PMC9805138; doi:10.1002/chem.202201794)
Supplement: Supplementary file 1 — Supporting Information [file CHEM-28-0-s001.pdf]

Chemistry–A European Journal

Supporting Information

Cationic Gold(II) Complexes: Experimental and Theoretical Study

Jaya Mehara, Adarsh Koovakattil Surendran, Teun van Wieringen, Deeksha Setia, Cina Foroutan-Nejad, Michal Straka,* Lubomír Rulíšek, and Jana Roithová*

Contents

Mass spectrometric studies: 3

ESI spectrum of various ligands from Table 1 with AuX_3 (Source spectrum, CIDs and(/or) energy resolved CIDs) 5

1) 2,2'-bipyridine with $AuCl_3$ 5
2) 2,2'-bipyridine with $AuBr_3$ 6
3) [(bipy)$AuCl_2$]PF_6 in acetone with excess of NaI 8
4) 2, 2’:6’, 2”-terpyridine with $AuCl_3$ 9
5) 2, 2’:6’, 2”-terpyridine with $AuBr_3$ 11
6) 1,10-Phenanthroline with $AuCl_3$ 14
7) 1,10-Phenanthroline with $AuBr_3$ 15
8) Pyridine with $AuCl_3$ 17
9) Pyridine with $AuBr_3$ 18
10) 1,1-Bis(diphenylphosphino)methane with $AuCl_3$ 19
11) 1,2-Bis(diphenylphosphino)ethane with $AuCl_3$ 19
12) 1,2-Bis(diphenylphosphino)ethane with $AuBr_3$ 20
13) 1,3-Bis(diphenylphosphino)propane with $AuCl_3$ 21
14) 1,3-Bis(diphenylphosphino)propane with $AuBr_3$ 22
15) 1,4-Bis(diphenylphosphino)butane with $AuCl_3$ 24
16) 1,4-Bis(diphenylphosphino)butane with $AuBr_3$ 25
17) Tetramethylethylenediamine with $AuCl_3$ 25
18) Tetramethylethylenediamine with $AuBr_3$ 26
19) Bipyridine with $CuCl_2$ 27
20) Bipyridine with $CuBr_2$ 27

Synthesis of gold(III) complexes 29

Electrochemistry experiments 32

IRPD and Vis spectra 38

Theoretical Details 45

Method calibration for electronic spectra 45

References 52

Mass spectrometric studies:

The experiments were performed on either Thermo Scientific LTQ XL linear trap or Finnigan LCQ Deca XP mass spectrometer equipped with an electrospray ionization (ESI) source.[1] General conditions were as follows: sheath gas 5-40 arbitrary unit, auxiliary gas 0-5 arbitrary unit, capillary temperature 150-220 ℃, spray voltage 2-5 kV, capillary voltage 0-50 V and tube lens 0-150 V.
Helium tagging photodissociation method was used to measure the IR spectra of the mass selected complexes on the ISORI instrument equipped with ESI source.[2] The ISORI instrument features a wire quadrupole trap operated at 3-5K, trapped ions are cooled down because of the helium buffer gas. These cooled ions then attach a helium atom, the helium complexes on irradiation with the IR laser undergo helium detachment and the IR spectra is constructed as $(1 - N_i/N_{i0})$, where N_i and N_{i0} are numbers of helium complexes with and without laser irradiation.[16] For irradiation OPO/OPA system from LaserVision was used.
The energy resolved collision induced dissociation (CID) experiments were performed on LCQ Deca mass spectrometer with an ESI source. The collision energies in the LCQ ion trap was calibrated based on the measurements of dissociation energies of series of thermometer ion consisting of benzylpyridinium and benzhydrylpyridinium thermometer ions using Schroder's method.[4–6] The complexes were measured for 2-4 times to calculate the standard deviation.

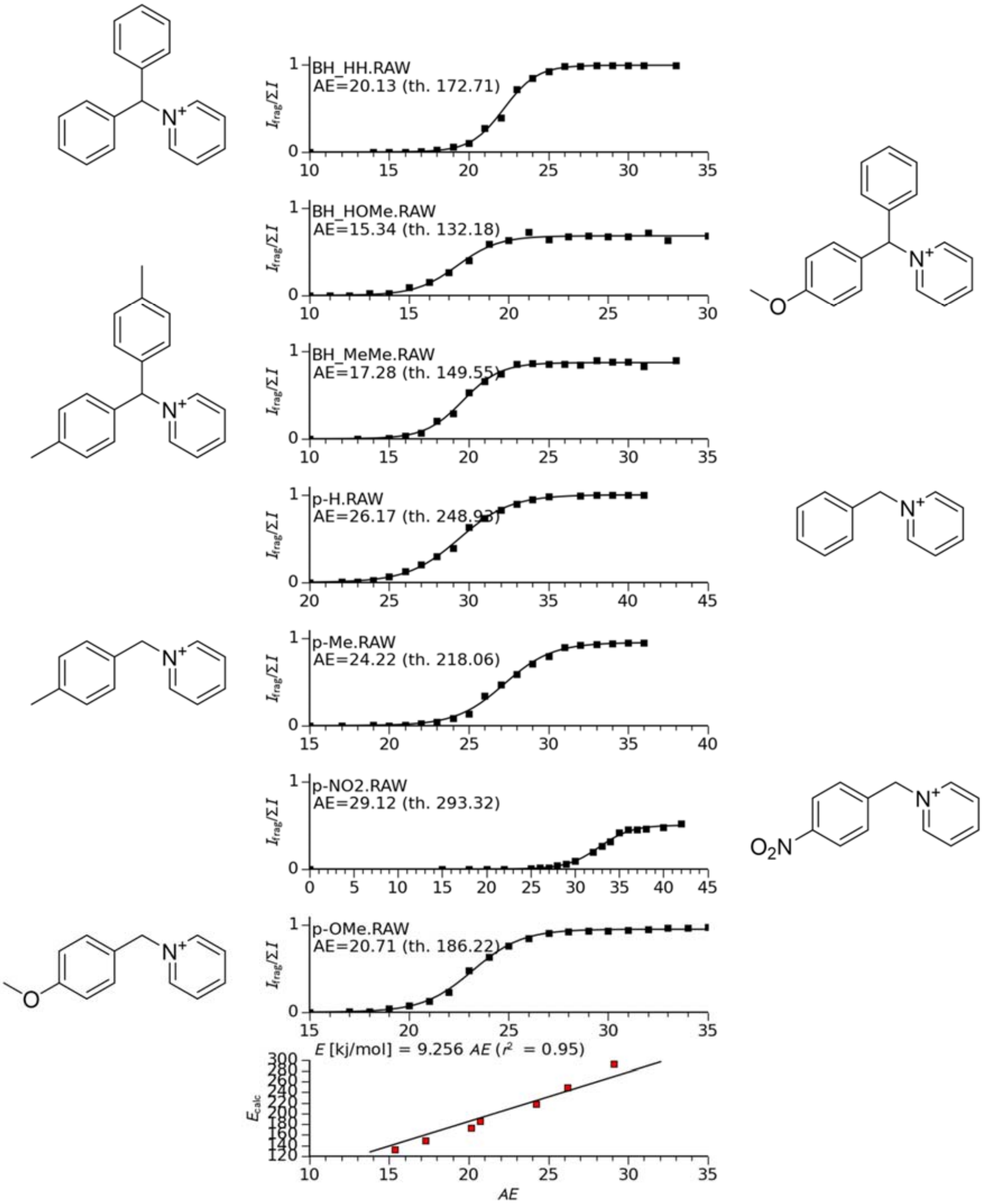

Figure S1: Calibration of the ion trap instrument using the thermometer ions (appearance energy i.e. AE for a set of thermometer ions is plotted against the known bond dissociation energy).

ESI spectrum of various ligands from Table 1 with AuX_3 (Source spectrum, CIDs and(/or) energy resolved CIDs)

1) 2,2'-bipyridine with $AuCl_3$

Sample preparation: 100 µL of 1 mM $AuCl_3$ in Acetonitrile (ACN) + 100 µL of 1 mM bipyridine (bipy) in dichloromethane (DCM) + 0.8 mL DCM.

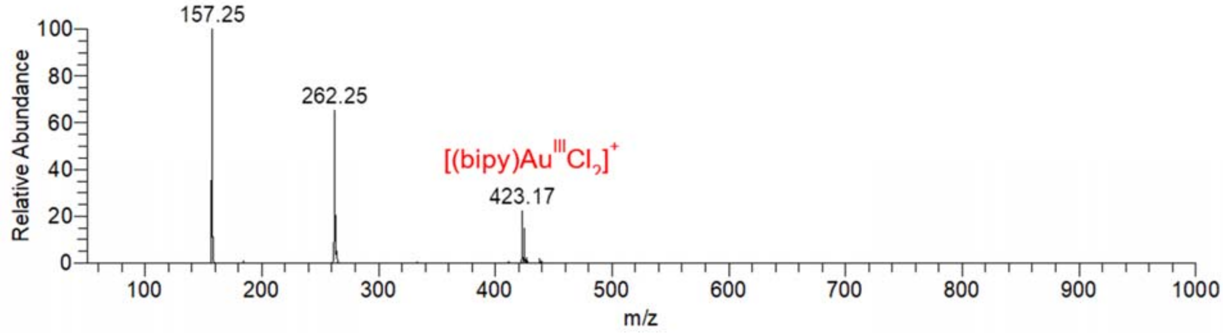

Figure S2: Source spectrum of 2,2'-bipyridine with $AuCl_3$

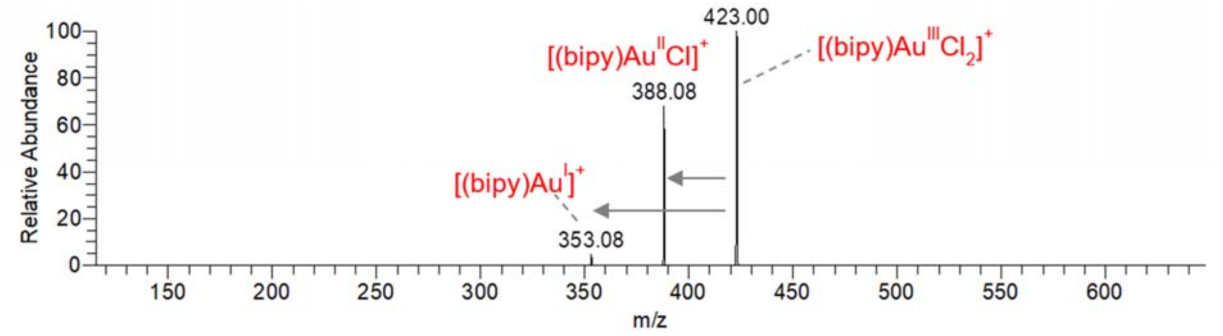

Figure S3: CID of *m/z* 423 at C.E = 16.5%

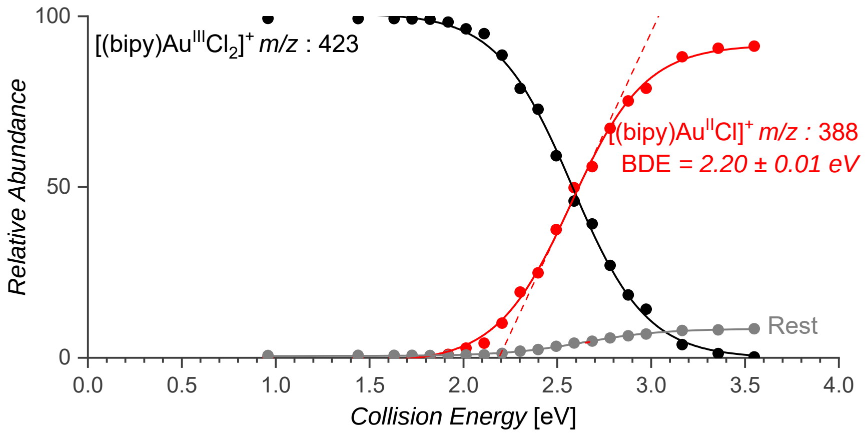

Figure S4: Energy Resolved CID of *m/z* 423

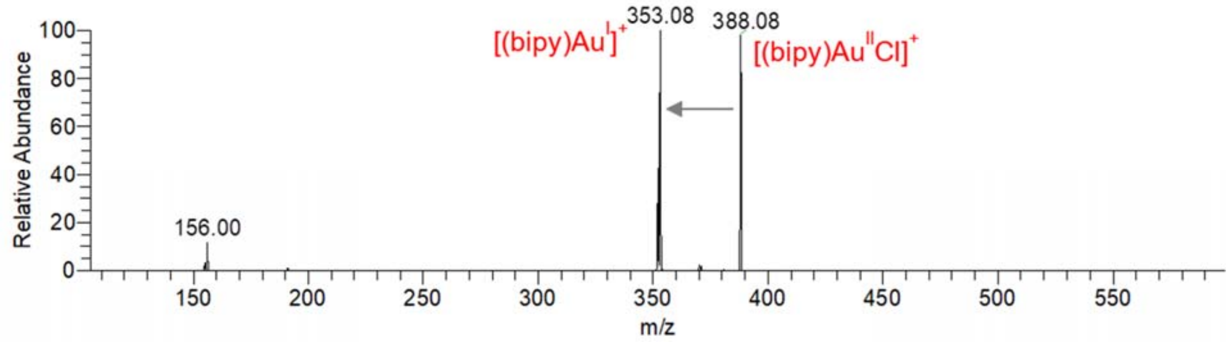

Figure S5: CID of *m/z* 388 at C.E = 29%

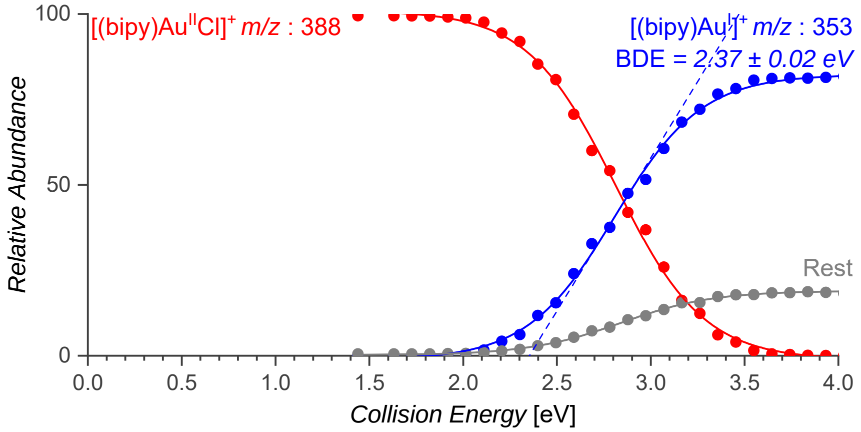

Figure S6: Energy Resolved CID of *m/z* 388

2) 2,2'-bipyridine with $AuBr_3$

Sample preparation: 100 µL of 1 mM $AuBr_3$ in Acetonitrile (ACN) + 100 µL of 1 mM bipyridine (bipy) in dichloromethane (DCM) + 0.8 mL DCM.

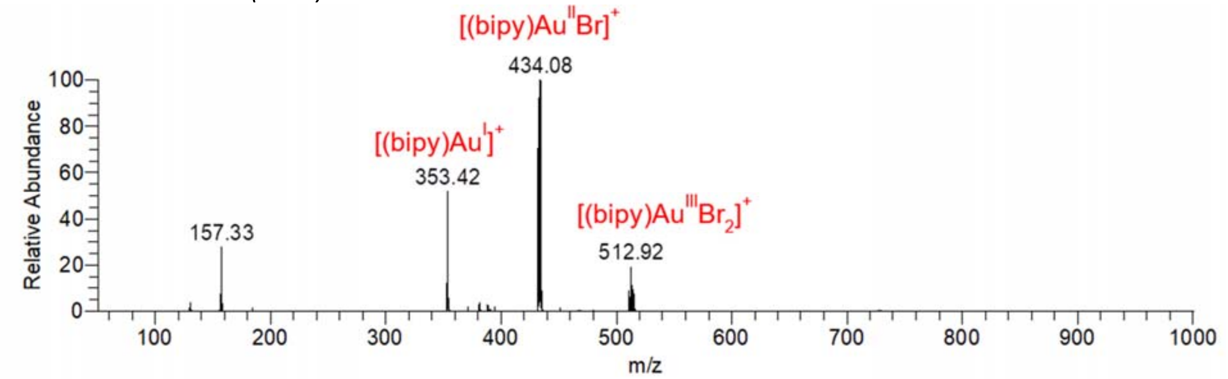

Figure S7: Source spectrum of 2,2'-bipyridine with $AuBr_3$

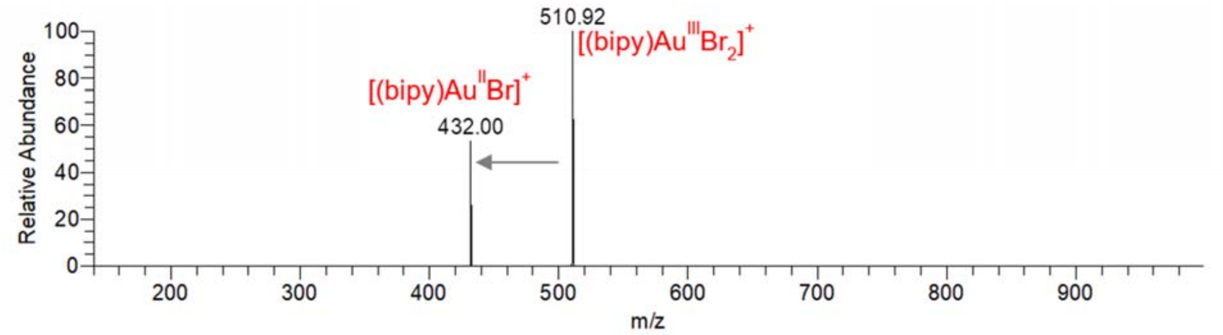

Figure S8: CID of *m/z* 511 at C.E = 13%

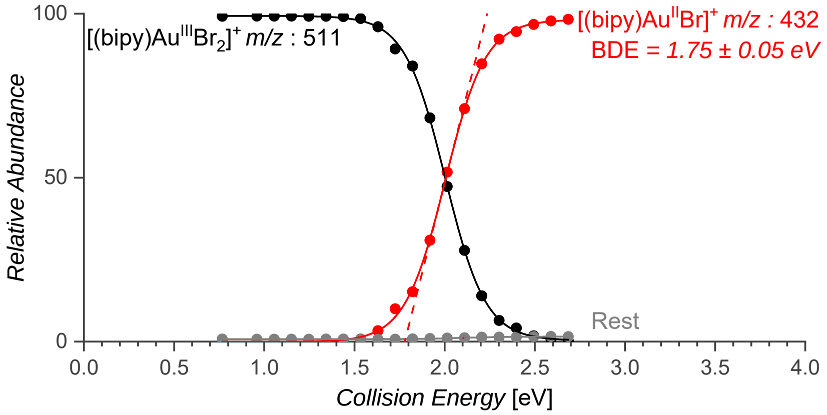

Figure S9: Energy Resolved CID of *m/z* 511

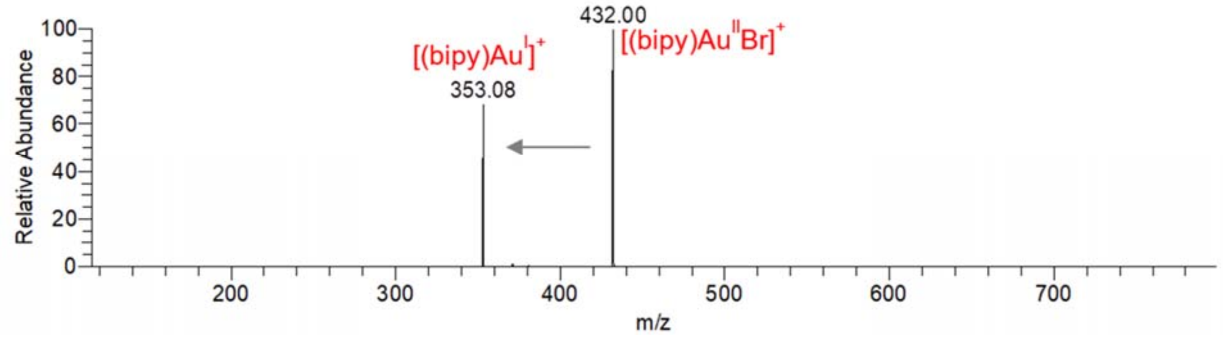

Figure S10: CID of *m/z* 432 at C.E = 15%

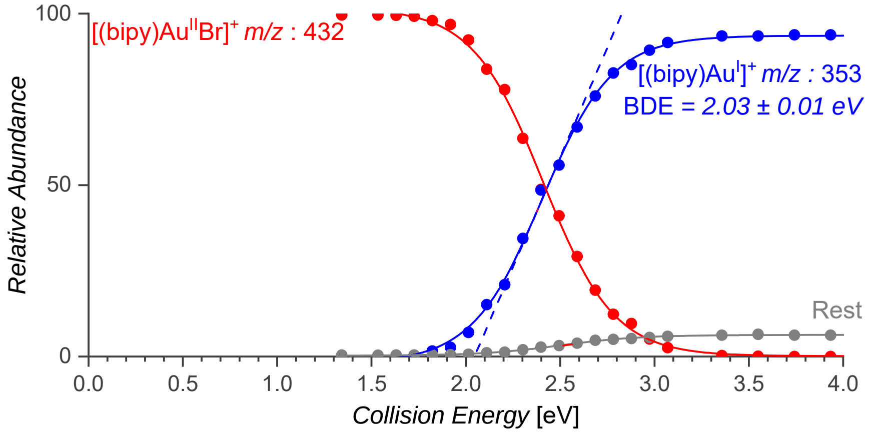

Figure S11: Energy Resolved CID of *m/z* 432

3) $[(bipy)AuCl_2]PF_6$ in acetone with excess of NaI

Sample preparation: 500 µL of 1 mM $[(bipy)AuCl_2]PF_6$ in acetone + 10 eq NaI, sonicated for 15min, filtered, filtrate + 0.5 mL DCM.

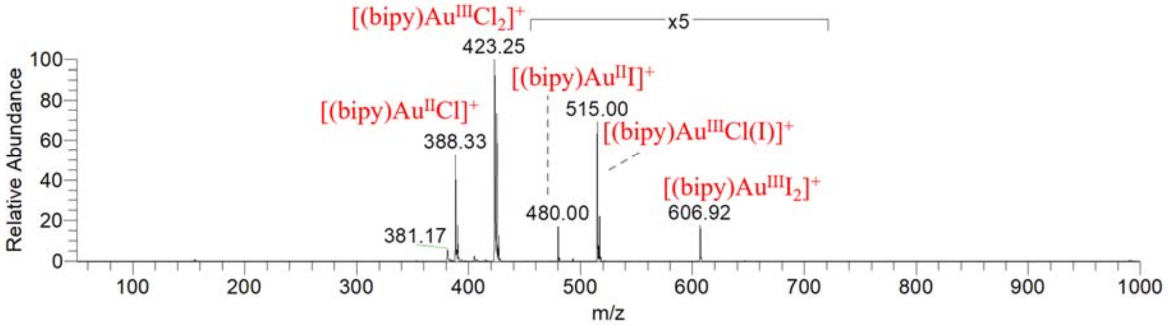

Figure S12: Source spectrum of $[(bipy)AuCl_2]PF_6$ with NaI

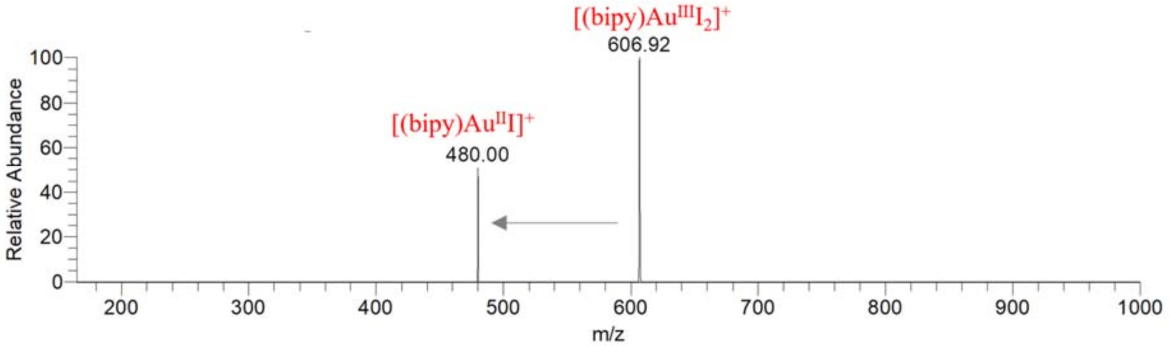

Figure S13: CID of *m/z* 607 at C.E = 20 %

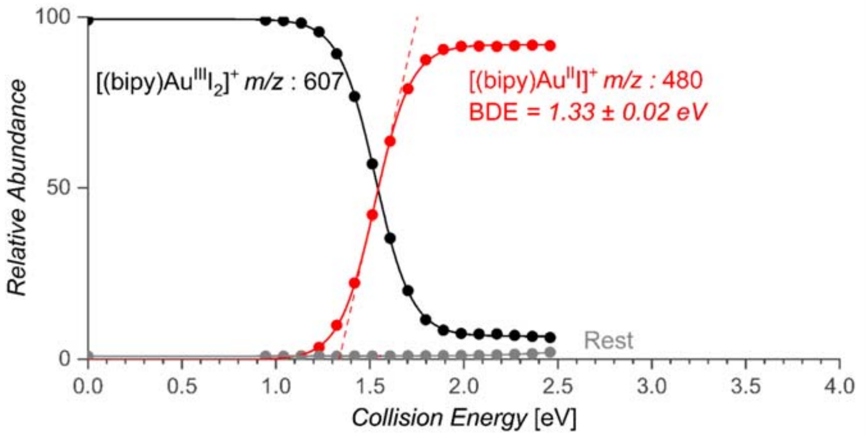

Figure S14: Energy Resolved CID of *m/z* 607

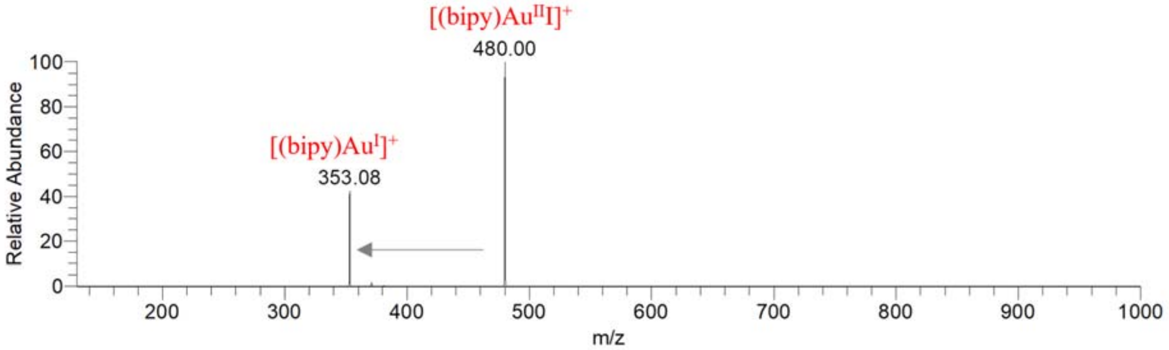

Figure S15: CID of *m/z* 480 at C.E = 13%

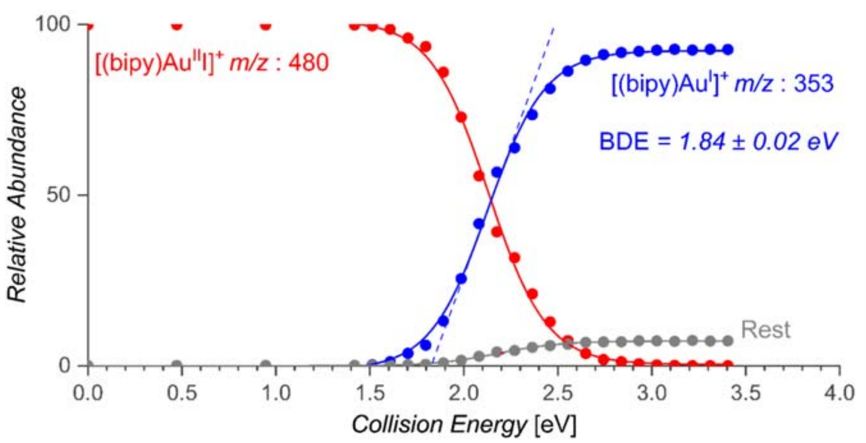

Figure S16: Energy Resolved CID of *m/z* 480

4) 2, 2':6', 2"-terpyridine with $AuCl_3$

Sample preparation: 100 µL of 1 mM $AuCl_3$ in Acetonitrile (ACN) + 100 µL of 1 mM terpyridine (terpy) in dichloromethane (DCM) + 0.8 mL DCM.

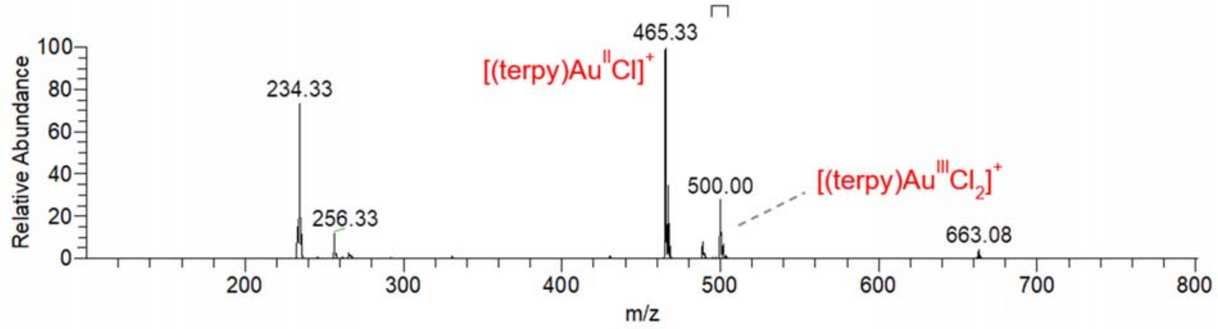

Figure S17: Source spectrum of 2, 2':6', 2"-terpyridine with $AuCl_3$

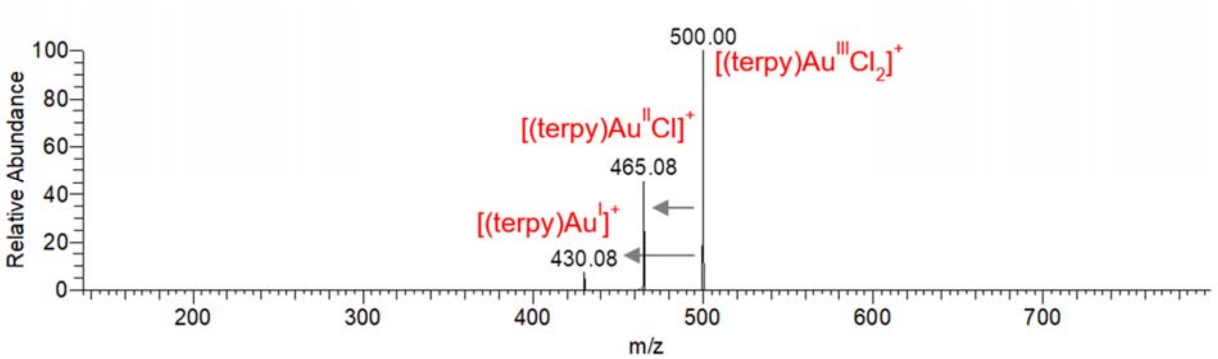

Figure S18: CID of *m/z* 500 at C.E = 13.5%

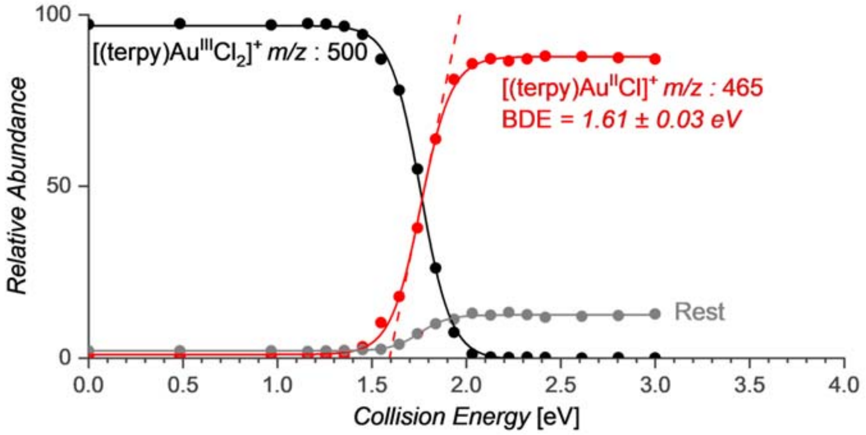

Figure S19: Energy Resolved CID of *m/z* 500

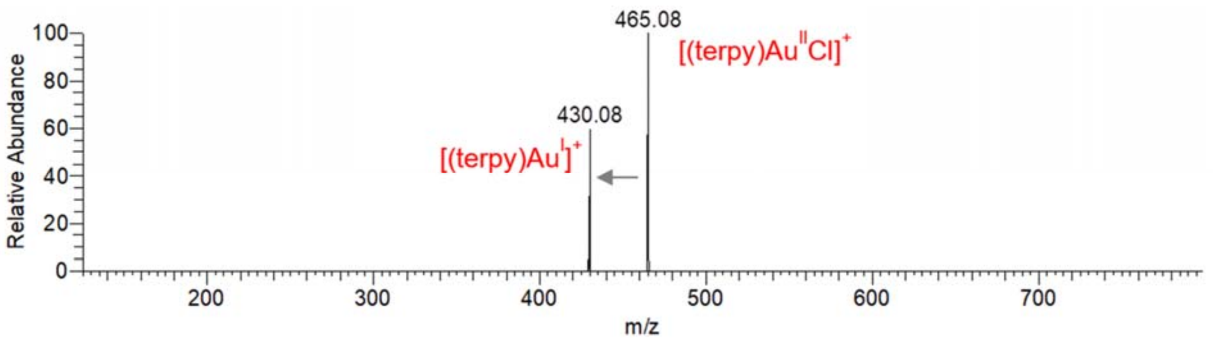

Figure S20: CID of *m/z* 465 at C.E = 16%

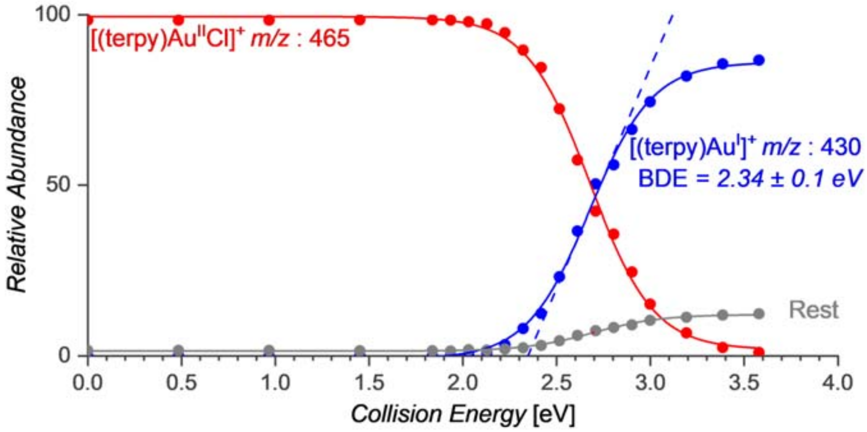

Figure S21: Energy Resolved CID of *m/z* 465

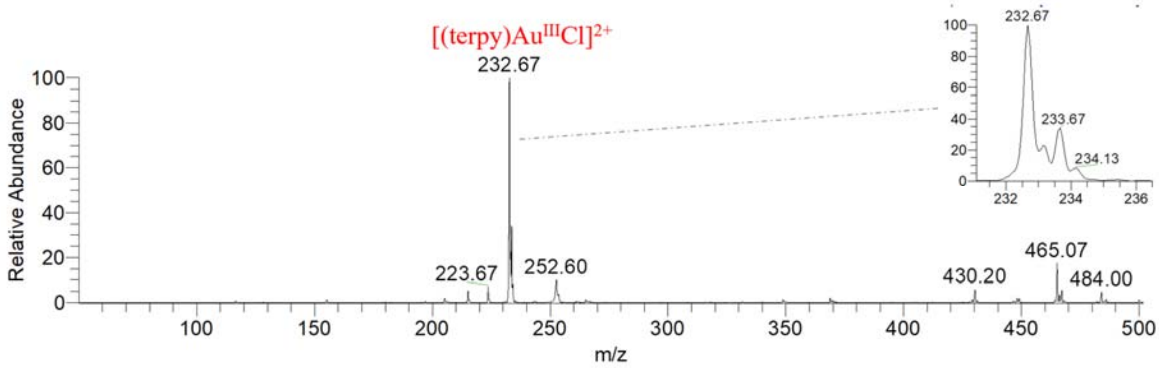

Figure S22: [Au(terpy)(Cl)](PF_6)$_2$ dissolved in acetonitrile

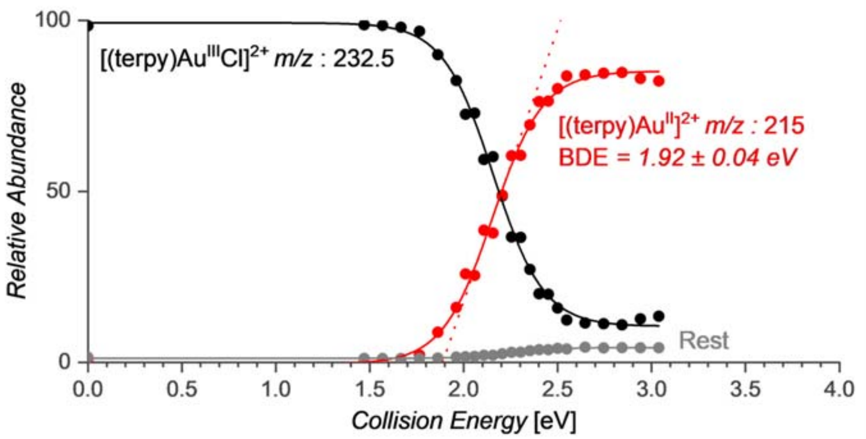

Figure S23: Energy Resolved CID of *m/z* 232.5

5) 2, 2':6', 2"-terpyridine with $AuBr_3$

Sample preparation: 100 µL of 1 mM $AuBr_3$ in Acetonitrile (ACN) + 100 µL of 1 mM terpyridine (terpy) in dichloromethane (DCM) + 0.8 mL DCM.

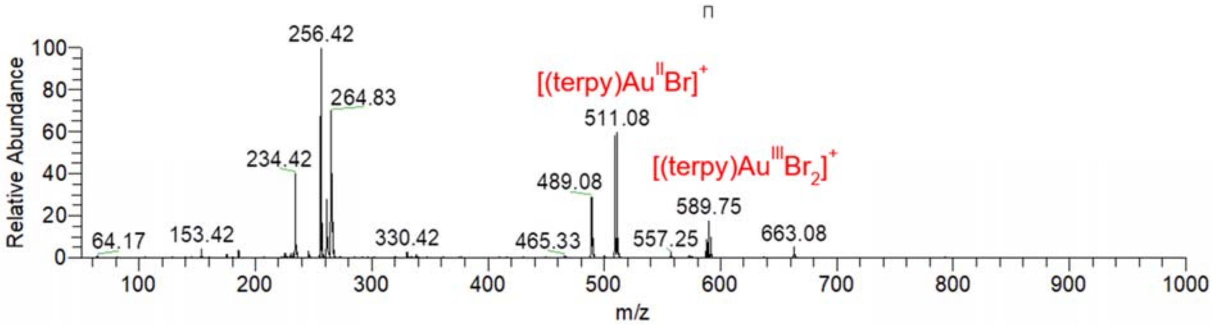

Figure S24: Source spectrum of 2, 2':6', 2"-terpyridine with $AuBr_3$

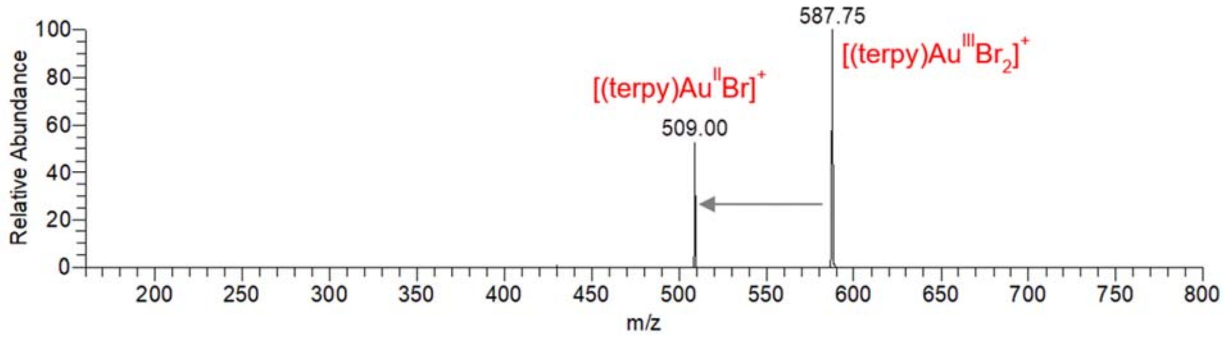

Figure S25: CID of *m/z* 588 at C.E = 13%

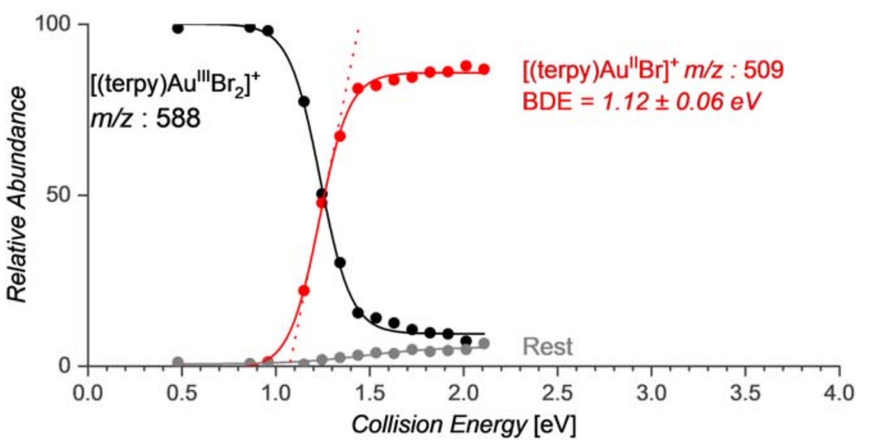

Figure S26: Energy Resolved CID of *m/z* 588

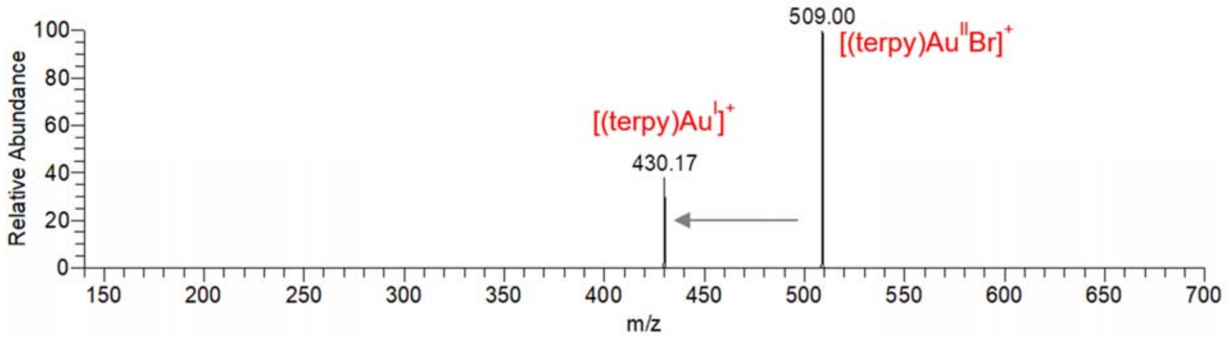

Figure S27: CID of *m/z* 509 at C.E = 13.5%

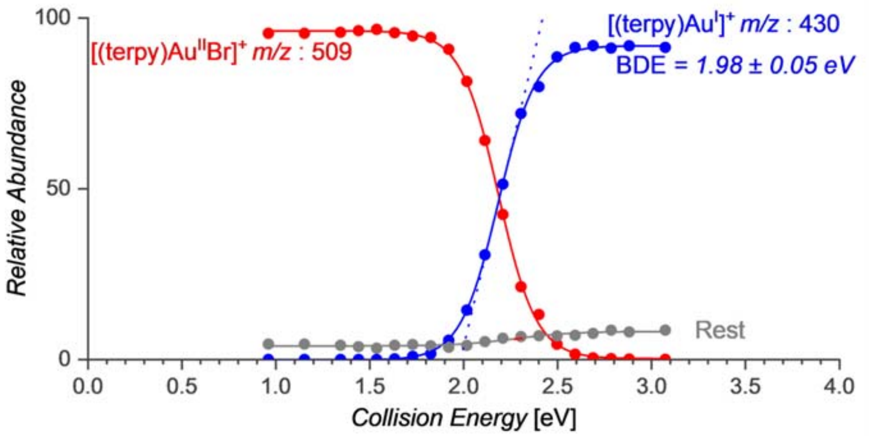

Figure S28: Energy Resolved CID of *m/z* 509

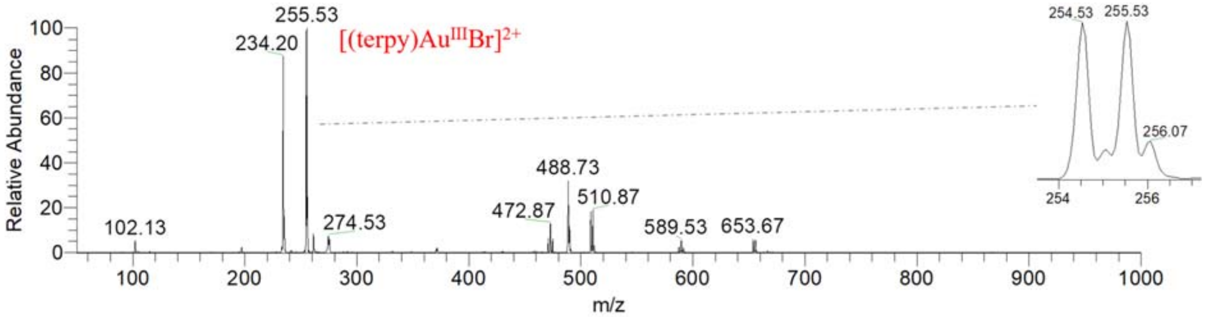

Figure S29: $[Au(terpy)(Br)](PF_6)_2$ dissolved in acetonitrile

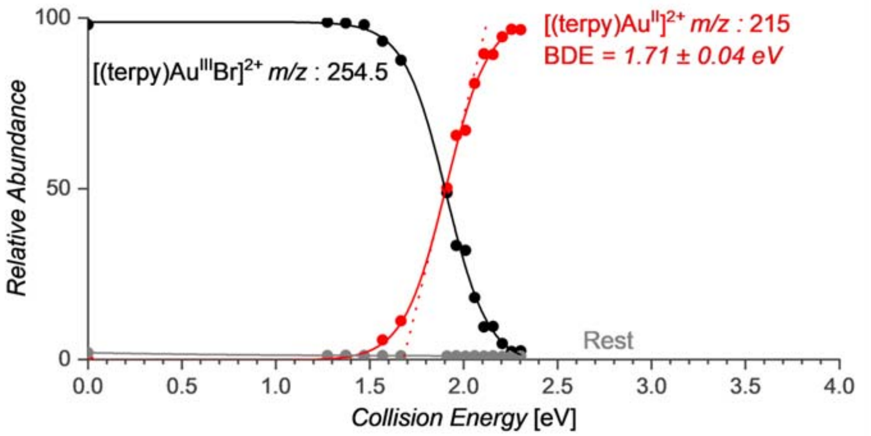

Figure S30: Energy Resolved CID of *m/z* 254.5

6) **1,10-Phenanthroline with $AuCl_3$**

Sample preparation: 100 µL of 1 mM $AuCl_3$ in Acetonitrile (ACN) + 100 µL of 1 mM 1,10-Phenanthroline in dichloromethane (DCM) + 0.8 mL DCM.

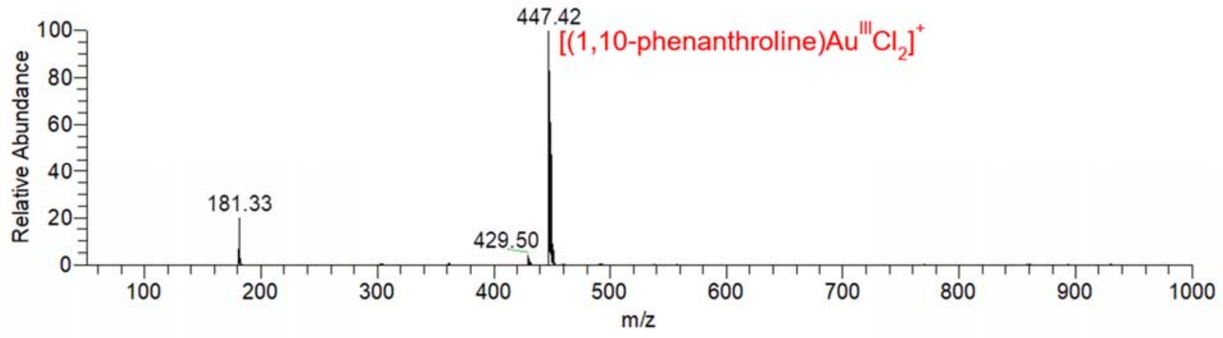

Figure S31: Source spectrum of 1,10-Phenanthroline with $AuCl_3$

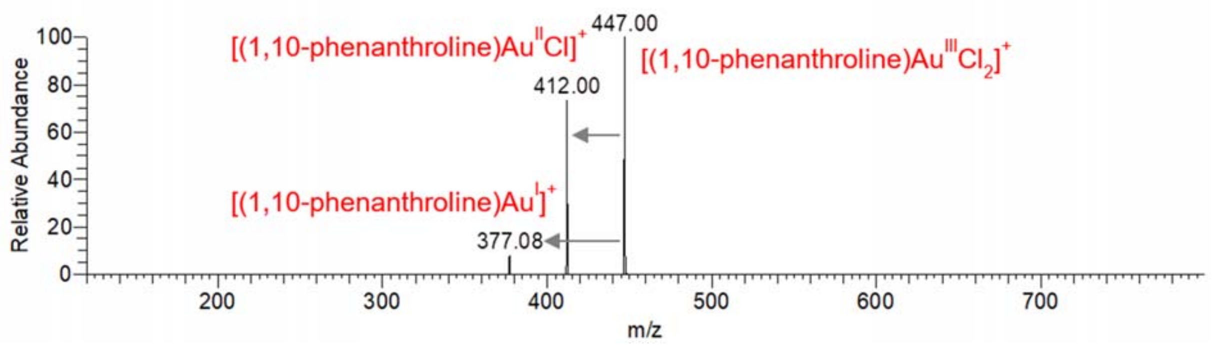

Figure S32: CID of *m/z* 447 at C.E = 16%

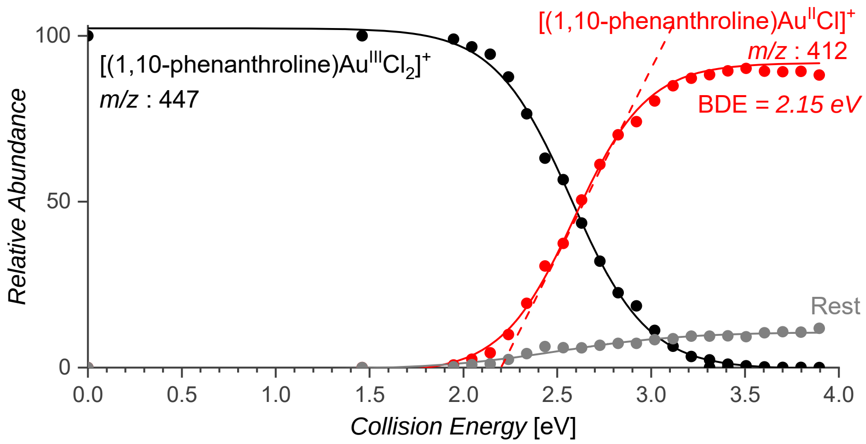

Figure S33: Energy Resolved CID of *m/z* 447

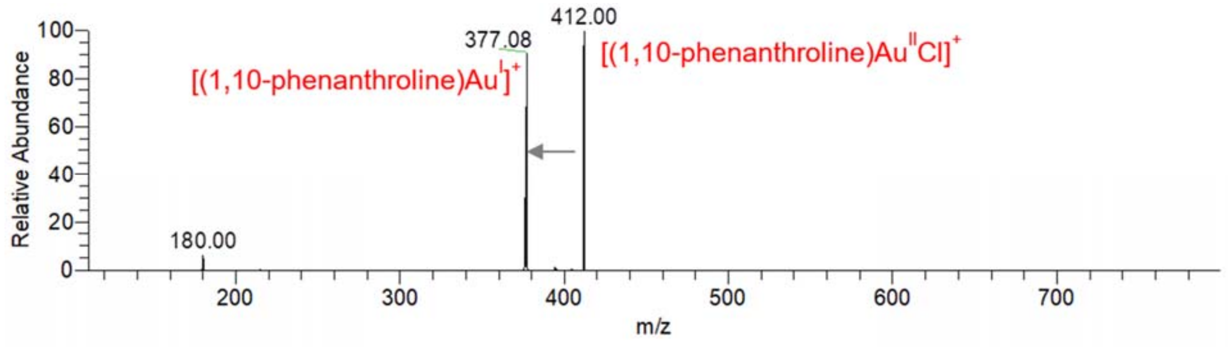

Figure S34: CID of *m/z* 412 at C.E = 27%

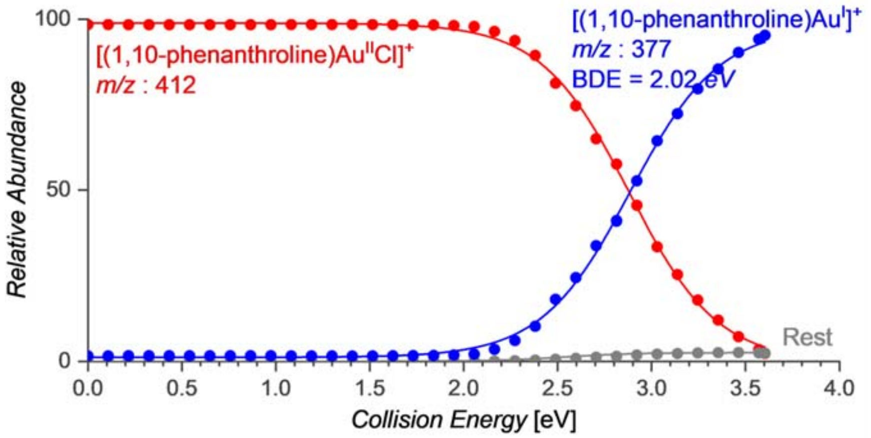

Figure S35: Energy Resolved CID of *m/z* 412

7) 1,10-Phenanthroline with $AuBr_3$

Sample preparation: 100 µL of 1 mM $AuBr_3$ in Acetonitrile (ACN) + 100 µL of 1 mM 1,10-Phenanthroline in dichloromethane (DCM) + 0.8 mL DCM.

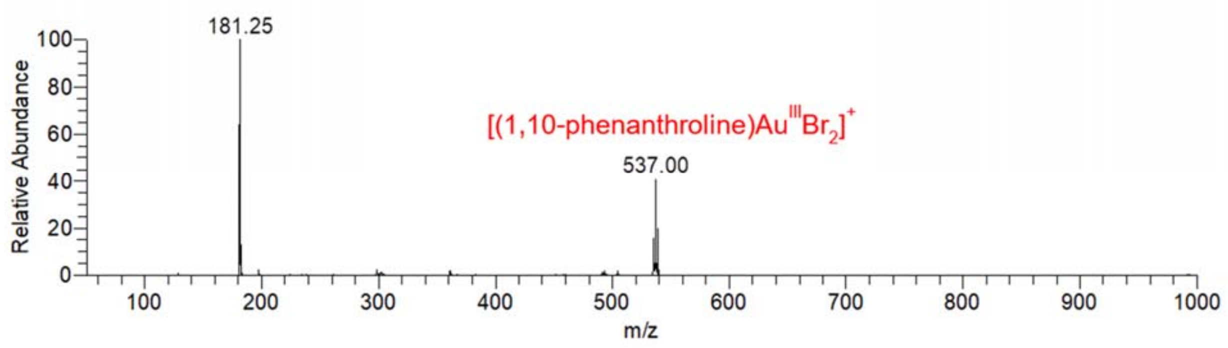

Figure S36: Source spectrum of 1,10-Phenanthroline with $AuBr_3$

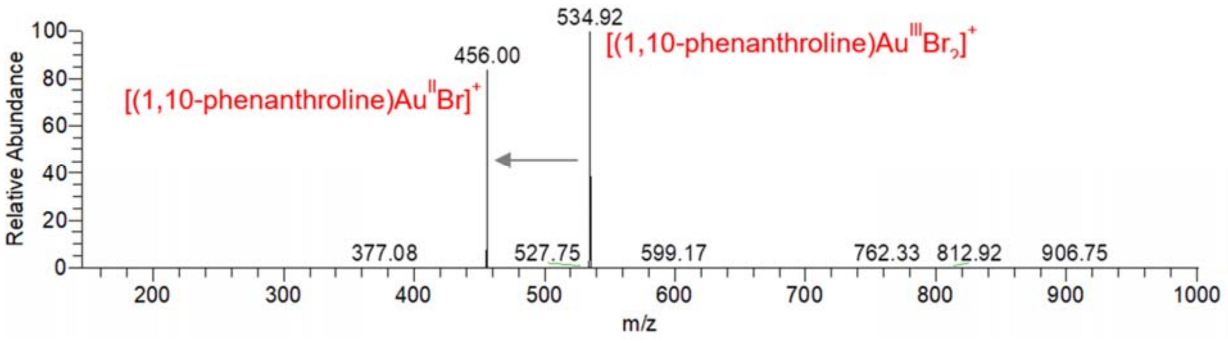

Figure S37: CID of *m/z* 535 at C.E = 14%

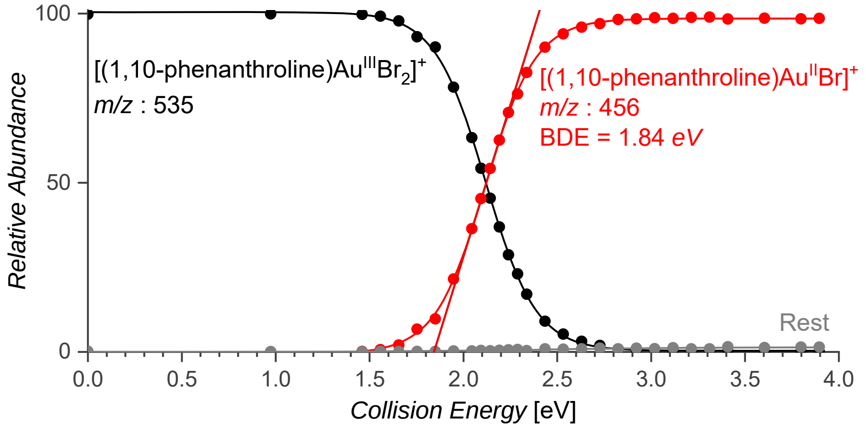

Figure S38: Energy Resolved CID of *m/z* 535

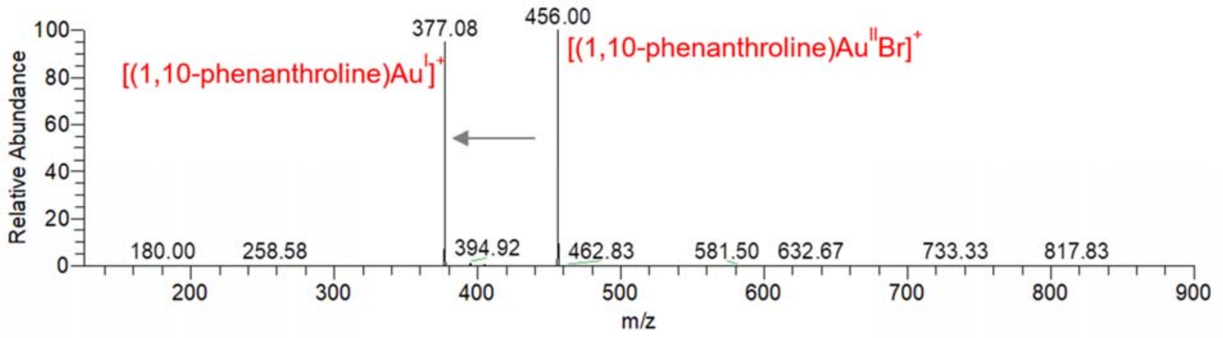

Figure S39: CID of *m/z* 456 at C.E = 15%

8) Pyridine with $AuCl_3$

Sample preparation: 100 µL of 1 mM $AuCl_3$ in Acetonitrile (ACN) + 100 µL of 1 mM pyridine in dichloromethane (DCM) + 0.8 mL DCM.

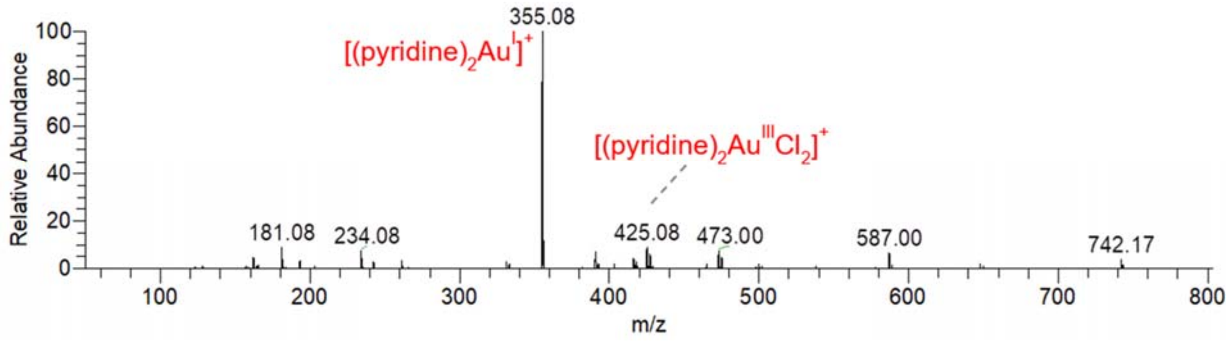

Figure S40: Source spectrum of Pyridine with $AuCl_3$

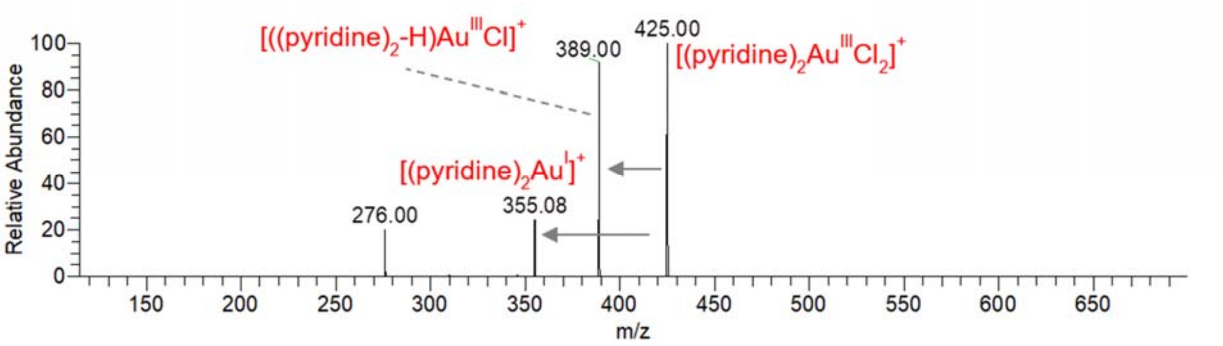

Figure S41: CID of *m/z* 425 at C.E = 17%

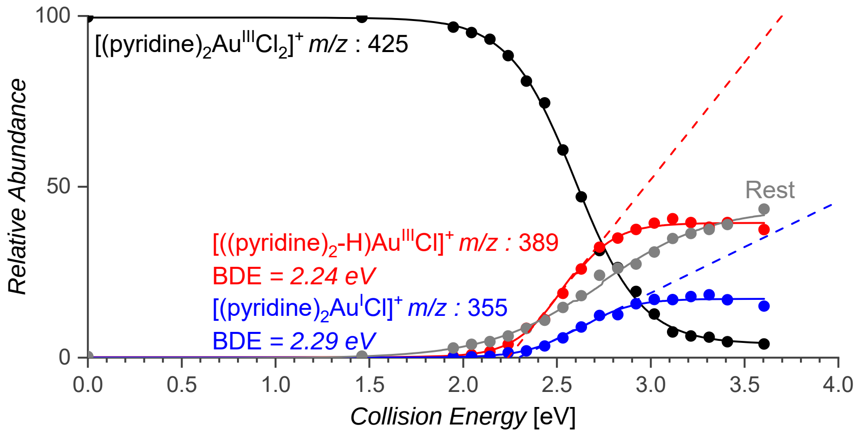

Figure S42: Energy Resolved CID of *m/z* 425

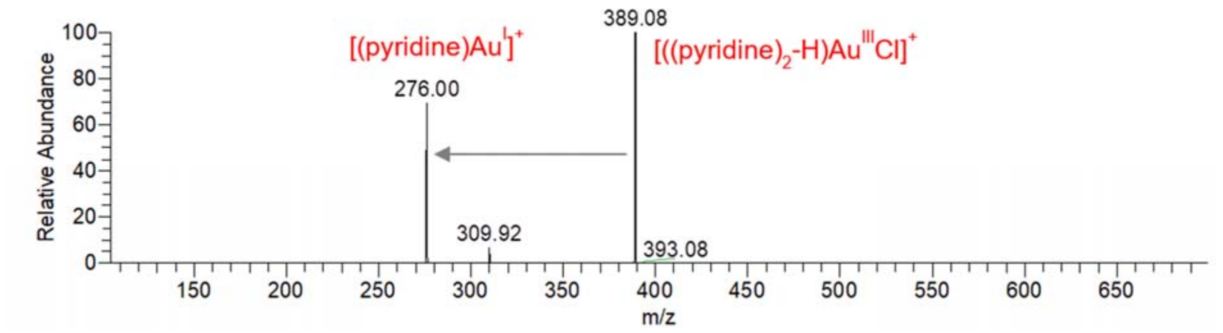

Figure S43: CID of *m/z* 389 at C.E = 20%

9) Pyridine with $AuBr_3$

Sample preparation: 100 µL of 1 mM $AuBr_3$ in Acetonitrile (ACN) + 100 µL of 1 mM pyridine in dichloro-methane (DCM) + 0.8 mL DCM.

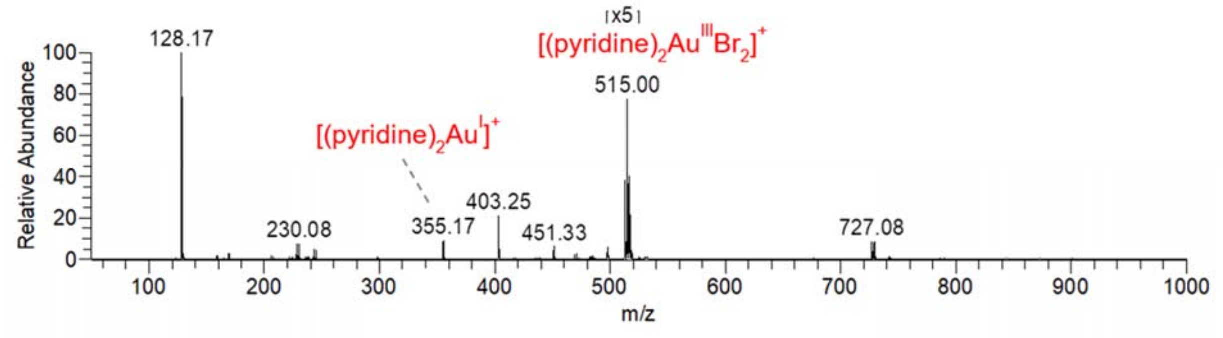

Figure S44: Source spectrum of Pyridine with $AuBr_3$

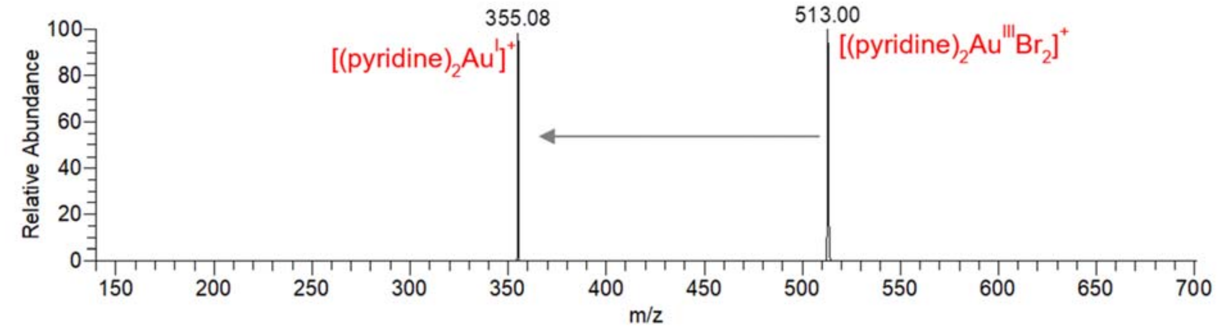

Figure S45: CID of *m/z* 513 at C.E = 13%

10) 1,1-Bis(diphenylphosphino)methane with $AuCl_3$

Sample preparation: 100 µL of 1 mM $AuCl_3$ in Acetonitrile (ACN) + 300 µL of 1 mM 1,1-Bis(diphenylphosphino)methane (dppm) in dichloromethane (DCM) + 3.6 mL DCM.

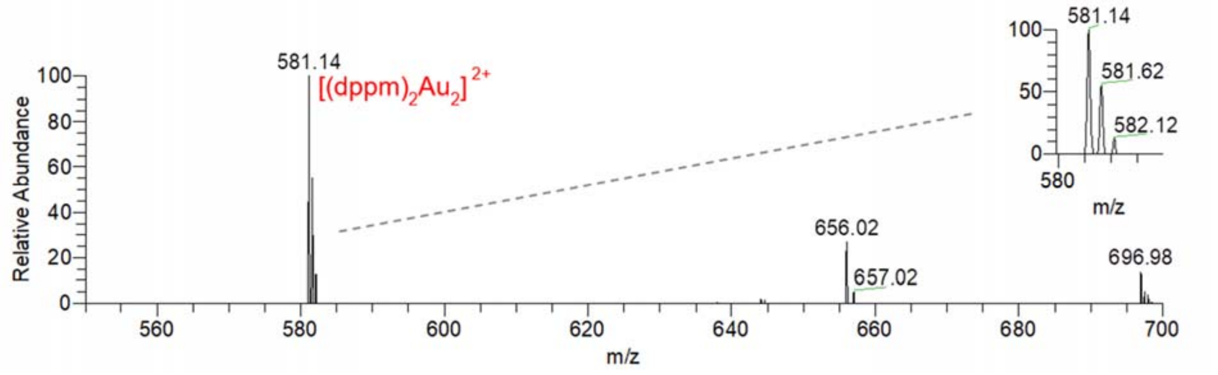

Figure S46: Source spectrum of 1,1-Bis(diphenylphosphino)methane with $AuCl_3$

11) 1,2-Bis(diphenylphosphino)ethane with $AuCl_3$

Sample preparation: 100 µL of 1 mM $AuCl_3$ in Acetonitrile (ACN) + 100 µL of 1 mM 1,2-Bis(diphenylphosphino)ethane (dppe) in dichloromethane (DCM) + 0.8 mL DCM.

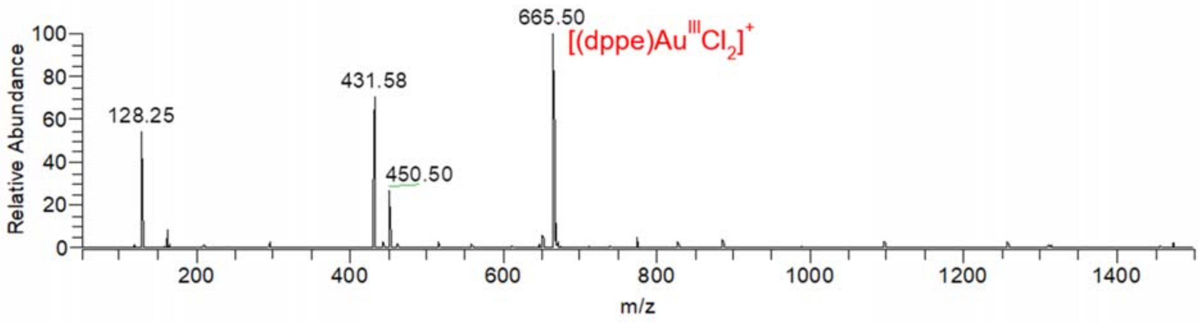

Figure S47: Source spectrum of 1,1-Bis(diphenylphosphino)ethane with $AuCl_3$

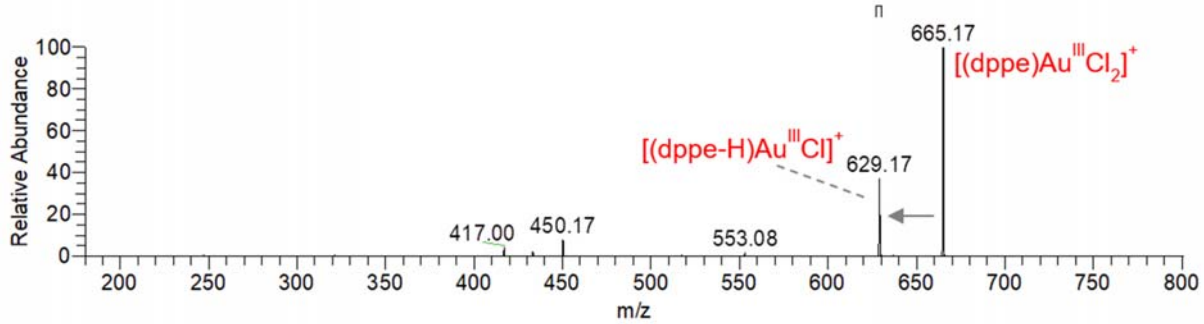

Figure S48: CID of *m/z* 665 at C.E = 19%

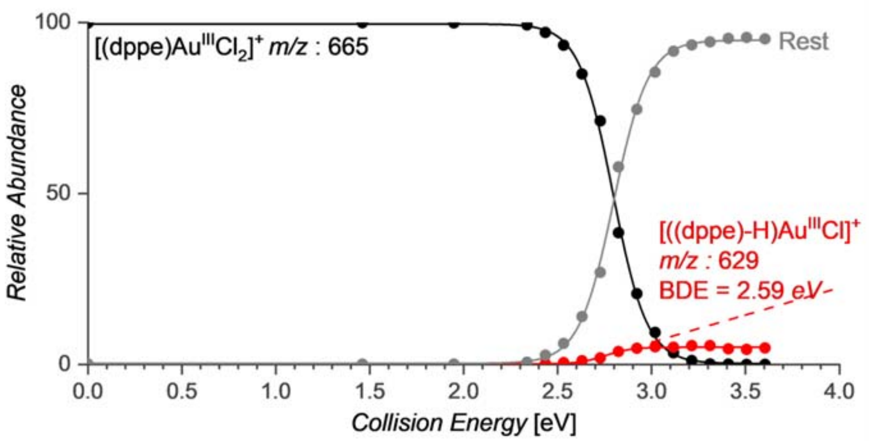

Figure S49: Energy Resolved CID of *m/z* 665

12) 1,2-Bis(diphenylphosphino)ethane with $AuBr_3$

Sample preparation: 100 µL of 1 mM $AuBr_3$ in Acetonitrile (ACN) + 100 µL of 1 mM 1,2-Bis(diphenylphosphino)ethane (dppe) in dichloromethane (DCM) + 0.8 mL DCM.

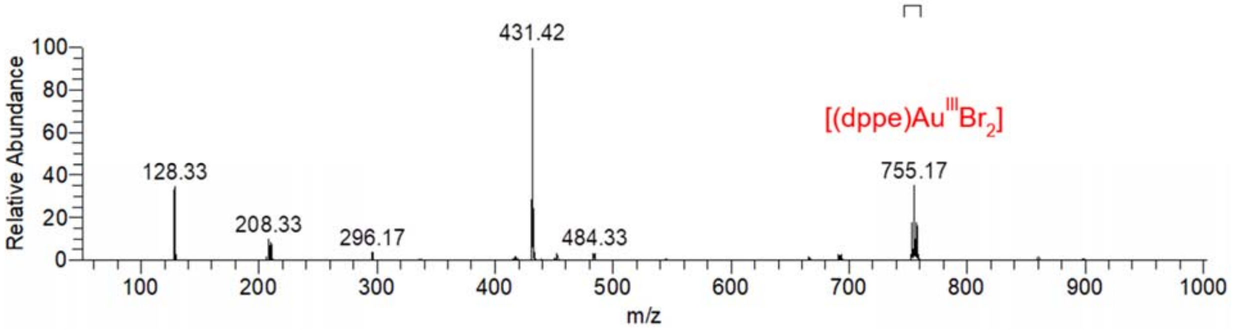

Figure S50: Source spectrum of 1,2-Bis(diphenylphosphino)ethane with $AuBr_3$

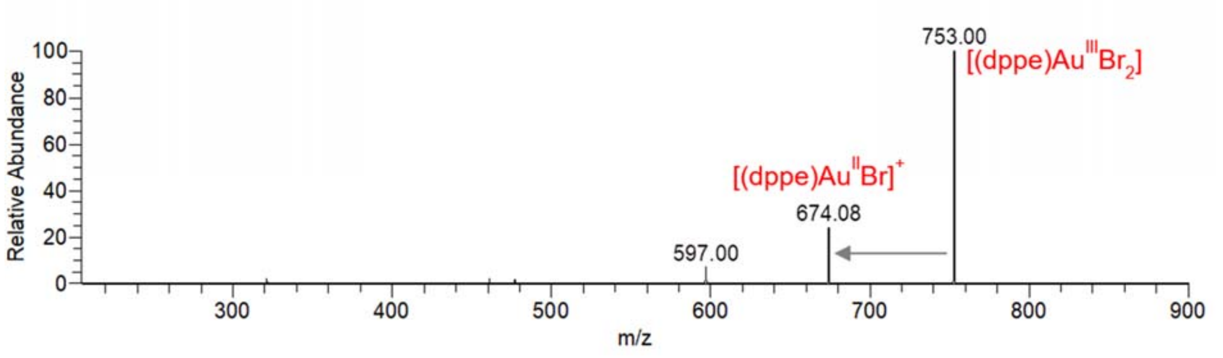

Figure S51: CID of *m/z* 753 at C.E = 21%

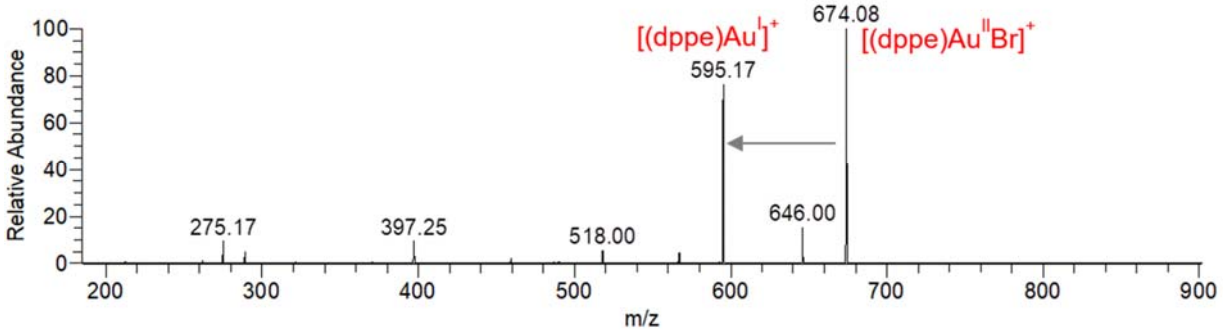

Figure S52: CID MS^3 of *m/z* 674 at C.E = 20%

13) 1,3-Bis(diphenylphosphino)propane with $AuCl_3$

Sample preparation: 100 µL of 1 mM $AuCl_3$ in Acetonitrile (ACN) + 100 µL of 1 mM 1,3-Bis(diphenylphosphino)propane (dppp) in dichloromethane (DCM) + 0.8 mL DCM.

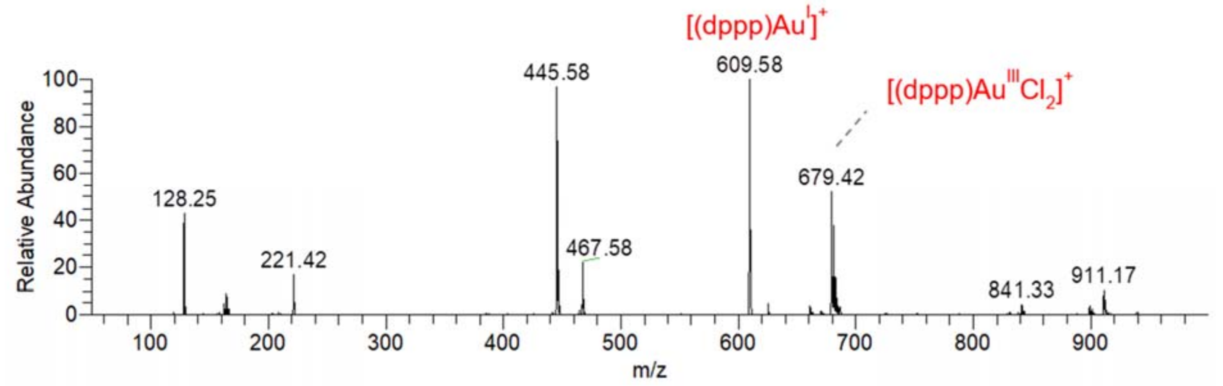

Figure S53: Source spectrum of 1,3-Bis(diphenylphosphino)propane with $AuCl_3$

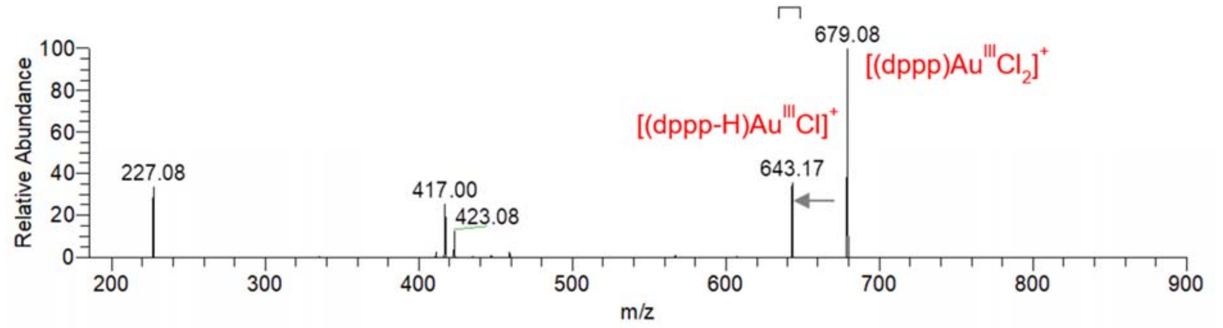

Figure S54: CID of *m/z* 679 at C.E = 21%

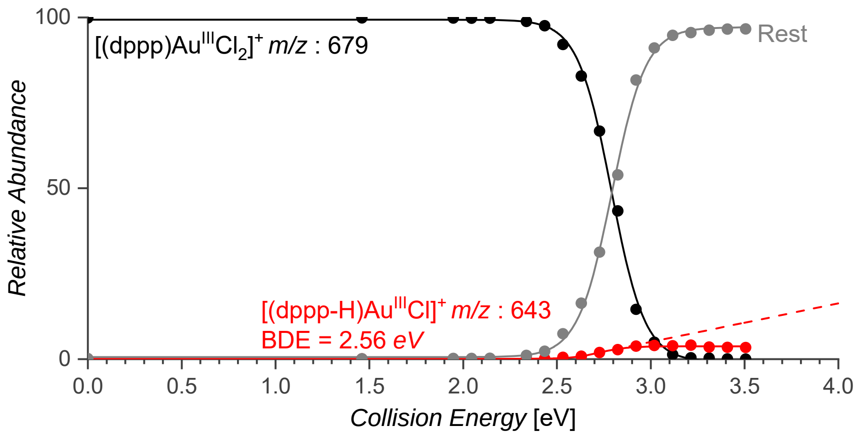

Figure S55: Energy Resolved CID of *m/z* 679

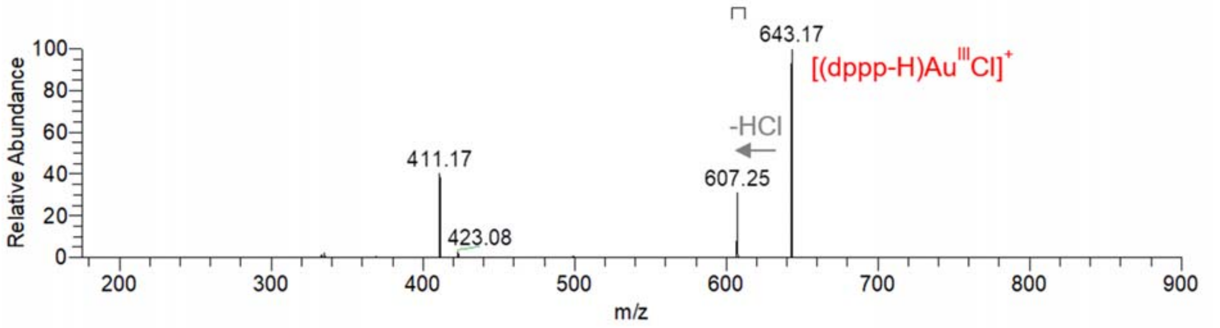

Figure S56: CID MS^3 of *m/z* 643 at C.E = 19%

14) 1,3-Bis(diphenylphosphino)propane with $AuBr_3$

Sample preparation: 100 µL of 1 mM $AuBr_3$ in Acetonitrile (ACN) + 100 µL of 1 mM 1,3-Bis(diphenylphosphino)propane (dppp) in dichloromethane (DCM) + 0.8 mL DCM.

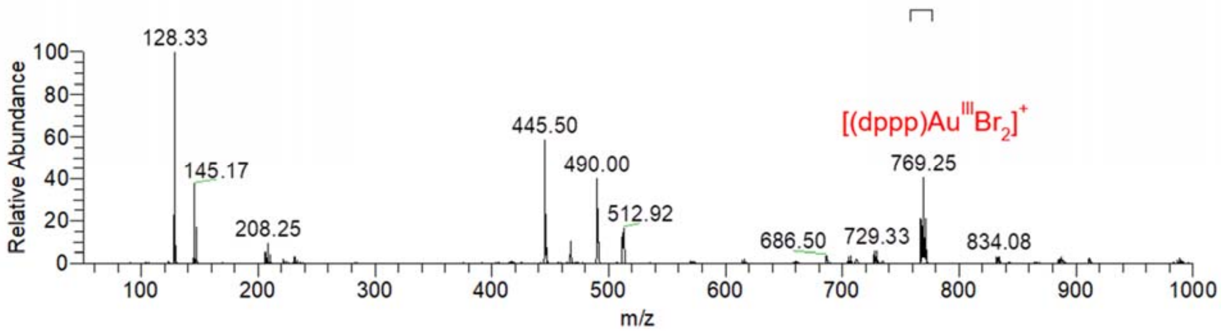

Figure S57: Source spectrum of 1,3-Bis(diphenylphosphino)propane with $AuBr_3$

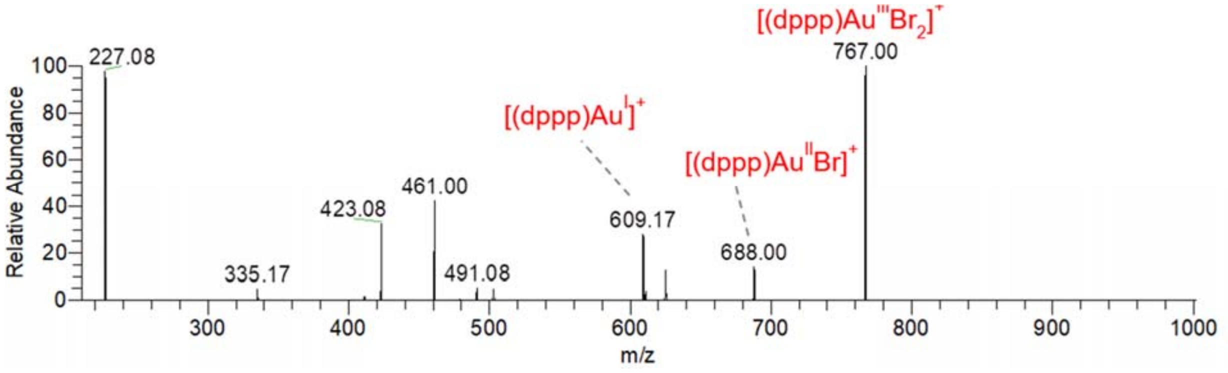

Figure S58: CID of *m/z* 767 at C.E = 24%

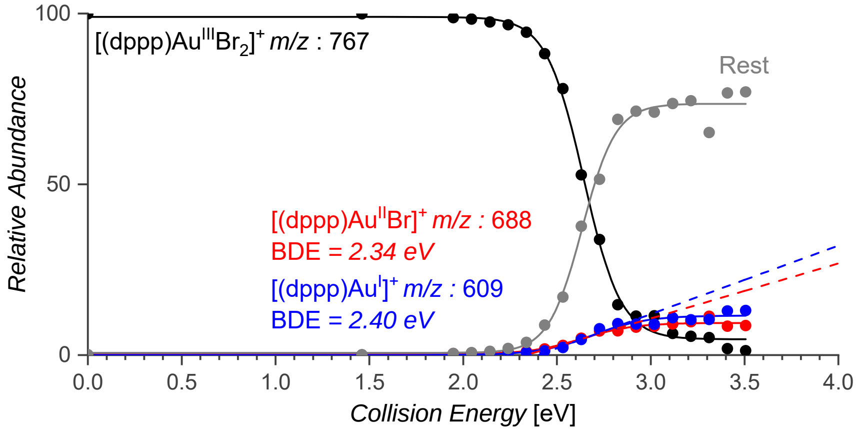

Figure S59: Energy Resolved CID of *m/z* 767

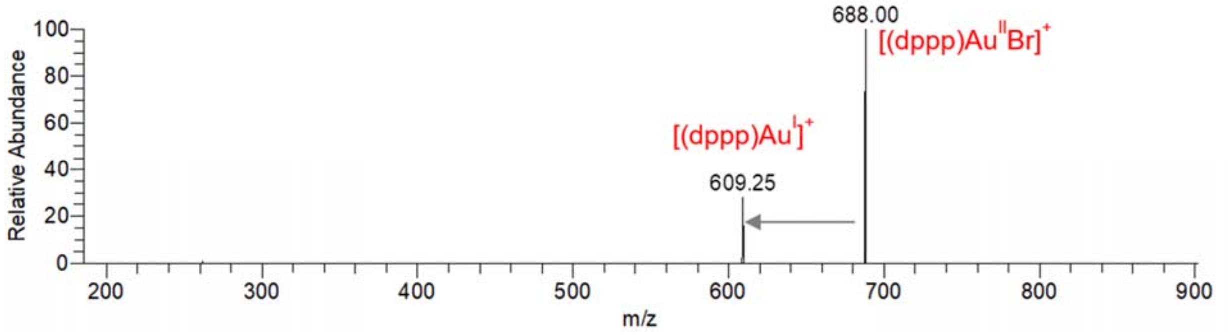

Figure S60: CID MS^3 of *m/z* 688 at C.E = 22.5%

15) 1,4-Bis(diphenylphosphino)butane with $AuCl_3$

Sample preparation: 100 µL of 1 mM $AuCl_3$ in Acetonitrile (ACN) + 300 µL of 1 mM 1,4-Bis(diphenylphosphino)butane (dppb) in dichloromethane (DCM) + 3.6 mL DCM.

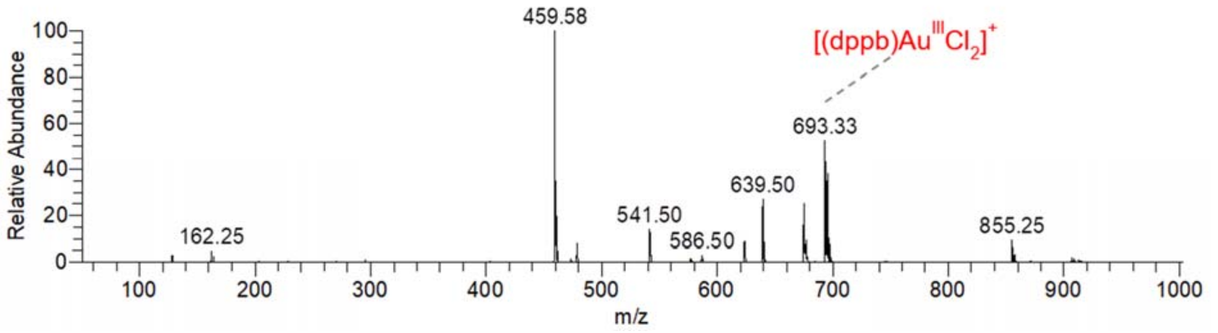

Figure S61: Source spectrum of 1,4-Bis(diphenylphosphino)butane with $AuCl_3$

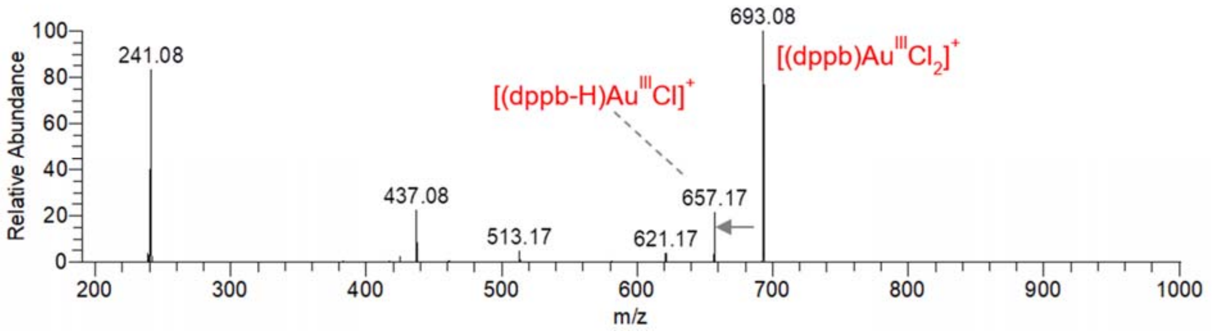

Figure S62: CID of *m/z* 693 at C.E = 22%

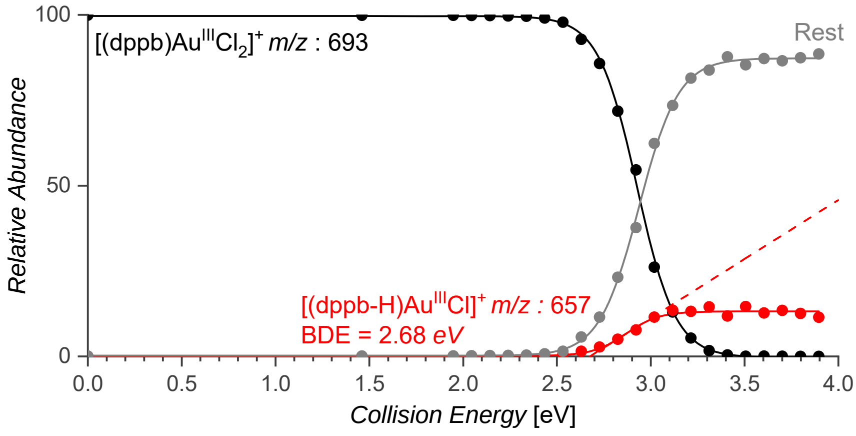

Figure S63: Energy Resolved CID of *m/z* 693

16) 1,4-Bis(diphenylphosphino)butane with $AuBr_3$

Sample preparation: 100 µL of 1 mM $AuBr_3$ in Acetonitrile (ACN) + 100 µL of 1 mM 1,4-Bis(diphenylphosphino)butane (dppb) in dichloromethane (DCM) + 0.8 mL DCM.

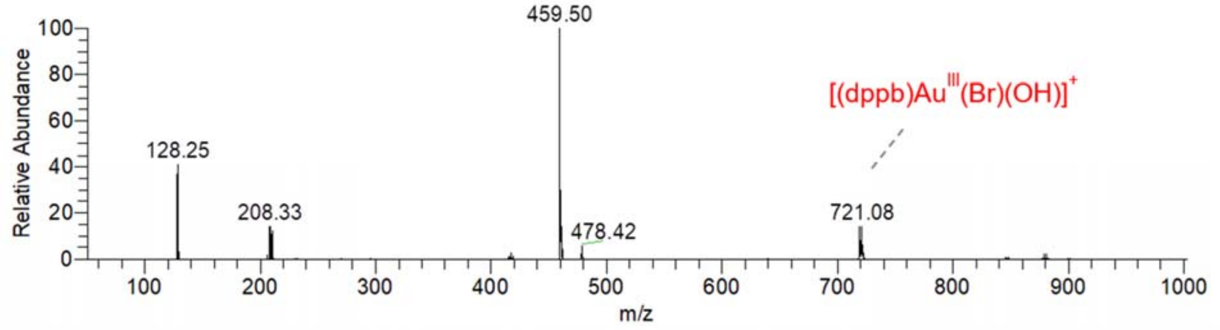

Figure S64: Source spectrum of 1,4-Bis(diphenylphosphino)butane with $AuBr_3$

$[(dppb)Au^{III}Br_2]^+$ not observed.

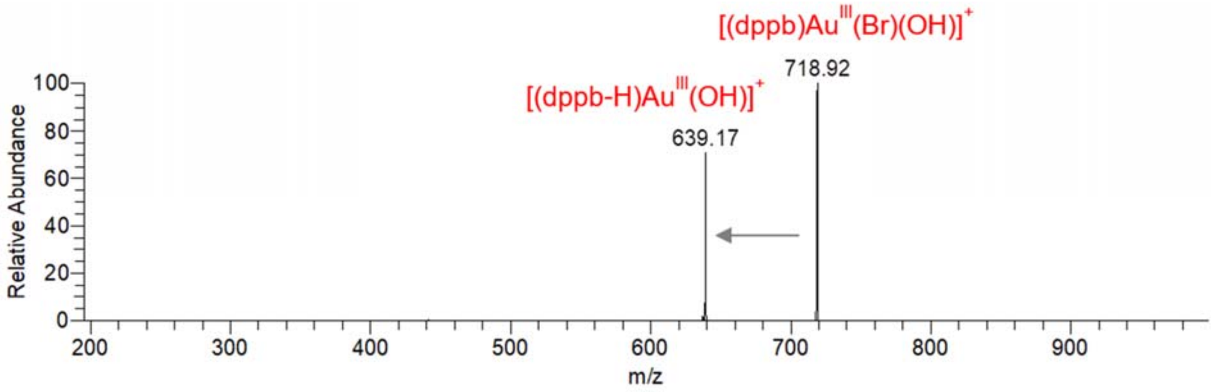

Figure S65: CID of *m/z* 719 at C.E = 21%

17) Tetramethylethylenediamine with $AuCl_3$

Sample preparation: 100 µL of 1 mM $AuCl_3$ in Acetonitrile (ACN) + 100 µL of 1 mM tetramethylethylenediamine (tmeda) in dichloromethane (DCM) + 0.8 mL DCM.

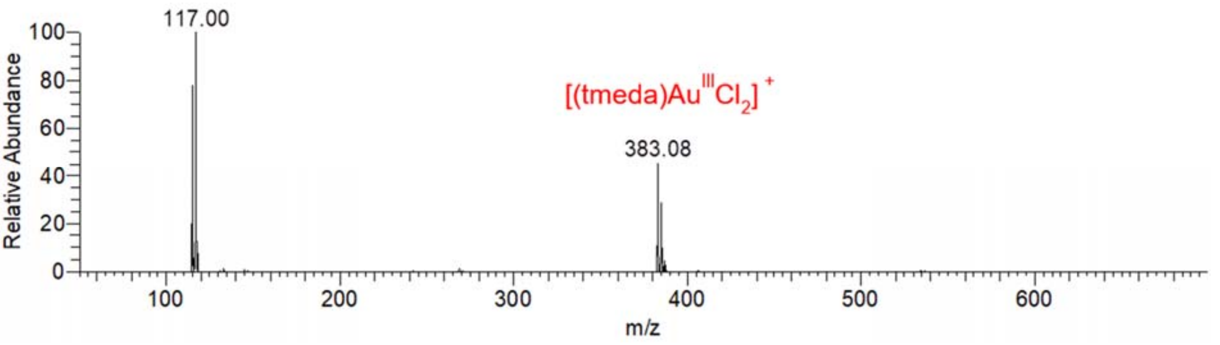

Figure S66: Source spectrum of Tetramethylethylenediamine with $AuCl_3$

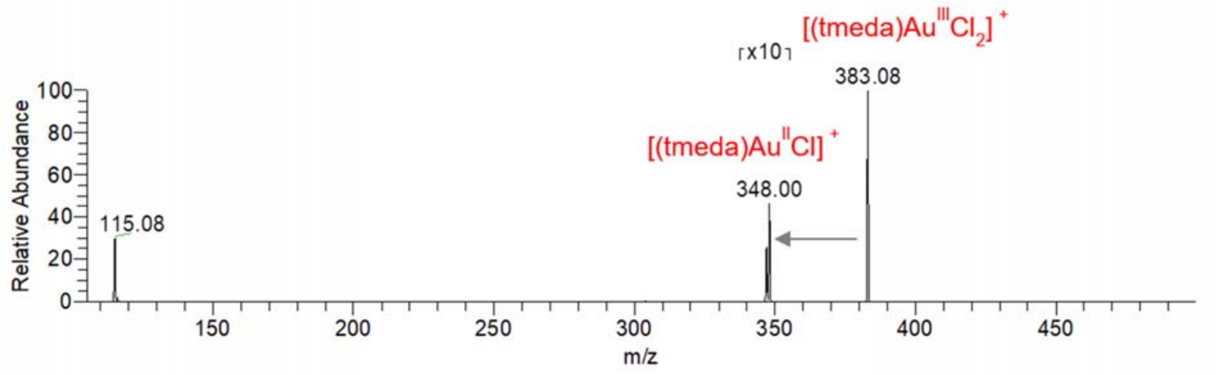

Figure S67: CID of *m/z* 383 at C.E = 22%

18) Tetramethylethylenediamine with $AuBr_3$

Sample preparation: 100 µL of 1 mM $AuBr_3$ in Acetonitrile (ACN) + 100 µL of 1 mM tetramethylethylene-diamine (tmeda) in dichloromethane (DCM) + 0.8 mL DCM.

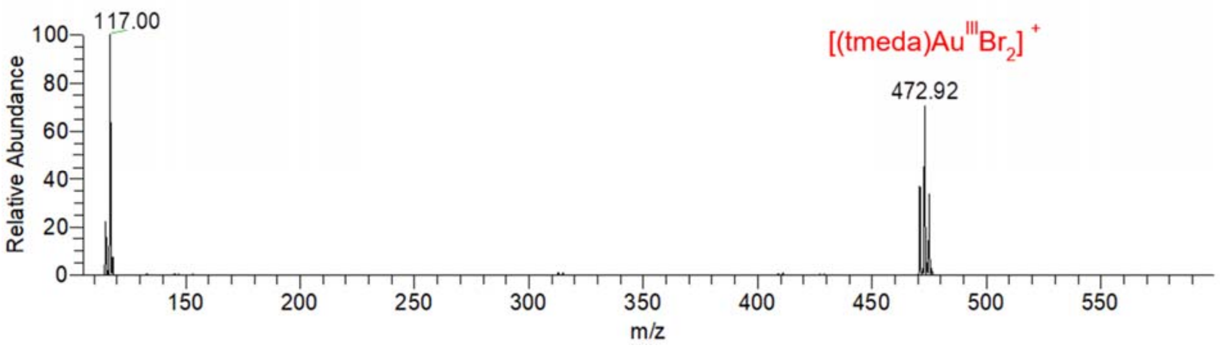

Figure S68: Source spectrum of Tetramethylethylenediamine with $AuBr_3$

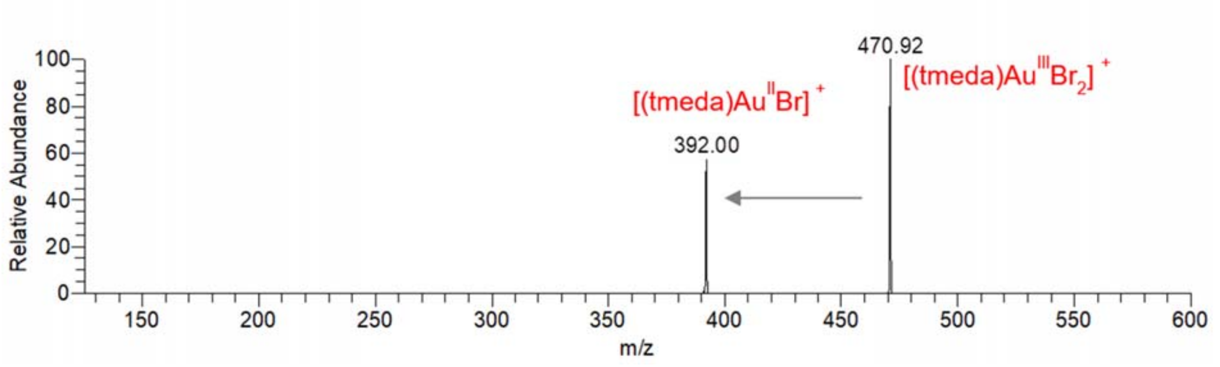

Figure S69: CID of *m/z* 471 at C.E = 13%

19) Bipyridine with $CuCl_2$

Sample preparation: 100 µL of 1 mM $CuCl_2$ in Acetonitrile (ACN) + 100 µL of 1 mM bipyridine (bipy) in dichloromethane (DCM) + 0.8 mL DCM.

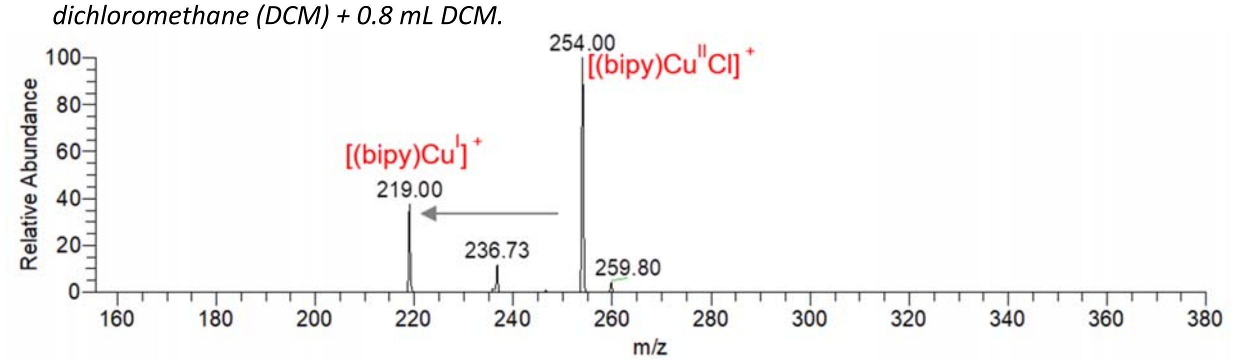

Figure S70: CID of *m/z* X at C.E = 32%

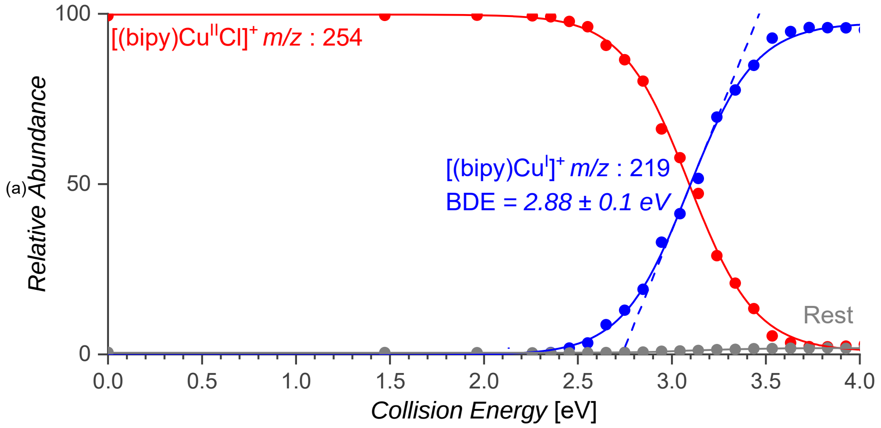

Figure S71: Energy Resolved CID of *m/z* 254

20) Bipyridine with $CuBr_2$

Sample preparation: 100 µL of 1 mM $CuBr_2$ in Acetonitrile (ACN) + 100 µL of 1 mM bipyridine (bipy) in dichloromethane (DCM) + 0.8 mL DCM.

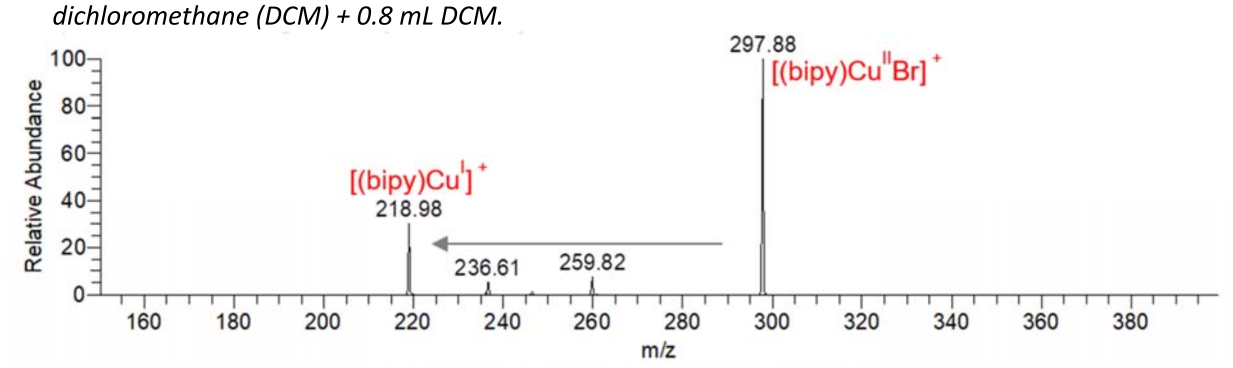

Figure S72: CID of *m/z* X at C.E = 25%

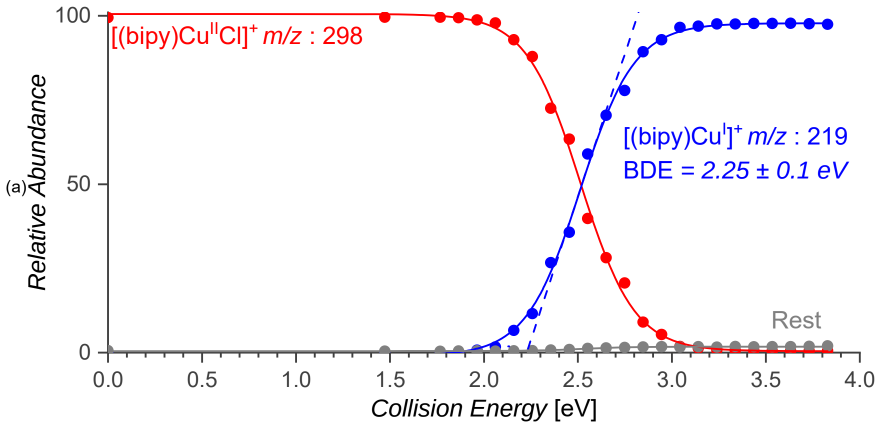

Figure S73: Energy Resolved CID of *m/z* 298

Synthesis of gold(III) complexes

Using the synthesis procedure described by Casini et al., gold(III) complexes of bipyridine, phenanthroline and terpyridine with Cl or Br and PF_6 as counterion were prepared.[7] To 1 eq. of the ligand in acetonitrile, aqueous solution of 1 eq. of $KAuX_4$ or AuX_3 was added and the mixture refluxed for 12-24 hrs. Followed by addition of 3 eq. of KPF_6 and refluxed for another 2-3 hours. The solution was cooled to room temperature and the precipitate was filtered, washed with cold water (3X) and dried under vacuum. These complexes were used for electrochemical studies.

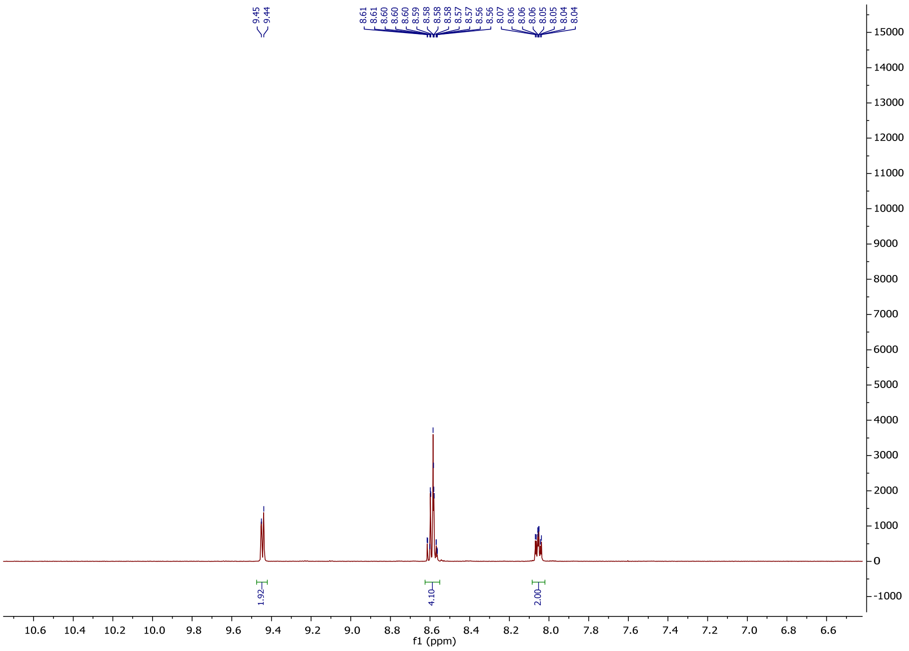

Figure S74: ^{1}H NMR in CD_3CN of gold(III) complex prepared with bipy, gold(III) chloride with PF_6 counterion $[Au(bipy)Cl_2]PF_6$

^{1}H NMR (500 MHz, CD_3CN) δ 9.47 – 9.42 (d, 2H), 8.63 – 8.55 (m, 4H), 8.10 – 8.01 (td, 2H).
NMR matches with the literature values of the complex.[8]

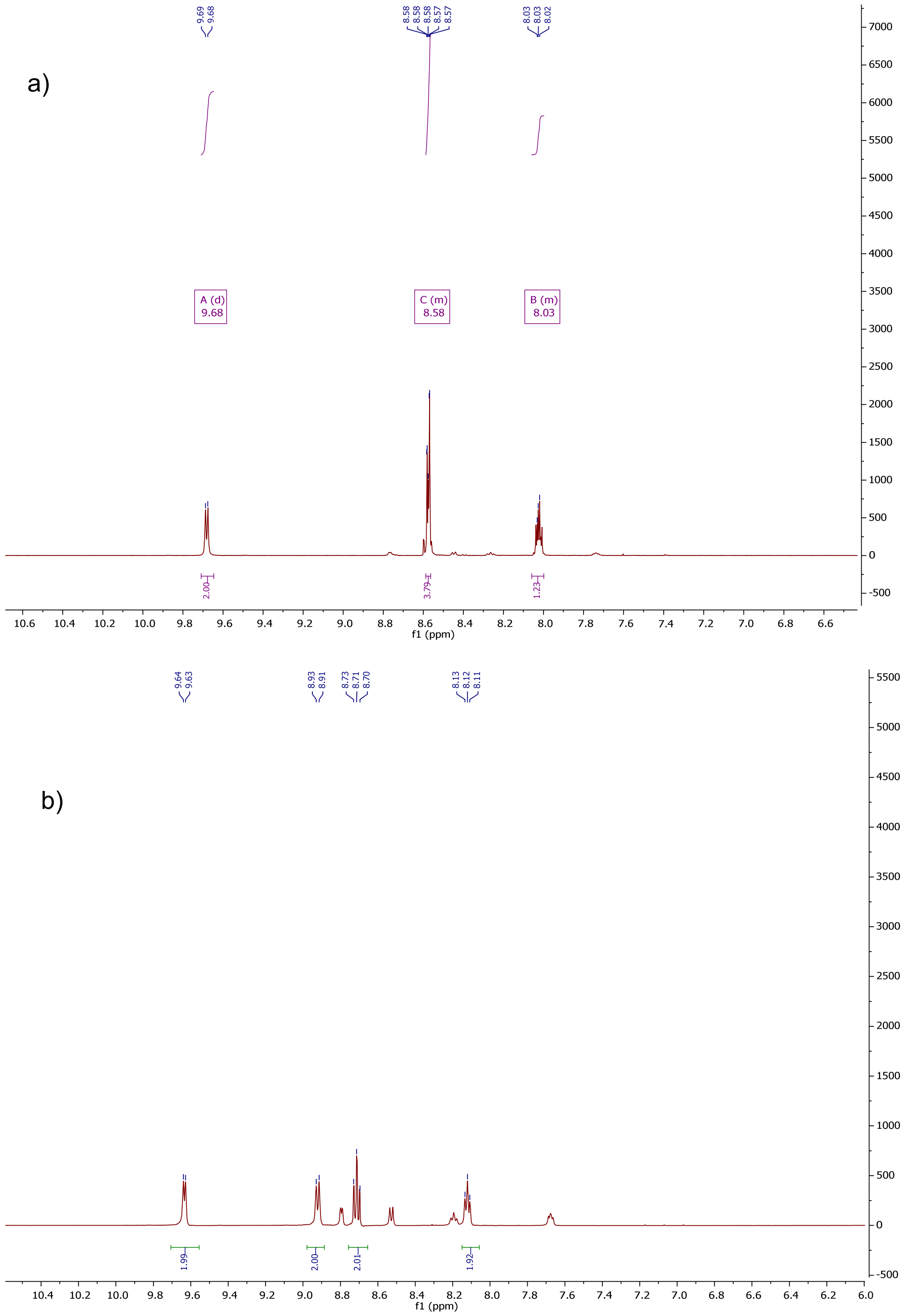

Figure S75: ^{1}H NMR of gold(III) complex prepared with bipy, gold(III) bromide with PF_6 counterion $[Au(bipy)Br_2]PF_6$ – a) CD_3CN and b) DMSO-d_6

^{1}H NMR (500 MHz, CD_3CN) δ 9.68 (d, J = 6.0 Hz, 2H), 8.59 – 8.54 (m, 4H), 8.09 – 7.98 (m, 1H).
1H NMR (500 MHz, DMSO) δ 9.64 – 9.63 (d, 2H), 8.93 – 8.91 (d, 2H), 8.73 – 8.70 (t, 2H), 8.13 – 8.11 (t, 2H).
As noted in the literature for similar gold(III) complexes, the low intensity signals are due to the degradation of the complex in DMSO-d_6.[9]

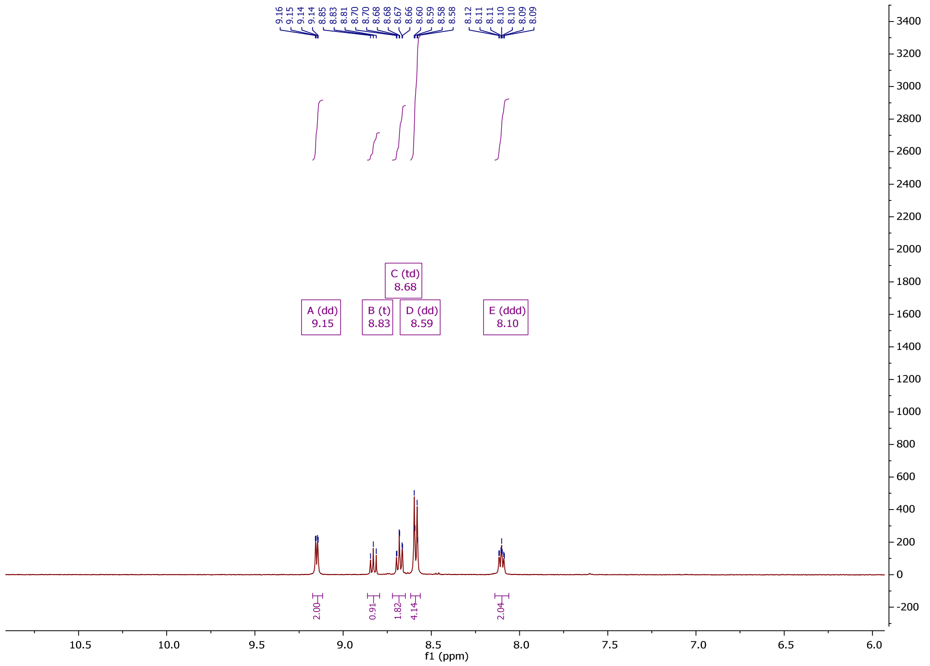

Figure S76: ^{1}H NMR in CD_3CN of gold(III) complex prepared with terpy, gold(III) chloride with PF_6 counterion $[Au(terpy)Cl](PF_6)_2$

^{1}H NMR (500 MHz, CD_3CN) δ 9.15 (dd, *J* = 6.0, 1.5 Hz, 2H), 8.83 (t, *J* = 8.2 Hz, 1H), 8.68 (td, *J* = 7.9, 1.5 Hz, 2H), 8.59 (dd, *J* = 8.1, 2.2 Hz, 4H), 8.10 (ddd, *J* = 7.7, 6.0, 1.6 Hz, 2H).
NMR compares well with the literature values of the same complex (shifts observed because of different solvents used).[9]

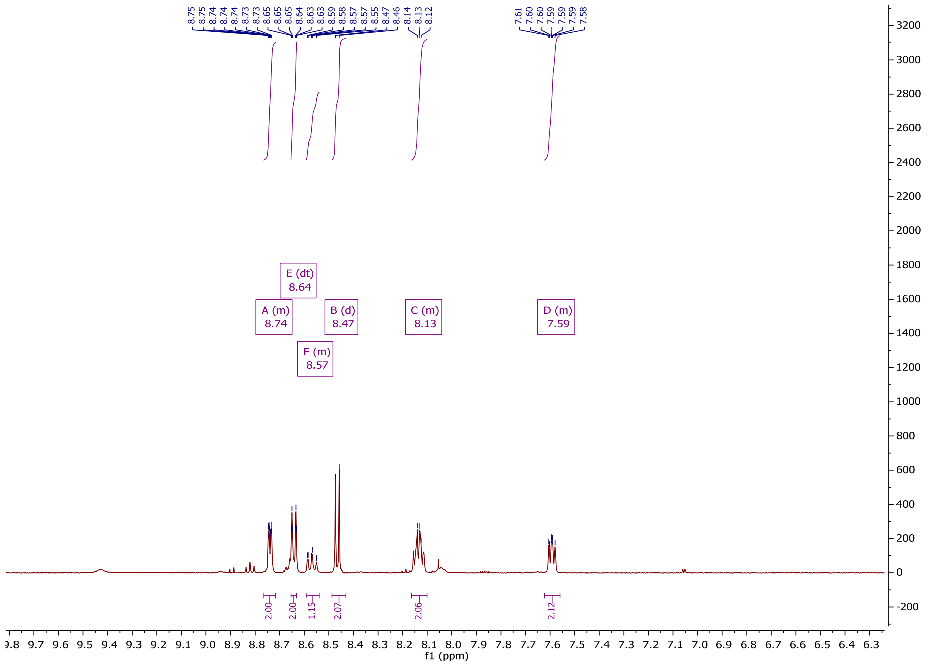

Figure S77: ^{1}H NMR in CD_3CN of gold(III) complex prepared with bipy, gold(III) bromide with PF_6 counterion $[Au(terpy)Br](PF_6)_2$

^{1}H NMR (500 MHz, CD_3CN) δ 8.76 – 8.72 (m, 2H), 8.64 (dt, *J* = 8.0, 1.1 Hz, 2H), 8.59 – 8.54 (m, 1H), 8.47 (d, *J* = 7.9 Hz, 2H), 8.16 – 8.10 (m, 2H), 7.62 – 7.56 (m, 2H).
As noted in the literature for similar gold(III) complexes, the low intensity signals are due to the degradation of the complex; we have also observed a precipitation overtime.[9]

Electrochemistry experiments

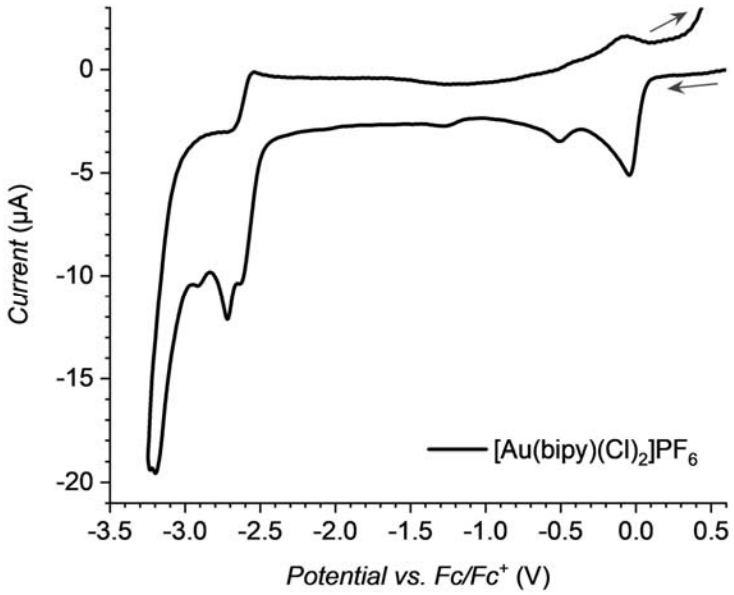

Figure S78: Cyclic voltammogram (CV) of [Au(bipy)(Cl)$_2$]PF$_6$ (0.25mM) measured in TBAPF$_6$ (0.1M) in DMF with a scan rate of 100 mV/s.

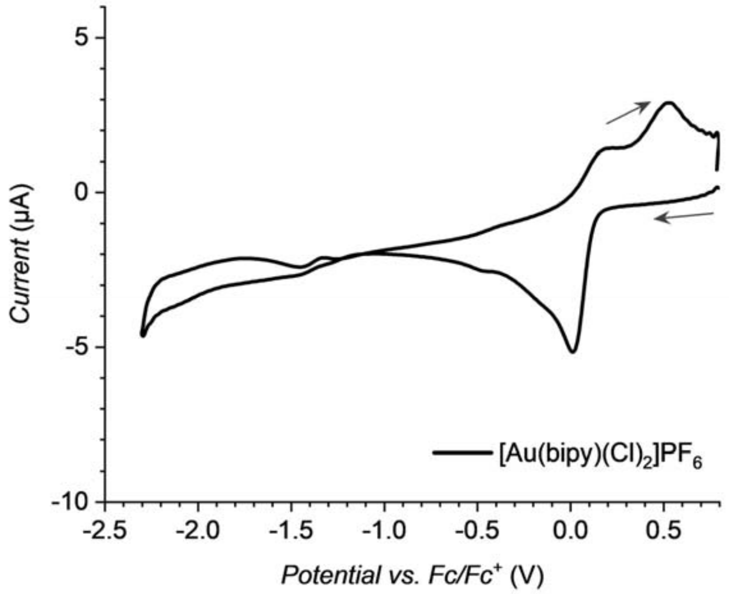

Figure S79: Cyclic voltammogram (CV) of [Au(bipy)(Cl)$_2$]PF$_6$ (0.25mM) measured in TBAPF$_6$ (0.1M) in

DCM with a scan rate of 100 mV/s.

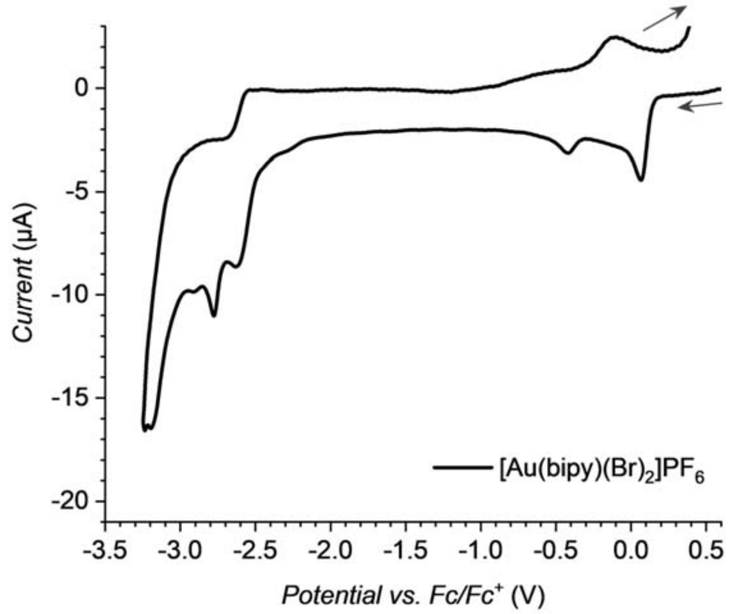

Figure S80: Cyclic voltammogram (CV) of $[Au(bipy)(Br)_2]PF_6$ (0.25mM) measured in $TBAPF_6$ (0.1M) in DMF with a scan rate of 100 mV/s.

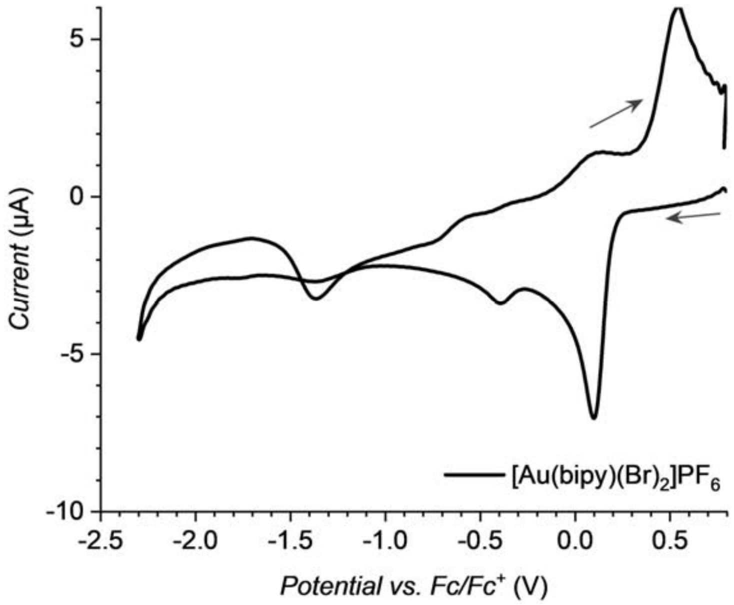

Figure S81: Cyclic voltammogram (CV) of $[Au(bipy)(Br)_2]PF_6$ (0.25mM) measured in $TBAPF_6$ (0.1M) in DCM with a scan rate of 100 mV/s.

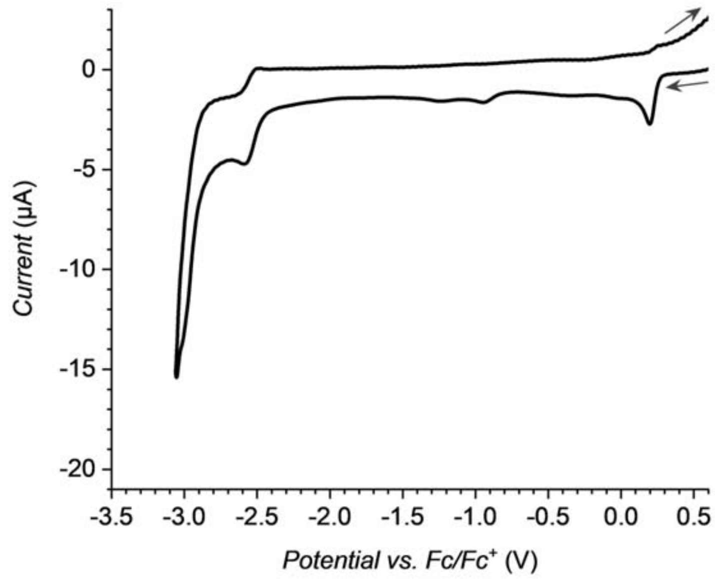

Figure S82: Cyclic voltammogram (CV) of [Au(terpy)(Cl)]$(PF_6)_2$ (0.25mM) measured in $TBAPF_6$ (0.1M) in DMF with a scan rate of 100 mV/s.

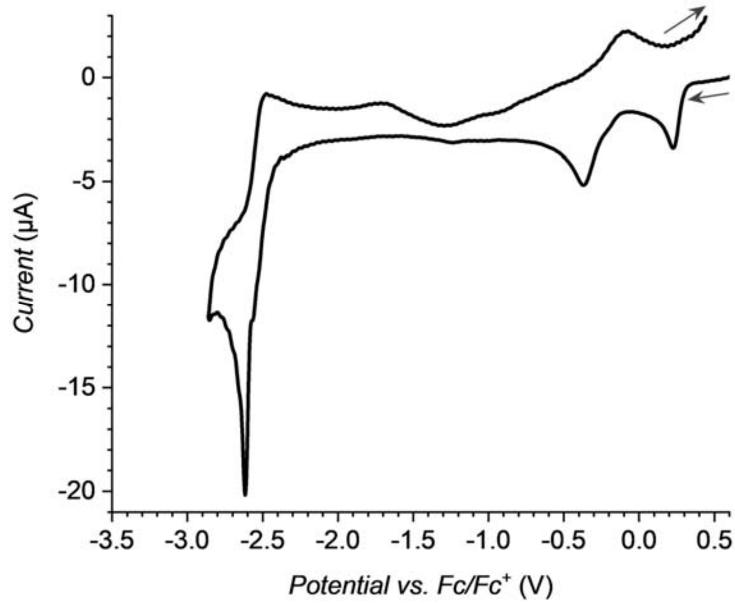

Figure S83: Cyclic voltammogram (CV) of [Au(terpy)(Br)]$(PF_6)_2$ (0.25mM) measured in $TBAPF_6$ (0.1M) in DMF with a scan rate of 100 mV/s.

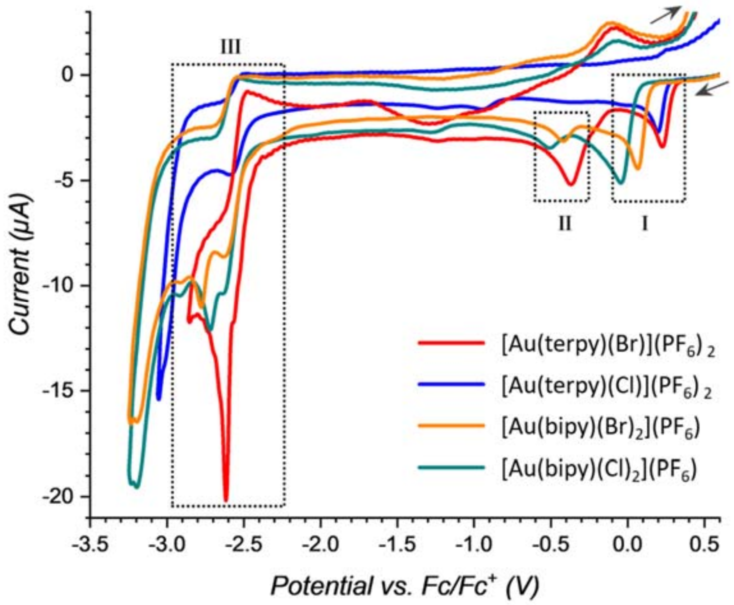

Figure S84: Overlaid cyclic voltammogram (CV) of [Au(bipy)(Cl)$_2$]PF$_6$ (dark cyan), [Au(bipy)(Br)$_2$]PF$_6$ (orange), [Au(terpy)(Cl)](PF$_6$)$_2$ (dark blue) and [Au(terpy)(Br)]PF$_6$)$_2$ (red) (0.25mM) measured in TBAPF$_6$ (0.1M) in DMF with a scan rate of 100 mV/s. Region I corresponds to the reduction of Au(III) → Au(I) of the corresponding gold(III) complexes.[10,11] The region II corresponds to the reduction of free Au(III) salt present in the complex, this is further confirmed by performing CV experiments with AuCl$_3$ and AuBr$_3$ salts (Figure S86). Region III includes the reduction of Au(I)→ Au(0) and the ligand reduction (the ligand reduction is shown in the figure S86).

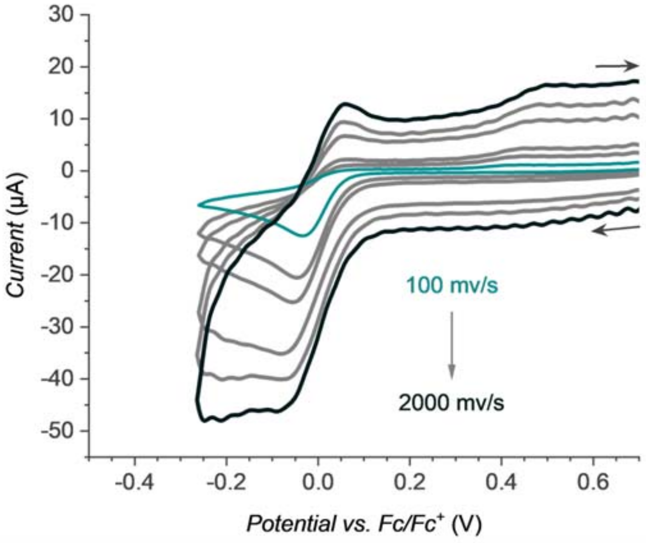

Figure S85: Cyclic voltammogram of [Au(bipy)(Cl)$_2$]PF$_6$ in DMF (0.5 mM) at various scan rates (100, 300, 500, 1000, 1500 and 2000 mV/s) in the range of the Au^{III} → Au^{I} reduction potential

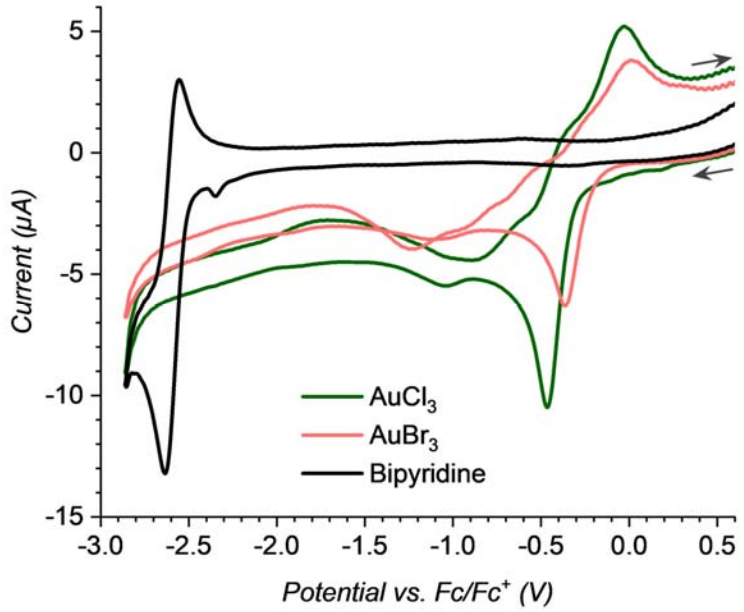

Figure S86: Cyclic voltammogram of gold salts $AuCl_3$, $AuBr_3$ and 2,2'-bipyridine ligand in DMF (0.5 mM) at a scan rate of 100 mV/s.

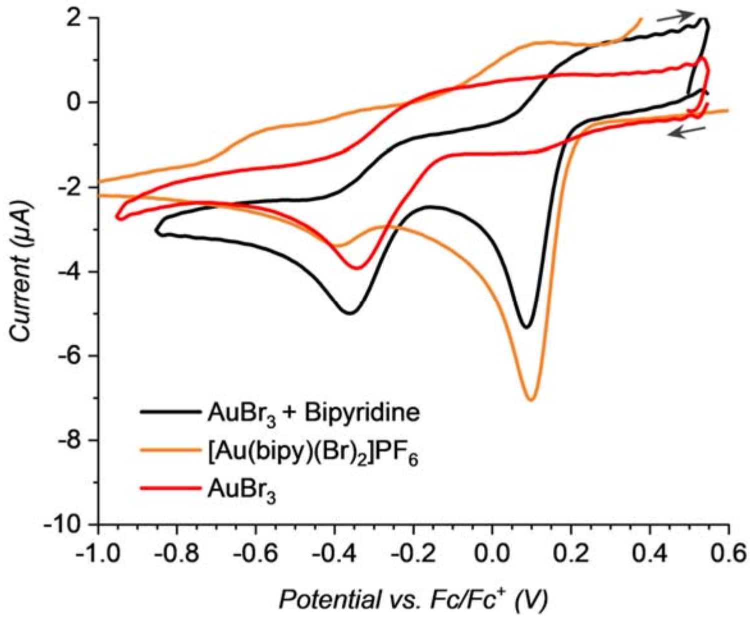

Figure S87: Cyclic voltammogram of $AuBr_3$ salt mixed with 2,2'-bipyridine (black), the synthesized complex $[Au(bipy)(Br)_2]PF_6$ (orange), and $AuBr_3$ salt (red) in DMF at a scan rate of 100 mV/s. The mixing of $AuBr_3$ with bipyridine also shows the same reduction peak of $[Au(bipy)(Br)_2]PF_6$ (synthesized separately) indicative of the formation same gold(III) complex.

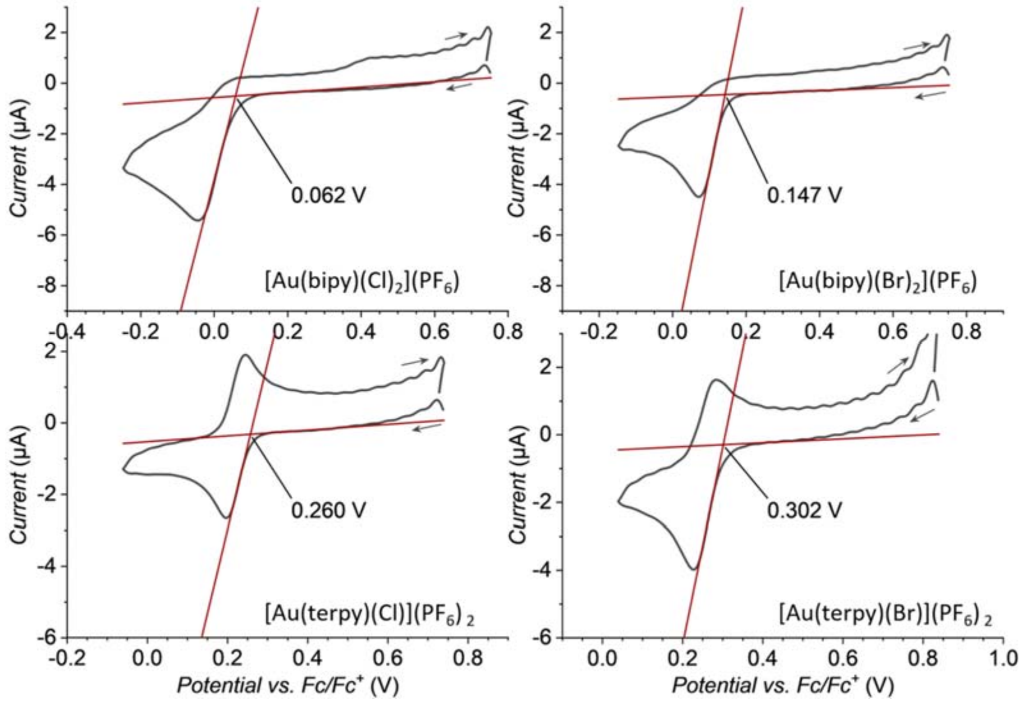

Figure S88: Calculation of the onset potentials using tangent line intersection from the cyclic voltammogram for gold(III) complexes in DMF (0.25 mM) at a scan rate of 100 mV/s.

IRPD and Vis spectra

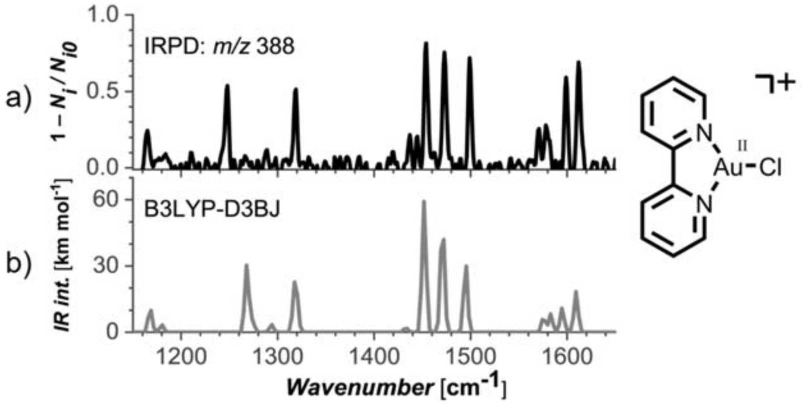

Figure S89: a) The experimental IR spectra of $[Au(bipy)(Cl)]^+$ measured by helium tagging photodissociation spectroscopy and b) Theoretically predicted IR spectra of $[Au(bipy)(Cl)]^+$ calculated by B3LYP-D3BJ/SDD, scaling factor: 0.98.

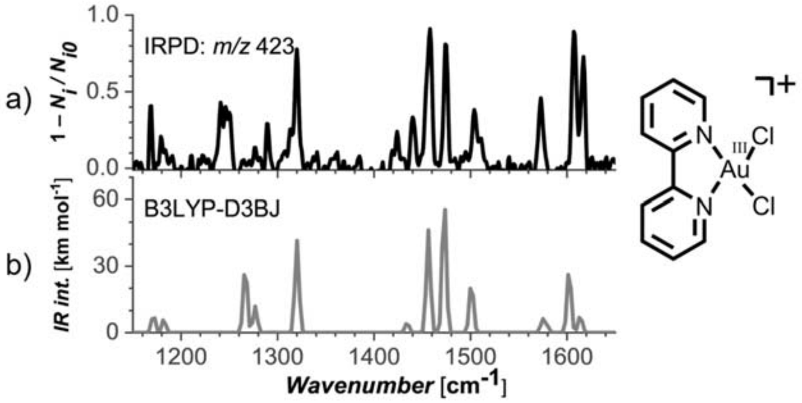

Figure S90: a) The experimental IR spectra of $[Au(bipy)(Cl)_2]^+$ measured by helium tagging photodissociation spectroscopy and b) Theoretically predicted IR spectra of $[Au(bipy)(Cl)_2]^+$ calculated by B3LYP-D3BJ/SDD, scaling factor: 0.98.

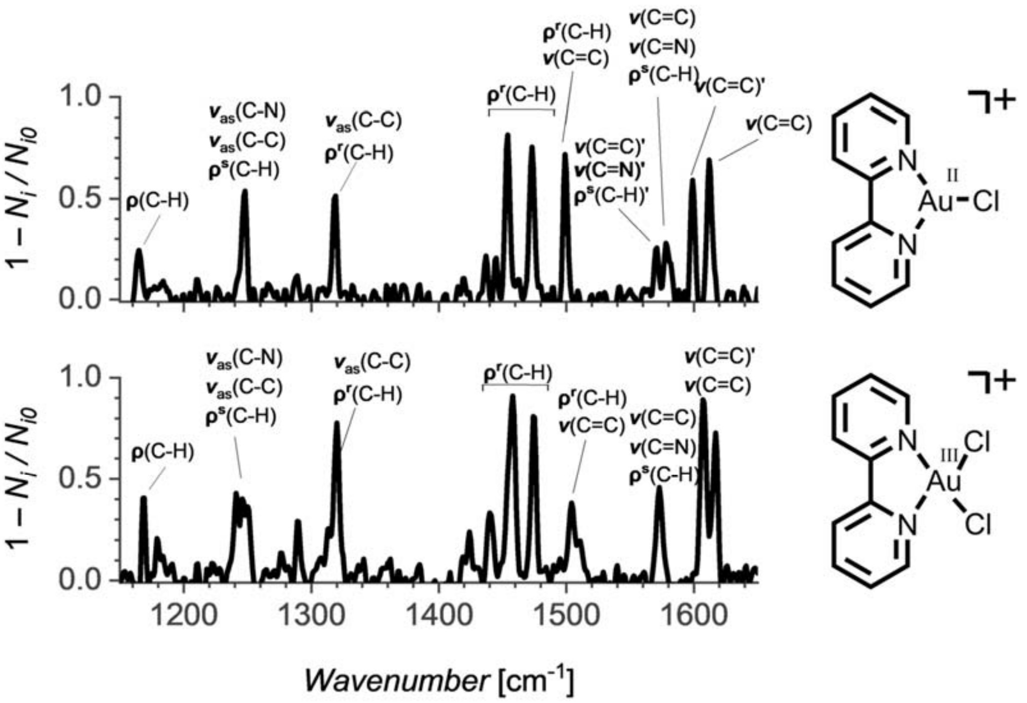

Figure S91: Experimental IR spectra comparison of the $[Au^{II}(bipy)Cl]^+$ and $[Au^{III}(bipy)Cl_2]^+$ with tentative assignment of the vibration modes. Note: ν is a stretching vibration, ν_{as} is an asymmetric stretching vibration, ρ is a general in-plane vibration, ρ^r is a rocking vibration and ρ^s is a scissoring vibration.

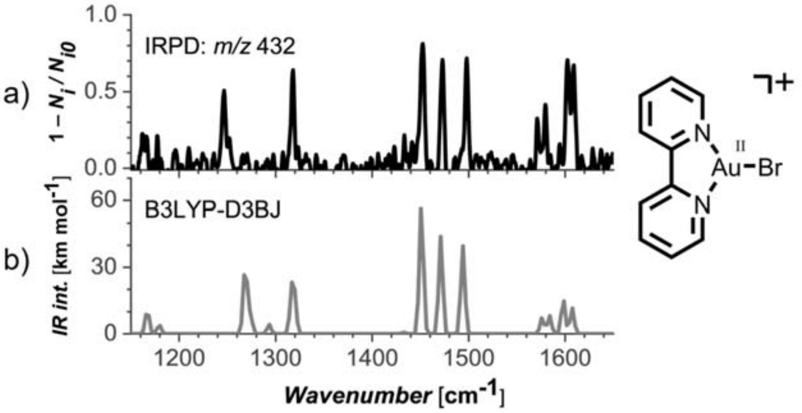

Figure S92: a) The experimental IR spectra of $[Au(bipy)(Br)]^+$ measured by helium tagging photodissociation spectroscopy. b) Theoretically predicted IR spectra of $[Au(bipy)(Br)]^+$ calculated by B3LYP-D3BJ/SDD, scaling factor: 0.98.

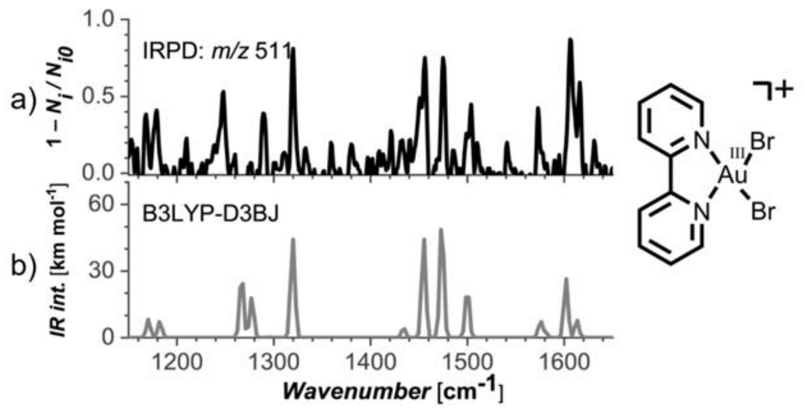

Figure S93: a) The experimental IR spectra of $[Au(bipy)(Br)_2]^+$ measured by helium tagging photodissociation spectroscopy. b) Theoretically predicted IR spectra of $[Au(bipy)(Br)_2]^+$ calculated by B3LYP-D3BJ/SDD, scaling factor: 0.98.

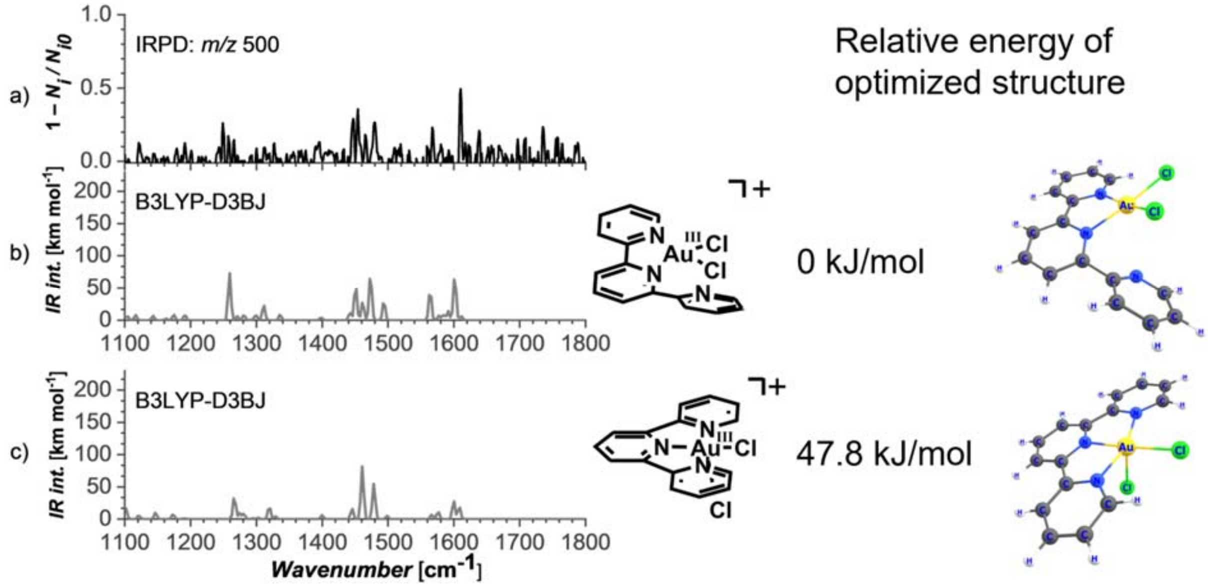

Figure S94: a) The experimental IR spectra of $[Au(terpy)(Cl)_2]^+$ measured by helium tagging photodissociation spectroscopy. b) Theoretically predicted IR spectra of $[Au(terpy)(Cl)_2]^+$ calculated by B3LYP-D3BJ/SDD, scaling factor: 0.98. c) Theoretically predicted IR spectra of $[Au(terpy)(Cl)_2]^+$ calculated by B3LYP-D3BJ/SDD, scaling factor: 0.98.

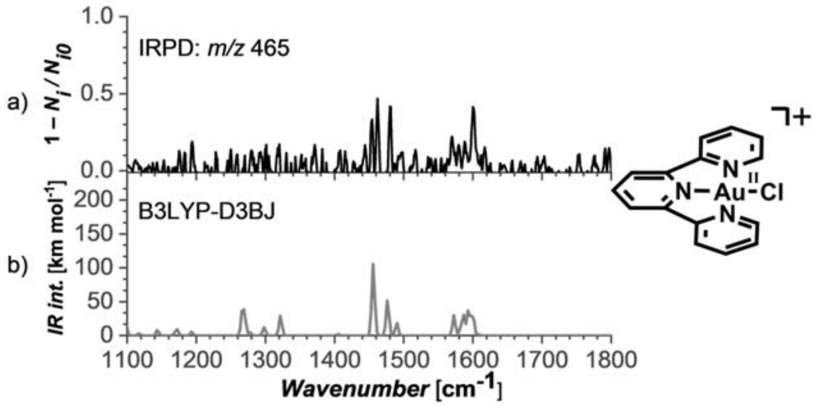

Figure S95: a) The experimental IR spectra of $[Au(terpy)(Cl)]^{+}$ measured by helium tagging photodissociation spectroscopy. b) Theoretically predicted IR spectra of $[Au(terpy)(Cl)]^{+}$ calculated by B3LYP-D3BJ/SDD, scaling factor: 0.98.

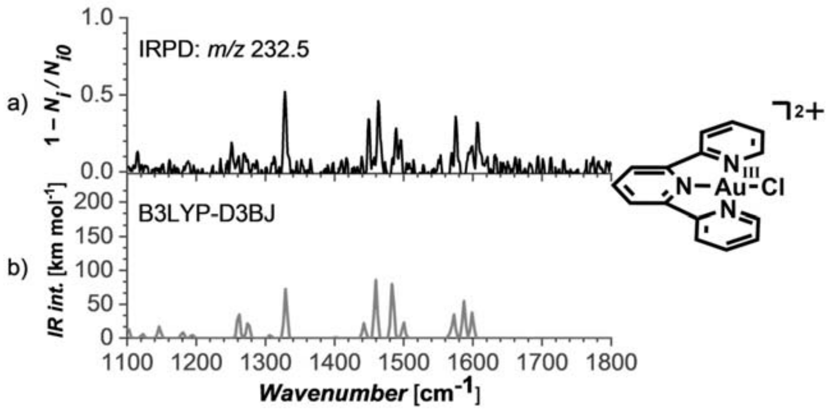

Figure S96: a) The experimental IR spectra of $[Au(terpy)(Cl)]^{2+}$ measured by helium tagging photodissociation spectroscopy. b) Theoretically predicted IR spectra of $[Au(terpy)(Cl)]^{2+}$ calculated by B3LYP-D3BJ/SDD, scaling factor: 0.98.

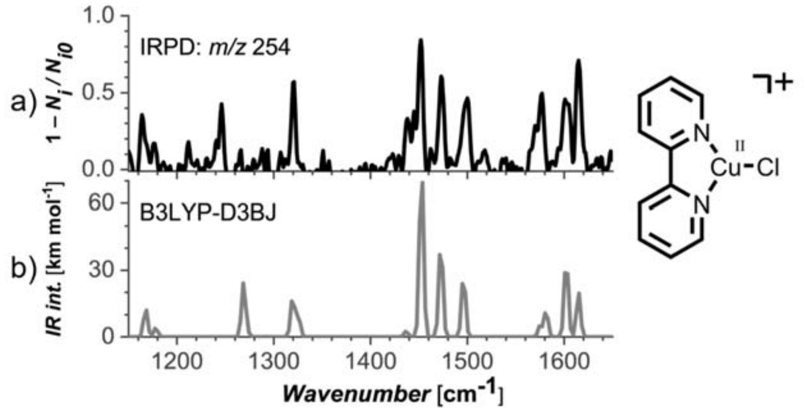

Figure S97: a) The experimental IR spectra of $[Cu(bipy)(Cl)]^+$ measured by helium tagging photodissociation spectroscopy and b) Theoretically predicted IR spectra of $[Cu(bipy)(Cl)]^+$ calculated by B3LYP-D3BJ/SDD, scaling factor: 0.98.

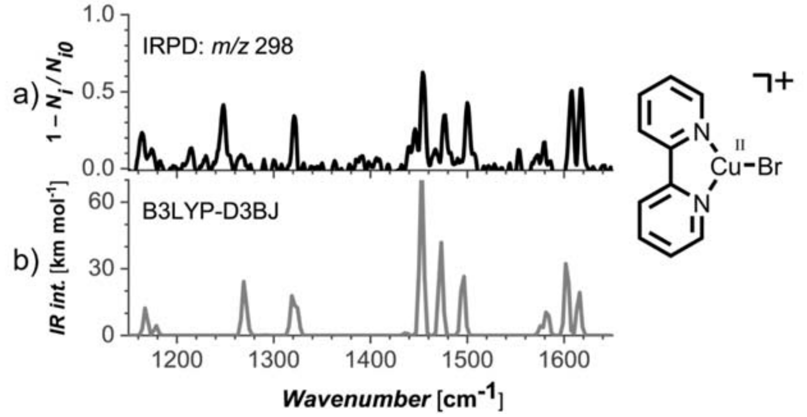

Figure S98: a) The experimental IR spectra of $[Cu(bipy)(Br)]^+$ measured by helium tagging photodissociation spectroscopy and b) Theoretically predicted IR spectra of $[Cu(bipy)(Br)]^+$ calculated by B3LYP-D3BJ/SDD, scaling factor: 0.98.

Helium tagging visPD measurements

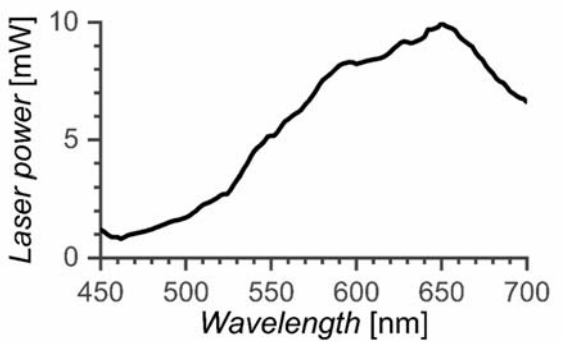

Figure S99: SuperK laser power dependence on the wavelength.

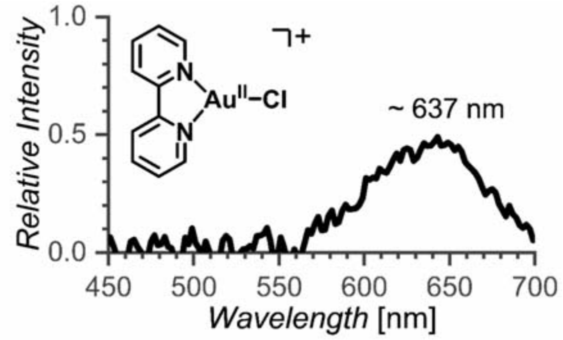

Figure S100: Visible spectra of $[Au(bipy)(Cl)]^{+}$ measured by helium tagging photodissociation spectroscopy.

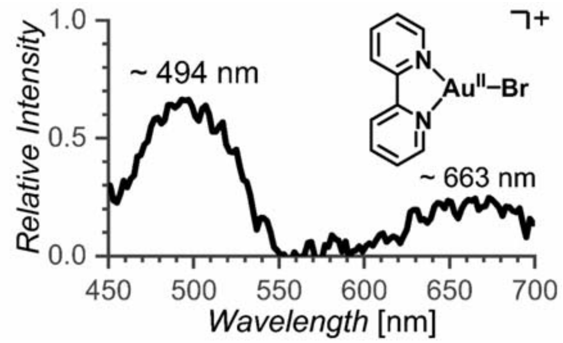

Figure S101: Visible spectra of $[Au(bipy)(Br)]^{+}$ measured by helium tagging photodissociation spectroscopy.

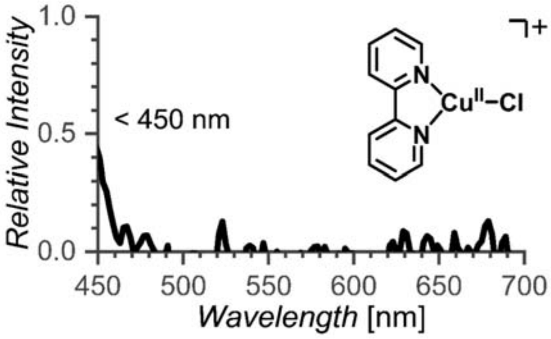

Figure S102: Visible spectra of $[Cu(bipy)(Cl)]^+$ measured by helium tagging photodissociation spectroscopy.

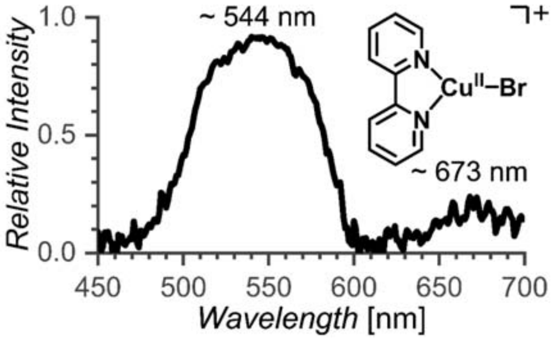

Figure S103: Visible spectra of $[Cu(bipy)(Br)]^+$ measured by helium tagging photodissociation spectroscopy.

Theoretical Details

Table S1. Overview of experimental and theoretical bond dissociation energies of $[Au(Lig)(X)_n]^+$ complexes (n = 1 or 2).

Reaction	BDE_{expt} [kJ mol^{-1}]	BDE_{theo} [kJ mol^{-1}]		
		B3LYP-D3/6-311+G** SDD on Au, Br and I	B3LYP-D3BJ/ Def2VTZPP	B3LYP-D3/6-311+G** Def2TZVPD on Au
$[Au^{III}(bipy)(Cl)_2]^+ \rightarrow [Au^{II}(bipy)(Cl)]^+ + Cl^{\cdot}$	213 ± 1	215	246	226
$[Au^{II}(bipy)(Cl)]^+ \rightarrow [Au^{I}(bipy)]^+ + Cl^{\cdot}$	228 ± 2	235	266	246
$[Au^{III}(bipy)(Br)_2]^+ \rightarrow [Au^{II}(bipy)(Br)]^+ + Br^{\cdot}$	169 ± 5	172	214	197
$[Au^{II}(bipy)(Br)]^+ \rightarrow [Au^{I}(bipy)]^+ + Br^{\cdot}$	196 ± 4	203	247	231
$[Au^{III}(bipy)(I)_2]^+ \rightarrow [Au^{II}(bipy)(I)]^+ + I^{\cdot}$	128 ± 2	146	123	
$[Au^{II}(bipy)(I)]^+ \rightarrow [Au^{I}(bipy)]^+ + I^{\cdot}$	178 ± 2	197	230	
$[Au^{III}(terpy)(Cl)_2]^+ \rightarrow [Au^{II}(terpy)(Cl)]^+ + Cl^{\cdot}$	155 ± 3	125		136
$[Au^{II}(terpy)(Cl)]^+ \rightarrow [Au^{I}(terpy)]^+ + Cl^{\cdot}$	226 ± 1	224		232
$[Au^{III}(terpy)(Br)_2]^+ \rightarrow [Au^{II}(terpy)(Br)]^+ + Br^{\cdot}$	108 ± 6	86		114
$[Au^{II}(terpy)(Br)]^+ \rightarrow [Au^{I}(terpy)]^+ + Br^{\cdot}$	191 ± 1	188		209

Method calibration for electronic spectra

In here, we tested performance of several functionals, *ab initio* methods, different basis sets, ECPs, role of the optimized structure, and spin-orbit effects in the calculations of electronic spectra of studied systems. Optimized structure is an important factor here. For example, the calculated $\lambda(a_1)$ in $[Au(bipy)(Br)]^+$ covers a relatively broad range of wavelengths, 639-723 nm (1.72-1.94 eV) depending on the method used for the geometry optimization. The expected dependence on DFT is mild. The tested functionals (B3LYP, CAM-B3LYP, PBE0, wB97DX) predict results from 440 nm to 464 nm (2.67-2.82 eV; **Table S2**) for $\lambda(a_1)$ in $[Au(bipy)(Br)]^+$. The aug-cc-pVTZ quality basis sets with the corresponding pseudopotentials (PP) provide results that converge within a few nm (**Table S3**) and are affordable for studied systems. Inclusion of scalar relativistic effects via ECP (effective core pseudopotential) is a necessity for Au atom but has only marginal effect for Br atom (**Table S2**). Using ECP on Cu atom may to lead to larger deviations and problems, **Table S3**. The effect of spin-orbit (SO) coupling can be relatively large, several tens of nm, **Tables S2 and S5**. For the qualitative analysis in the main text, we decided to use PBE0/def2TZVPP/PP(Au) for geometry and CAM-B3LYP/aug-cc-pVTZ/Au(PP) for excitation energies.

Table S2. Comparison of theoretical and experimental excitation bands for $[Au(bipy)(Br)]^+$.

**Geometry**	**Functional**	**Basis set and ECP**	**λ(*b*$_2$)/nm**	**λ(*a*$_1$)/nm**
B3LYP/6-311+G**/SDD(Au)	B3LYP	aug-cc-pVTZ/PP(Au,Br)	530	713
B3LYP/6-311+G**/SDD(Au,Br)		aug-cc-pVTZ/PP(Au,Br)	558	723
TPSS/def2-TZVP/PP(Au)		aug-cc-pVTZ/PP(Au,Br)	497	667
PBE0/def2-TZVPP/PP(Au)		aug-cc-pVTZ/SDD(Au,Br)	475	639
		6-311+G*/SDD(Au, Br)	476	647
		aug-cc-pVTZ/PP(Au,Br)	480	638
	B3LYP	aug-cc-pVTZ/PP(Au)	477	637
	**CAM-B3LYP**	**aug-cc-pVTZ/PP(Au)**	**495**	**664**
	PBE0	aug-cc-pVTZ/PP(Au)	482	638
	wB79xD	aug-cc-pVTZ/PP(Au)	500	672
	CAM-B3LYP	ZORA/TZP	479	634
	CAM-B3LYP	SO-ZORA/TZP	434	621
TPSS/def2-TZVP/Au(PP)	ADC(2)	def2-TZVP	554	713
TPSS/def2-TZVP/Au(PP)	CC2	def2-TZVP	510	646
**Experiment**			**494**	**663**

Table S3. Comparison of theoretical and experimental excitation bands for $[Cu(bipy)(Cl)]^+$.

**Geometry**	**Functional**	**Basis set and ECP**	**λ(*b*$_2$)/nm**	**λ(*a*$_1$)/nm**
B3LYP/6-311G**	B3LYP	aug-cc-pVTZ	484	681
PBE0/def2-TZVPP		auc-cc-pVTZ	464	658
		6-311+G**	487	697
		auc-cc-pVTZ	464	658
		aug-cc-pVQZ	464	654
		aug-cc-pVTZ/SDD(Cu)	**333**	**409**
PBE0/def2-TZVPP	B3LYP	aug-cc-pVTZ/PP(Cu)	435	654
	CAM-B3LYP	aug-cc-pVTZ/PP(Cu)	413	674
	**CAM-B3LYP**	**aug-cc-pVTZ**	**455**	**684**
	PBE0	aug-cc-pVTZ	443	686
	wB79XD	aug-cc-pVTZ	440	692
TPSS/def2TZVP	ADC(2)	def2-TZVP	556	898
TPSS/def2TZVP	CC2	def2-TZVP	558	617
**Experiment**			**<450**	**-**

Table S4. Comparison of theoretical and experimental excitation bands for [Cu(bipy)(Br)]$^{+}$.

Geometry	Functional	Basis set and ECP	λ(b_2)/nm	λ(a_1)/nm
B3LYP/6-311G**	B3LYP	aug-cc-pVTZ		
B3LYP/6-311G**/SDD(Cu)		aug-cc-pVTZ		
PBE0/def2-TZVPP		auc-cc-pVTZ	532	680
PBE0/def2-TZVPP		6-311+G**		
		auc-cc-pVTZ		
		aug-cc-pVQZ	532	669
		aug-cc-pVTZ/SDD(Cu)	**413**	**1095**
	B3LYP	aug-cc-pVTZ/PP(Cu)		
	CAM-B3LYP	aug-cc-pVTZ/PP(Cu)	497	691
	**CAM-B3LYP**	**aug-cc-pVTZ**	545	694
	PBE0	aug-cc-pVTZ	520	699
	wB79XD	aug-cc-pVTZ	534	699
	M06-2X	aug-cc-pVTZ/PP(Au)	**368**	**991**
TPSS/def2-TZVP	ADC(2)	def2-TZVP	**785**	**968**
TPSS/def2-TZVP	CC2	def2-TZVP	**681**	**1097**
**Experiment**			**544**	**673**

Table S5. Comparison of theoretical and experimental excitation bands for [Au(bipy)(Cl)]$^{+}$ using the C_s structure.

Structure	Functional	Basis set and ECP	λ(*a*")/nm	λ(*a*')/nm
B3LYP/6-311+G**/SDD(Au)	B3LYP	aug-cc-pVTZ/PP(Au)	515	750
PBE0/def2-TZVPP/PP(Au)		aug-cc-pVTZ/PP(Au)	457	693
		6311+G**/SDD(Au)	472	717
		aug-cc-pVTZ/SDD(Au)	464	705
	B3LYP	aug-cc-pVTZ/PP(Au)	457	693
	**CAM-B3LYP**	**aug-cc-pVTZ/PP(Au)**	**469**	**665**
	PBE0	aug-cc-pVTZ/PP(Au)	451	677
	wB97DX	aug-cc-pVTZ/PP(Au)	469	674
	M06-2X	aug-cc-pVTZ/PP(Au)	485	767
	CAM-B3LYP	ZORA/TZP	464	708
	CAM-B3LYP	SO-ZORA/TZP	408	646
TPSS/def2-TZVP/Au(PP)	ADC(2)	def2-TZVP	461	697
TPSS/def2-TZVP/Au(PP)	CC2	def2-TZVP	440	633
**Experiment**			**-**	**637**

Table S6. Dependence of electronic energy and calculated excitation energies (intensities in parenthesis) on N-Au-Br in-plane bending in $[Au(bipy)(Br)]^+$.

N-Au-Br(deg)	E(kJ/mol)	λ (*a*")/nm	λ (*a*')/nm
147	0		664(0.0001)
137	0.04	514(0.1468)	706(0.0047)
127	0.63	512(0.0707)	754(0.0194)
117	2.21	508(0.0320)	809(0.0200)

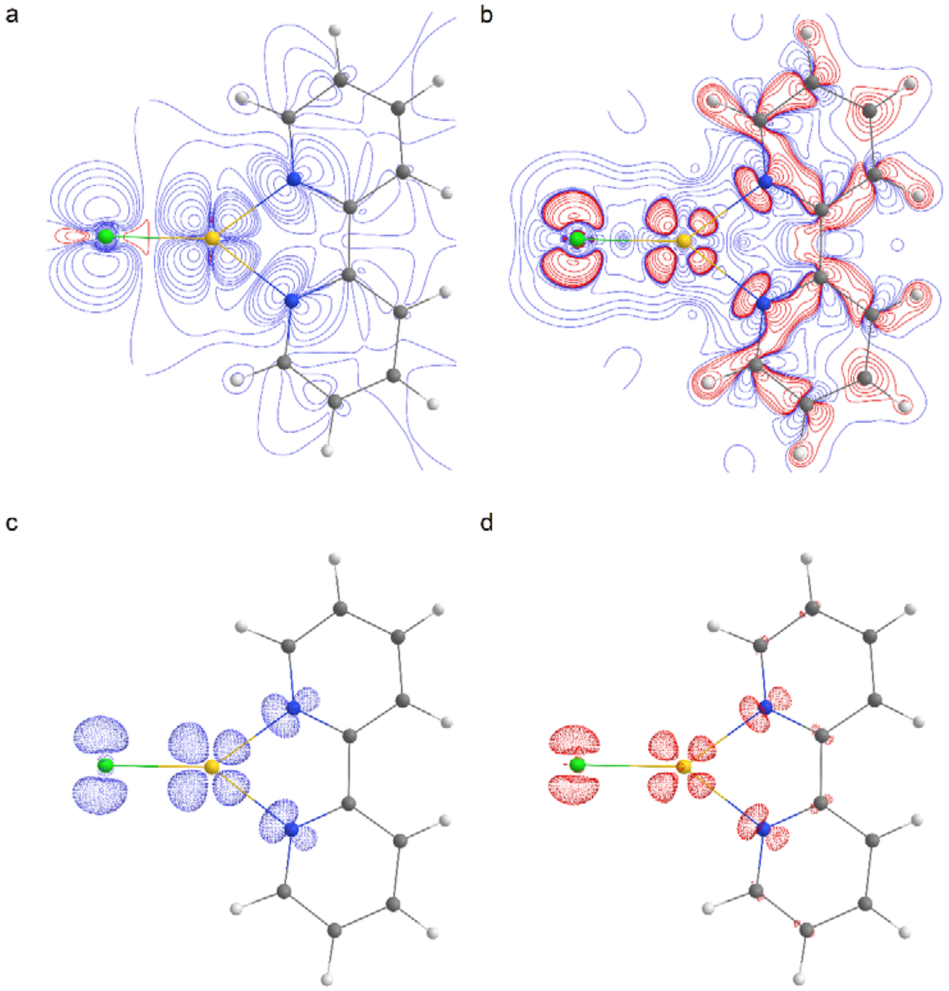

Figure S104: Profiles of (a) the spin density and (b) the Laplacian of the spin density on the plane of the [Au(bipy)(Cl)]$^+$ complex. (c) The isosurfaces of (c) the spin density at 0.01 and (d) the Laplacian of the spin density at -0.05 au. Positive and negative values are presented by blue and red in all maps. Negative values in the Laplacian plots denote regions in with spin density is more concentrated.

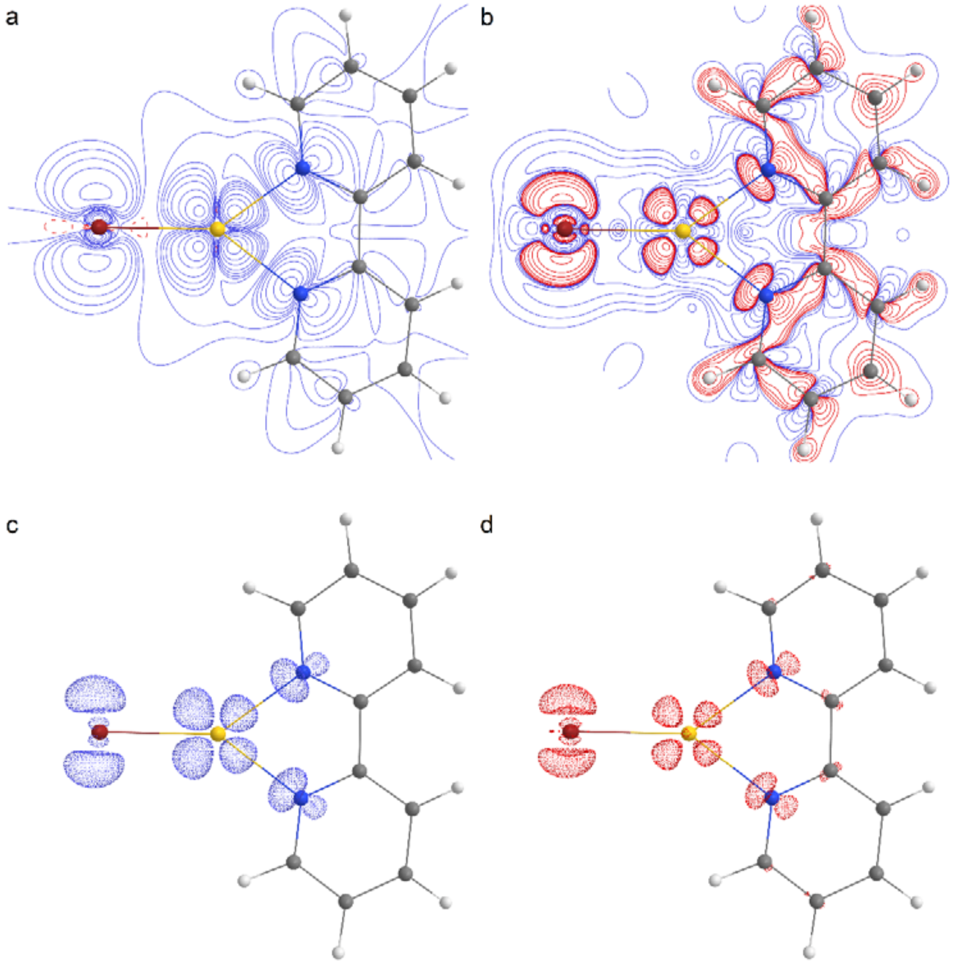

Figure S105: Profiles of (a) the spin density and (b) the Laplacian of the spin density on the plane of the $[Au(bipy)(Br)]^{+}$ complex. (c) The isosurfaces of (c) the spin density at 0.01 and (d) the Laplacian of the spin density at -0.05 au. Positive and negative values are presented by blue and red in all maps. Negative values in the Laplacian plots denote regions in with spin density is more concentrated.

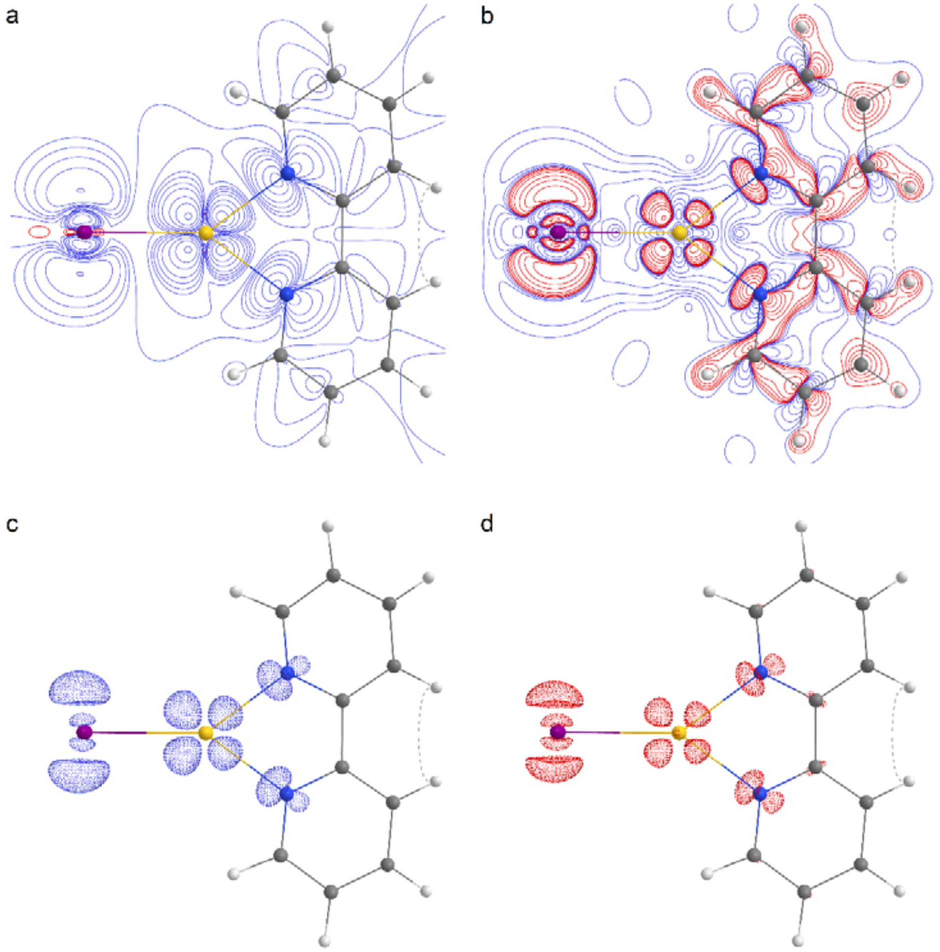

Figure S106: Profiles of (a) the spin density and (b) the Laplacian of the spin density on the plane of the $[Au(bipy)(I)]^+$ complex. (c) The isosurfaces of (c) the spin density at 0.01 and (d) the Laplacian of the spin density at -0.05 au. Positive and negative values are presented by blue and red in all maps. Negative values in the Laplacian plots denote regions in with spin density is more concentrated.

References

[1] P. Motloch, J. Jašík, J. Roithová, *Organometallics* **2021**, *40*, 1492–1502.

[2] J. Roithová, A. Gray, E. Andris, J. Jašík, D. Gerlich, *Acc. Chem. Res.* **2016**, *49*, 223–230.

[3] J. Jašík, R. Navrátil, I. Němec, J. Roithová, *J. Phys. Chem. A* **2015**, *119*, 12648–12655.

[4] E.-L. Zins, C. Pepe, D. Schröder, *J. Mass Spectrom.* **2010**, *45*, 1253–1260.

[5] J. E. Carpenter, C. P. McNary, A. Furin, A. F. Sweeney, P. B. Armentrout, *J. Am. Soc. Mass Spectrom.* **2017**, *28*, 1876–1888.

[6] R. Rahrt, T. Auth, M. Demireva, P. B. Armentrout, K. Koszinowski, *Anal. Chem.* **2019**, *91*, 11703–11711.

[7] A. Casini, M. C. Diawara, R. Scopelliti, S. M. Zakeeruddin, M. Grätzel, P. J. Dyson, *J. Chem. Soc. Dalt. Trans.* **2010**, *39*, 2239–2245.

[8] C. E. Rezsnyak, J. Autschbach, J. D. Atwood, S. Moncho, *J. Coord. Chem.* **2013**, *66*, 1153–1165.

[9] K. Czerwińska, M. Golec, M. Skonieczna, J. Palion-Gazda, D. Zygadło, A. Szlapa-Kula, S. Krompiec, B. Machura, A. Szurko, *Dalt. Trans.* **2017**, *46*, 3381–3392.

[10] M. C. Gimeno, J. M. López-de-Luzuriaga, E. Manso, M. Monge, M. E. Olmos, M. Rodríguez-Castillo, M.-T. Tena, D. P. Day, E. J. Lawrence, G. G. Wildgoose, *Inorg. Chem.* **2015**, *54*, 10667–10677.

[11] U. Koelle, A. Laguna, *Inorganica Chim. Acta* **1999**, *290*, 44–50.
